# A nitrogen-base catalyzed generation of organotin(ii) hydride from an organotin trihydride under reductive dihydrogen elimination†
†Electronic supplementary information (ESI) available: Detailed information concerning the general synthetic procedures, NMR spectroscopy and crystallographic details. Detailed explanations on the kinetic measurements and selected 1D NMR spectra as well as details on the computational considerations. CCDC 1061347–1061349. For ESI and crystallographic data in CIF or other electronic format see DOI: 10.1039/c5sc01561h



**DOI:** 10.1039/c5sc01561h

**Published:** 2015-05-22

**Authors:** Christian P. Sindlinger, Andreas Stasch, Holger F. Bettinger, Lars Wesemann

**Affiliations:** a Institut für Anorganische Chemie , Auf der Morgenstelle 18 , 72076 Tübingen , Germany . Email: lars.wesemann@uni-tuebingen.de; b School of Chemistry , Monash University , PO Box 23 , Melbourne , VIC 3800 , Australia; c Institut für Organische Chemie , Auf der Morgenstelle 18 , 72076 Tübingen , Germany

## Abstract

Since their first description over a decade ago, organotin(ii) hydrides have been an iconic class of compounds in molecular main group chemistry. Among other approaches they have been accessed from the hydrogenation of distannynes. We herein report their accessibility from the other direction by dehydrogenation of organotin trihydride. On reacting pyridine and amine bases with the bulky substituted organotin trihydride Ar*SnH_3_ (Ar* = 2,6-trip_2_(C_6_H_3_)–, trip = 2,4,6-triisopropylphenyl) hydrogen evolution was observed. In case of catalytic amounts of base the dehydro-coupling product diorganodistannane Ar*H_2_SnSnH_2_Ar* was obtained quantitatively whilst for excessive amounts (>4 eq.) the monomeric base adduct to known Ar*SnH was obtained almost exclusively. The base adducts were found to be remarkably thermally robust. They readily react with polar fulvenic C=C-bonds in hydro-stannylenylation reactions. The resulting half-sandwich complex Ar*SnCp* was structurally characterized. Moreover, on application of less nucleophilic amine bases, the uncoordinated, in solution dimeric [Ar*SnH]_2_ is formed. NMR spectroscopic studies on the kinetics of the DMAP-catalysed reductive elimination of dihydrogen were performed. The activation energy was approximated to be 13.7 kcal mol^–1^. Solvent dependencies and a kinetic isotope effect KIE of *k*_H_/*k*_D_ = 1.65 in benzene and 2.04 in THF were found and along with DFT calculations support a polar mechanism for this dehydrogenation.

## Abstract 2

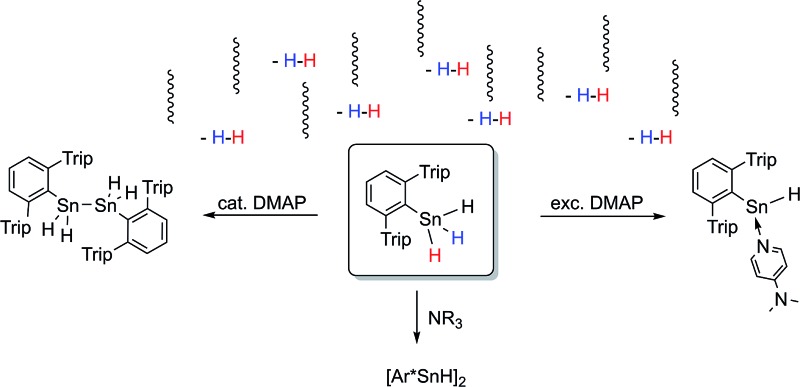
Amine bases are shown to induce reductive elimination of dihydrogen from terphenyltin trihydride.

## Introduction

In the periodic table, tin is located at an intriguing borderline position in terms of the stability of its di- and tetravalent derivatives. As the fifth period representative of the carbon group 14 it thermodynamically prefers formation of tetravalent compounds in the oxidation state +4 yet it benefits from the markedly increased effect of the inert pair for heavier elements. Whilst divalent compounds of its lighter congener Ge are easily oxidized toward Ge(iv) species, inorganic Pb(iv) compounds are prone to reduction to form the preferred divalent species. Located between these two extremes tin forms stable di- and tetravalent salts and thus reveals a more sophisticated relationship between di- and tetravalency.

**Scheme 1:**
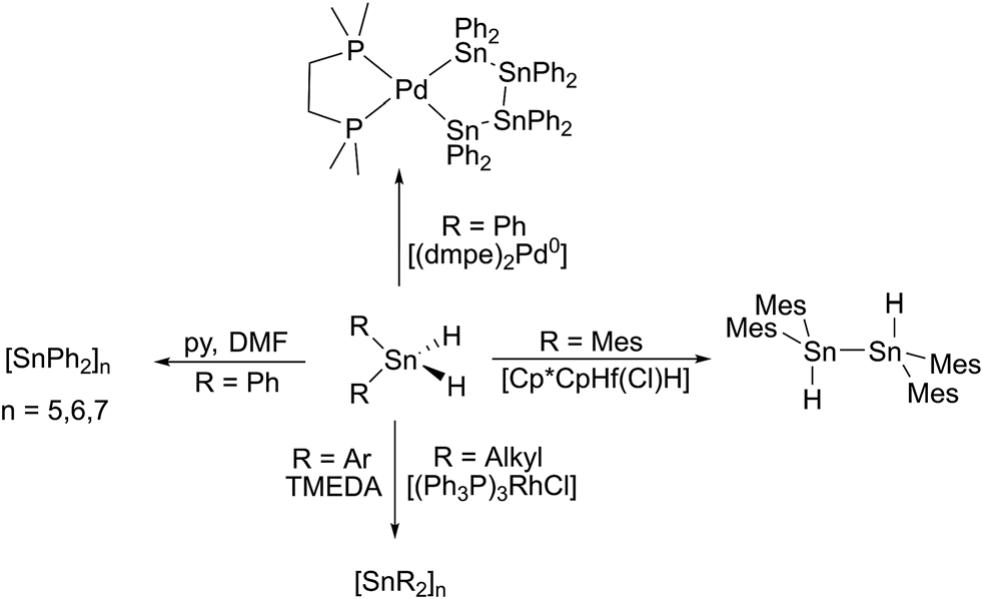
Selected examples for dehydrogenation approaches toward organotin dihydrides.

In the field of organometallic chemistry the preparation and handling of divalent compounds of Sn (stannylenes) has always been a challenging task due to their generally high sensitivity toward ambient oxidative atmosphere.^1,2^ Stannylenes are usually accessed by salt metathesis reactions with the respective Sn(ii) halides or by various reduction approaches of the diorganotin dihalides.^3^ A highly underestimated class of potential precursors for low-valent tin chemistry are tetravalent hydrides of the general composition R_*n*_SnH_4–*n*_ with *n* ≤ 2. Owing to the weak tin–hydrogen bond these compounds represent potential precursors for the generation of divalent Sn(ii) species by removal of dihydrogen. Observations of a catalytic approach towards evolution of dihydrogen from tin dihydrides date back to seminal contributions of Kuivila *et al.* and Neumann *et al.* in the early 1960s who reported on formamide and base catalysed dehydrogenation from diphenyltin dihydride (and other diorganotin dihydrides) and isolation of yellow solids that were characterized as polymeric or oligomeric diorganotin.^4–16^ Many years later the concept was revisited and also transition metal catalysts have been applied to perform selective dehydrocoupling reactions with tin dihydrides to obtain tetraorganodistannanes or organotin polymers (Scheme 1).^17–23^ In the case of the formation of tetraorganodistannanes the formation *via* intermediate generation of the stannylene was proposed.^17^

**Chart 1:**
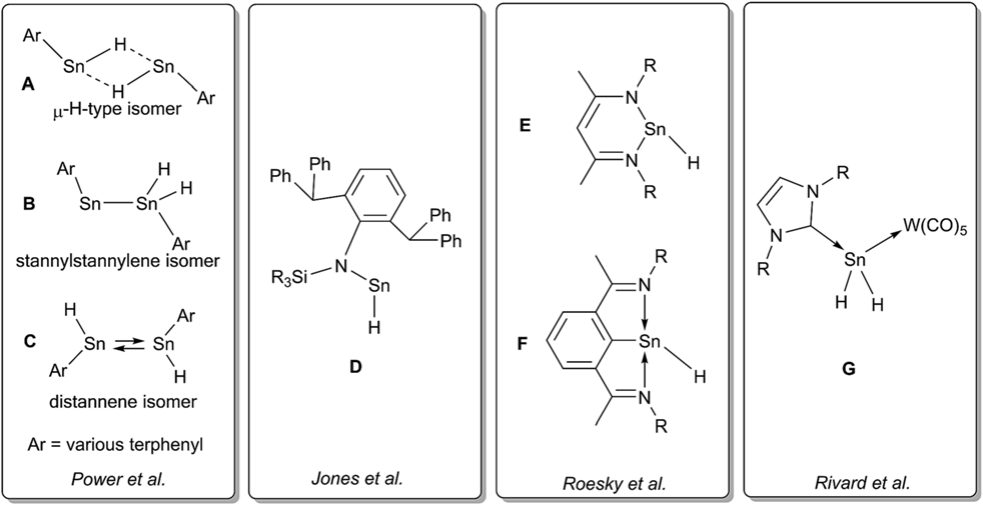
Selected examples for tin(ii) hydrides.

Recently, we have been able to show that the use of N-heterocyclic carbenes as stoichiometric dehydrogenation reagents represents a highly selective way to abstract dihydrogen from tin di- and trihydrides.^24^ Depending on the exact stoichiometric ratio of the carbene *vs.* tin hydride either dehydrocoupling was achieved or the adducts of carbenes to stannylenes and organotin(ii) hydrides were obtained. Organotin(ii) hydrides are an intriguing class of compounds that have first been reported by seminal works of Power *et al.* ^25,26^ Computational predictions for dimers of the parent SnH_2_ indicated various isomers with low isomerisation barriers.^27,28^ Some of the structural motifs predicted computationally have been experimentally observed (**A** and **B**, Chart 1). Decisive for the type of isomer finally obtained (Sn(μ-H)_2_Sn-bridged *vs.* stannylstannylene SnSnH_2_) is the substitution pattern of the terphenyl moiety applied whereas different isomers may be observed in solid-state and in solution (Chart 1).^29–32^ With reduced bulk of the terphenyl moiety, higher aggregates such as tetramers are observed.^33^ Tin(ii) hydrides **D** monomeric in solid state were described by Jones *et al.* who also reported their catalytic applicability in hydroboration reactions.^34–36^ Examples for donor-stabilized β-diketiminate tin(ii) hydrides **E** and pincer-type complexes **F** and their reactivity in hydro-elementation reactions were reported.^37–41^ Parent [SnH_2_] was shown to be successfully stabilized in a donor–acceptor bisadduct complex (**G**, Chart 1).^42–44^

We realized that the dehydrogenation of organotin hydrides induced by simple amine bases has only been applied for the generation of higher aggregate stannanes but apart from phen_2_SnH_2_ (phen = phenanthryl) we are not aware of any systematic study on bulky substituted dihydrides.^11,45^ Moreover, to the best of our knowledge, reactions of organotin trihydrides and amine bases have not been investigated previously in the literature. We now consider organotin trihydrides with substituents of sufficient steric bulk to provide kinetic stabilization to yield the respective organotin(ii) hydride upon hydrogen release catalysed by an amine base. The conditions under which these hydrides are synthetically accessed are so far limited to metathesis of ArSn(ii) halides, hydroboration of ArSn(ii) amides, Sn–C hydrogenolysis of bulky stannylenes, and importantly, hydrogenation of distannynes ArSnSnAr (Ar = various bulky terphenyl moieties).^25,29,30,46^ The reverse reaction to form donor stabilized distannynes *via* dehydrogenation of unstable organotin(ii) hydride intermediates is also known.^47^

From a general synthetic perspective we consider a synthetic protocol toward organotin(ii) hydrides that does not rely on low-oxidation state precursor materials to represent a valuable addition for the synthesis of these species. Furthermore reductive eliminations in general are critical steps in many transition metal catalysed transformations but are less common for main group elements.^48^ The phenomenon of a readily occurring reductive elimination to produce low-valent species in the main groups represents a reaction of general interest to the chemical community in the development and understanding of main group catalysed reactions.^48^

## Results and discussion

### Thermodynamic considerations on the dehydrogenation of organotin trihydrides

In the course of their studies toward the hydrogenation of group 14 dimetallynes Power *et al.* found that in the case of distannynes only one equivalent of H_2_ was consumed in order to yield isomers of [ArSnH]_2_. As already mentioned the bulkiness of the terphenyl ligand was found to be decisive which isomers of dimeric ArSnH were obtained (Sn(μ-H)_2_Sn-bridged *vs.* stannylstannylene SnSnH_2_). This is in accordance with the computationally obtained small energetic differences between the various potential isomers and low-activation energy barriers for their interconversion.^30,49^ Even with large excess of hydrogen no further hydrogen uptake towards distannane ArH_2_Sn–SnH_2_Ar or even ArSnH_3_ was observed, even though the reaction was computed to be thermodynamically favourable.^29,49^ This contrasts the observations for the digermyne reaction under hydrogen atmosphere which yielded mixtures of ArHGeGeHAr, ArH_2_GeGeH_2_Ar and ArGeH_3_.^50^ This deviating reaction behaviour was further discussed in an extensive computational study that provided further insight into the mechanism of the hydrogen uptake reaction and rearrangement/isomerization processes in the hydrogenated species.^49^ For the Ar′-system (Ar′ = 2,6-(2,6-diisopropylphenyl)C_6_H_3_–), the free hydrogenation enthalpy for the hydrogen uptake from monomeric Ar′SnH to Ar′SnH_3_ was computed to be slightly exergonic (Δ*G* = –2.7 kcal mol^–1^). Nevertheless, the energy barrier (Δ*G* ^‡^ = 41.3 kcal mol^–1^) for this reaction and accordingly even more for the reverse dehydrogenation reaction was found to be too high to proceed.^49^

According to our calculated enthalpies in the gas phase (BP86/def2-TZVP) the dehydrogenation of Ar*SnH_3_ (**1**) [Ar* = 2,6-trip_2_(C_6_H_3_)– (trip = 2,4,6-triisopropylphenyl)] to form monomeric Ar*SnH **J** and dihydrogen is found to be endothermic (Δ*H* = 4.6 kcal mol^–1^) but slightly exergonic (Δ*G* = –2.6 kcal mol^–1^). When the experimentally observed dimerization of the monomeric Ar*SnH **J** is taken into account, the overall reaction becomes thermodynamically favourable (Δ*H* = –21.5 kcal mol^–1^, Δ*G* = –19.5 kcal mol^–1^). These preliminary energetic approximations further supported our interest to study possible dehydrogenations of Ar*SnH_3_.

### Improved synthesis of Ar*SnCl_3_

For our studies we chose Ar*SnH_3_ (**1**) with the bulky Ar* terphenyl-moiety since it has impressively been shown to stabilize the seminal [Ar*SnH]_2_ (**H**) accessed by different approaches and thus ensures the stability of potential products. Ar*SnH_3_ (**1**) is conveniently obtained by metathesis of the organotin trichloride Ar*SnCl_3_ (**2**) and LiAlH_4_ in ethereal solutions. However, attempts toward synthesis of **2** from Ar*Li and SnCl_4_ were found to yield mixtures including several hardly separable side-products (Ar*Cl and Ar*H). Oxidative approaches including the chlorination of the conveniently accessible and readily crystallizing Ar*SnCl **3** with some chlorination agents such as PCl_3_ or CCl_4_ were reported in the literature, yet reveal unsatisfactory yields and need further purification procedures.^51,52^ We found mercury(ii) chloride to be a perfectly suitable oxidizing agent for the chlorination of **3**. Addition of stoichiometric amounts of HgCl_2_ to cooled solutions of Ar*SnCl **3** in toluene result in immediate decolourisation of the solution and most likely formation of transient Ar*SnCl_2_HgCl. On warming to room temperature mercury precipitates and after isolation of the supernatant solution and extraction with hexane Ar*SnCl_3_ **2** can be isolated in quantitative yield in elemental analysis grade purity without further purification.

### Pyridine-base induced dehydrogenation

We studied the reactions of **1** with various equivalents of pyridine bases. Initial experiments involving the application of one equivalent of 4-dimethylaminopyridine (DMAP) in benzene at room temperature revealed a slow change of the solutions from colorless to yellow. The formation of a gas is indicated by occasional observation of bubbles. For solutions of [Ar*SnH]_2_ **H** a blue color was described by Power.^25,30^ ^1^H- as well as ^119^Sn-NMR spectroscopic surveillance of the reaction progress revealed ongoing consumption of the starting material Ar*SnH_3_ **1** along with formation of a mixture of compounds, among which dihydrogen can be clearly identified by its characteristic signal at *δ* = 4.47 ppm in the ^1^H NMR spectra. When kept at room temperature the reaction was finished within ten days. ^1^H-coupled ^119^Sn NMR spectroscopy of the intense yellow solution revealed two sets of signals, yet not the signals expected for [Ar*SnH]_2_ **H**. A doublet was found at *δ* = 225 ppm with a rather small coupling constant of *J*_SnH_ = 116 Hz and a spectrum of higher order (see ESI†) including a triplet of triplets pattern at *δ* = –394 ppm with coupling constants of *J*_SnH_ = 1780 Hz and 148 Hz. Sn–Sn coupling was observed, though poorly resolved. The latter coupling pattern can only be assigned to the Sn–Sn dehydrocoupling product diorganodistannane Ar*(H)_2_Sn–Sn(H)_2_Ar* (**4**). The dimensions of the observed coupling constants agree well with ^1^ *J*_SnH_ and ^2^ *J*_SnH_ coupling constants known in the literature.^3^

The second signal at lower field can be assigned to Ar*SnH(DMAP) **5** which represents the DMAP base-adduct to the Lewis-acidic tin(ii) moiety of the monomer of **H**, Ar*SnH **J**. The ^1^ *J*_SnH_ = 116 Hz coupling constant is strongly reduced compared to the coupling observed for the donor-free **H** [Ar*SnH]_2_ of 594 Hz. In solution **H** most likely exists as the stannylstannylene-type isomer Ar*SnSnH_2_Ar* **H_B_** (analogous to **B**, Chart 1), although in solid state the hydride-bridged isomer Ar*Sn(μ-H)_2_SnAr* **H_A_** (analogous to **A**, Chart 1) is observed.^25,30^ The observed coupling constant is in accord with the coupling in related tricoordinate organotin(ii) hydride complexes with terminal Sn–H bond such as found in the carbene adduct Ar*SnH(NHC) (223 Hz)^24^ and diketiminate/pincer complexes **E** and **F** (64/112 Hz).^39,41^

**Scheme 2:**
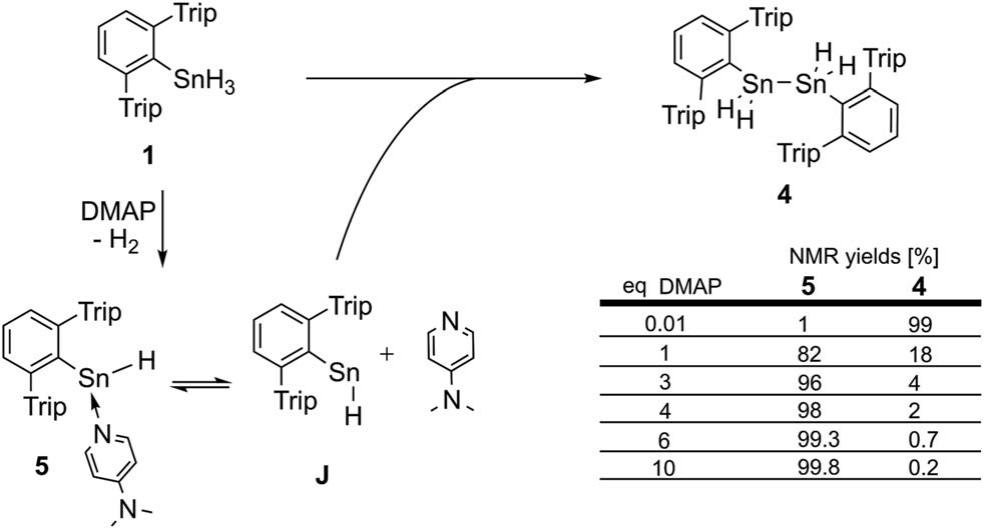
Dehydrogenation of Ar*SnH_3_ **1** with DMAP and summary of the dependency of the product ratios from the relative DMAP concentration.

The observed product mixture can be rationalized by a DMAP induced elimination of dihydrogen from Ar*SnH_3_ **1** under formation of the base-adduct **5** to the monomeric hydrido stannylene Ar*SnH **J**. Diorganodistannane **4** is then most likely formed by insertion of intermediately formed **J** into the Sn–H bond of Ar*SnH_3_ **1**.^53,54^ From the observed generation of distannane **4** we conclude that there has to be an equilibrium between Ar*SnH(DMAP) **5** and uncoordinated Ar*SnH **J** that provides sufficient amounts of **J** for subsequent reactions (Scheme 2). This assumption is further supported by the observation of broad resonances in ^1^H NMR spectra at room temperature indicating dynamic coordination behaviour.^55^

### Controlled dehydrocoupling *vs.* adduct-formation

In order to test our hypotheses we carried out reactions of Ar*SnH_3_ **1** with catalytic amounts (1 mol%) of DMAP base. As expected, distannane **4** is formed quantitatively along with traces of Ar*SnH(DMAP) **5**. At room temperature the reaction proceeded slowly (up to three weeks), but on warming to 65 °C Ar*SnH_3_ **1** was consumed within three days and no significant side products were observed. This is interesting since especially organotin trihydrides are usually thermally sensitive and often decompose under formation of elemental tin.^3,56,57^ Mixtures of distannane **4** and DMAP remained stable and no further elimination of dihydrogen was observed. Along with ligand-related resonances, ^1^H NMR spectra of **4** revealed the Sn–H protons at *δ* = –3.93 ppm accompanied by the satellites reflecting the complex mixture of ^117/119^Sn isotopomers (see ESI†). Large colorless crystals of **4** could be reproducibly obtained from saturated solutions in benzene. However, the crystals diffracted poorly with large cell dimensions, therefore no adequate solution could be found and only the suggested Ar*Sn–SnAr* moiety could be confirmed, since resolution of the hydride atoms was impossible (see ESI†).

Accordingly, reactions of Ar*SnH_3_ **1** with increasing excess of DMAP led to selective formation of Ar*SnH(DMAP) **5** and successful suppression of the subsequent reaction that yields distannane **4**. Whilst three equivalents of base allowed formation of 4 mol% of **4** as a side product only about 0.2 mol% were formed on application of ten equivalents of DMAP (Scheme 2).

The ^1^H NMR spectrum of reaction mixtures including Ar*SnH(DMAP) **5** and excessive DMAP revealed a broad singlet at *δ* = 11.6 ppm accompanied by tin satellites with an integration ratio corresponding to the natural abundance of NMR active tin isotopes strongly indicating the monomeric nature of the species. The signal is assigned to the Sn–H proton and is low-field shifted compared to the NMR spectroscopic features of [Ar*SnH]_2_ **H**. The shift compares well with Roesky's three coordinate Sn–H complexes **E**.^41^ The DMAP adduct to a bulky amido tin hydride was reported by Jones.^34^

The mixtures of **5** and DMAP in solution revealed remarkable thermal stability. Even upon prolonged heating to 85 °C no decomposition was observed. For [Ar*SnH]_2_ **H** and related terphenyl tin(ii) hydride [Ar′SnH]_2_ decomposition with dehydrogenation and ligand stripping under formation of metal-rich clusters is known in refluxing toluene.^32,58^ The carbene-adduct of the much less bulky substituted tripSnH(NHC) **K** (trip = 2,4,6-triisopropyl-phenyl, NHC = 1,3-diethyl-4,5-dimethylimidazol-2-ylidene) generated *in situ* is thermally sensitive and already decomposes at temperatures above –20 °C.^24^ Upon heating, the Sn–H resonance of **5** gradually broadened and shifted toward lower field by 0.2 ppm at 85 °C. The Sn–H coupling constant decreased by approx. 5 Hz each 10 K. This was accompanied by a sharpening of DMAP related signals at elevated temperatures along with subtle shifts of the DMAP related signals. With high excess of DMAP the related signals were found to be much sharper. These findings indicate a dynamic exchange of coordinated and free DMAP. Unfortunately, all attempts to crystallize **5** from these mixtures remained unsuccessful.

**Fig. 1:**
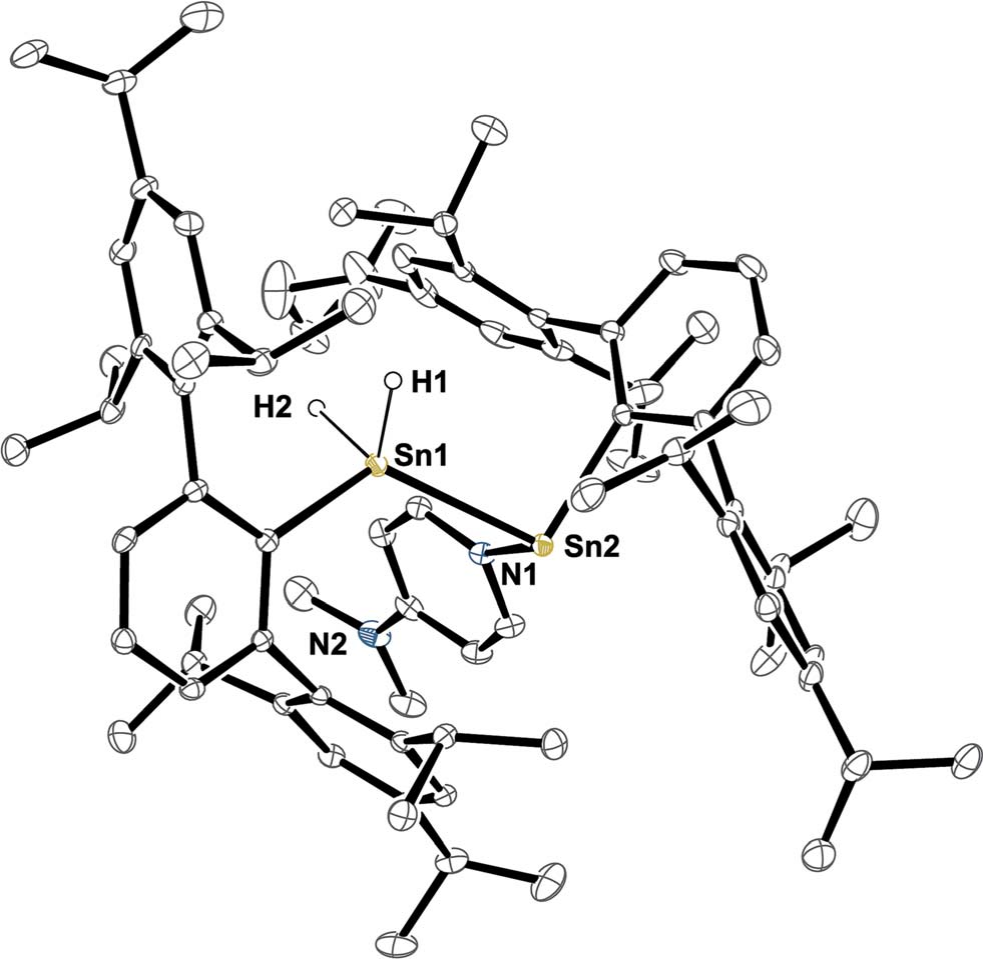
ORTEP plot of the molecular structure of **6**. Thermal ellipsoids are shown at 30% probability level. All hydrogen atoms but H1 and H2 as well as co-crystallized solvent molecules have been omitted for clarity. H1 and H2 were located in the difference Fourier map and their Sn–H contacts are underestimated. Selected bond lengths [Å] and angles are given: Sn1–Sn2 2.8792(3), Sn2–N1 2.316(3), Sn2–C_*ipso*_ 2.271(3), Sn1–C_*ipso*_ 2.190(3), Sn1–H1 1.58(5), Sn1–H2 1.62(5); C_*ipso*_–Sn2–Sn1 96.12(8)°, Sn2–Sn1–C_*ipso*_ 124.65(9)°, Sn1–Sn2–N1 92.77(8)°, C_*ipso*_–Sn2–N1 103.34(11)°.

When the solvents of the reaction mixtures of **5** and DMAP were removed *in vacuo*, yellow solutions were obtained by extraction with pentane along with a residual white solid that corresponds to approx. 80–90% of the excessive DMAP. Storage of these extracts at –40 °C led to further crystallization of colorless DMAP and yellow solutions. When these solutions were again separated from the crystallized solid and subsequently warmed to room temperature they immediately turned pale green. On storing these solutions at –40 °C they turned back to yellow and yellow crystals were obtained within four days. X-ray diffraction did not reveal the expected moiety of Ar*SnH(DMAP) **5** but the DMAP adduct to the stannylstannylene-type isomer **H_B_** Ar*Sn(DMAP)SnH_2_Ar* **6** (Fig. 1).

Structural features of **6** include a Sn–Sn bond of 2.8792(3) Å which is slightly shorter as found for the bulkier substituted uncoordinated stannylstannylenes (2.916/2.924 Å).^29,30^ The Sn–DMAP vector is almost perpendicular to the C_*ipso*_–Sn1–Sn2 plane in accord with an electron donating interaction into the empty p-orbital at the stannylene Sn atom, which becomes a stereochemically active centre. However, NMR spectroscopy indicates a dynamic behaviour in solution (also see ESI†).

**Scheme 3:**
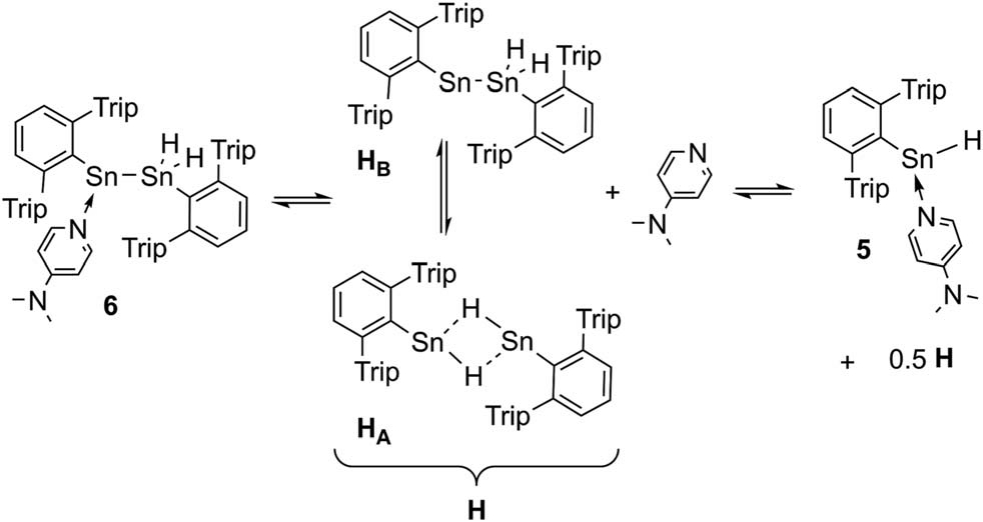
Suggested equilibrium between Ar*Sn(DMAP)SnH_2_Ar* **6** and Power's uncoordinated tin hydride [Ar*SnH]_2_ **H**, as well as base adduct Ar*SnH(DMAP) **5**.

^1^H NMR spectra of green solutions of dissolved crystals of Ar*Sn(DMAP)SnH_2_Ar* **6** at room temperature revealed broad ligand related sets of signals and Sn–H signals likely corresponding to Ar*SnH(DMAP) **5**. Broad DMAP related signal sets allow assumption of a dynamic equilibrium leading to compound mixtures. The same broad ^1^H NMR spectrum was obtained upon addition of 0.5 equivalents of DMAP to solutions of **H** obtained from an alternative approach (*vide infra*). The assumption of an equilibrium is further supported by the observation of four resonances in the ^119^Sn NMR spectrum at *δ* = 698 (**H**, broad), 398 (**6**-SnN), 225 (**5**) and –248 (**6**-SnH_2_) ppm. These represent all compounds of the equilibrium proposed in Scheme 3. On cooling the solutions of **6** to –40 °C well resolved NMR spectra could be obtained with sharp signal sets for all species. A set of signals in the spectra can be assigned to the adduct of a stannylstannylene which indicates the presence of **6** in solution. The tin hydride resonances appeared as two separate singlets at *δ* = 5.60 and 5.51 ppm in accord with the chiral environment at the neighbouring DMAP coordinated stannylene Sn atom. The inequivalency of the hydride signals is also reflected in the coupling pattern of the respective ^119^Sn resonance (**6**-SnH_2_) as a doublet of doublet with coupling constants of ^1^ *J*_Sn–H_ = 1230 and 1040 Hz. The chemical shift of both tin resonances in **6** are in accord with literature values for base-adducts to stannylenes and tin dihydrides.^24,59,60^ IR spectra revealed stretching and bending frequencies of the Sn–H bonds to be at 1763 cm^–1^ and 600 cm^–1^. A donor-adduct to an analogous germylgermylene was previously reported by Power.^61^

When parent pyridine was applied in THF solutions of Ar*SnH_3_ **1** with a vast excess of pyridine (approximately 100 equivalents) the reaction proceeded much slower than with DMAP. After one day at room temperature only 30% of Ar*SnH_3_ **1** was consumed. However NMR spectroscopic examinations indicate formation of the pyridine adduct Ar*SnH(py) **7** analogous to Ar*SnH(DMAP) **5**. The characteristic Sn–H resonances were found at *δ* = 386 ppm (^119^Sn) and 12.11 ppm (^1^H, ^1^ *J*_Sn–H_ = 100.2 Hz). In neat, carefully dried pyridine Ar*SnH_3_ **1** is less soluble leading to cloudy suspensions and no obvious reaction was observed at room temperature. On heating of the mixtures above 80 °C **1** dissolved and a vivid evolution of gas was observed along with a colour change of the solution to yellow. Although pyridine is used as the solvent and therefore in vast excess, formation of approx. 2–4 mol% of distannane **4** was observed from these mixtures. Compared with the findings for excessive application of DMAP, this indicates that the desired suppression of the distannane formation by forcing the putative equilibrium between Ar*SnH(py) **7** and free Ar*SnH **J** with high excess of pyridine towards the expectably less reactive adduct species **7** is overcompensated by the expectably less stable stannylene adduct of pyridine *vs.* DMAP. DFT calculations (BP86/def2TZVP) were run to approximate the free bond dissociation energies (BDE) for cleavage of base adducts to monomeric Ar*SnH **J**.^62^ The computed BDE reflect the weaker adducts formed with pyridine Ar*SnH(py) **7** (BDE = 8.7 kcal mol^–1^) compared to Ar*SnH(DMAP) (BDE = 11.3 kcal mol^–1^). The lower dissociation energy is also reflected in the observation of a subtle but obvious color change from yellow toward a pale green when the pyridine solutions were heated to approx. 90 °C in an NMR tube, putatively indicating the presence of a small amount of the blue compound [Ar*SnH]_2_ **H**. This was not observed for solutions of Ar*SnH(DMAP) **5** with excessive DMAP. The NMR spectra of the reaction mixture in pyridine reproducibly revealed the formation of a further, yet not assigned side product with a ^119^Sn resonance close to the Sn–H signal (*δ* = 383 ppm). This side product is most likely an isomer or solvate complex in equilibrium with Ar*SnH(py) **7** since it was completely consumed in trapping experiments with tetramethyl fulvene (*vide infra*) to yield the identical product. The obviously higher activation energy for the dehydrogenation using pyridine may be rationalized by either the lower nucleophilicity or lower basicity of pyridine (*vide infra*).

### Amine-base induced dehydrogenation

In order to further expand the synthetic value of this dehydrogenative approach as a more general route toward organotin(ii) hydrides it was highly desirable to find conditions under which the uncoordinated [Ar*SnH]_2_ **H** could be generated. DFT computations indicated that an even lower BDE could be expected for adducts of trialkylamine bases such as NMe_3_ (5.1 kcal mol^–1^). The lower BDE may result from both electronic as well as steric effects from interaction with the bulky terphenyl moiety Ar*. Therefore we tested the applicability of various alkyl amines. When a twenty fold excesses the sterically demanding Hünigs base i-Pr_2_NEt, which is generally considered a strong non-nucleophilic base, was tested a slow color change to a deep blue was observed. Ar*SnH_3_ **1** was consumed quantitatively after 36 h and ^1^H NMR spectroscopy revealed the formation of a mixture of three compounds including the signal sets of stannylstannylene type isomer [Ar*SnH]_2_ **H_B_** (approx. 50 mol%), distannane **4** (35 mol%) and an unknown compound (15 mol%). This unknown compound featured a Sn–H resonance at *δ* = 9.68 ppm and revealed broad tin satellites with *J*_Sn–H_ = 91.3 Hz. At first we expected the resonance to correspond to a base adduct Ar*SnH(Ni–Pr_2_Et). However, the integration ratio of the signals ^119^Sn satellites with respect to the NMR inactive isotopomer resonance reflected twice the natural abundance of NMR active tin nuclei. This strongly indicates the presence of a hydride species coupling with two magnetically equivalent tin atoms, as it is realized in the μ-H-bridged isomer Ar*Sn(μ-H)_2_SnAr* **H_A_**. To the best of our knowledge, solutions of [Ar*SnH]_2_ **H** have so far only been reported to reveal the signal sets for the stannylstannylene isomer **H_B_**. Observation of the μ-H-bridged isomer Ar*Sn(μ-H)_2_SnAr* **H_A_** may be attributed to the different polarity of the solvent mixtures including the amine, or a further isomerization pathway that is opened by the amine base present allowing an equilibrium in solution. Along with the small coupling constant the assignment of the resonance at *δ* = 9.68 ppm to the hydrogen bridged isomer to Ar*Sn(μ-H)_2_SnAr* **H_A_** is in accord with Power's observations in extended studies on the structural peculiarities of various terphenyl tin(ii) hydrides.^29,30^ The assumption is further supported by chemical derivatisation experiments (*vide infra*).

The observed formation of larger amounts of distannane **4** may be explained by the sterically well shielded amine. Therefore the dehydrogenation step may be less favourable with a consequently slower consumption of Ar*SnH_3_ **1**. Moreover less effective coordination and therefore less effective deactivation of *in situ* generated Lewis-acidic monomeric Ar*SnH **J** may cause the insertion reaction to be promoted. Consequently, less bulky substituted amines such as TMEDA (*N*,*N*,*N*′,*N*′-tetramethylethylene-diamine) or Et_2_NMe were tested. After a few seconds the mixtures revealed a pale yellow colour (for Et_2_NMe) that quickly turned deep blue after a few minutes. The reactions were finished within a few hours. NMR spectroscopy revealed these solutions to almost exclusively contain **H_B_** and **H_A_** (approx. 95%) in a molar ratio of approximately 4 : 1. The molar ratio of **H_B_** to **H_A_** was found to be independent of the amount of amine base present in solution. We assign the initially observed yellow colour to Ar*SnH(NR_3_) adducts which are present in dilute solutions of newly generated Ar*SnH **J** and excessive amine. Due to the weak donor-interaction of the amine we expect that upon increasing concentrations of Ar*SnH(NR_3_) in the course of the dehydrogenation, deep blue dimeric **H** is formed. Distannane **4** was formed to a negligible degree (TMEDA: <1 mol%; Et_2_NMe: <1 mol%). At room temperature, the solutions remained stable and no decomposition was observed for days. Nevertheless, when volatiles including the amines were removed *in vacuo* at room temperature, examinations of the pale green to yellowish residue revealed further side-products (mainly Ar*SnH which may stem from ligand stripping).^58^ This is surprising since **H** was found to be isolable and stable in the solid state.^25^ This limits the preparative value of the approach. From a practical perspective, Et_2_NMe was used preferably since it is much easier removed *in vacuo*.

### Sn–H trapping reactions with tetramethyl fulvene

**Scheme 4:**
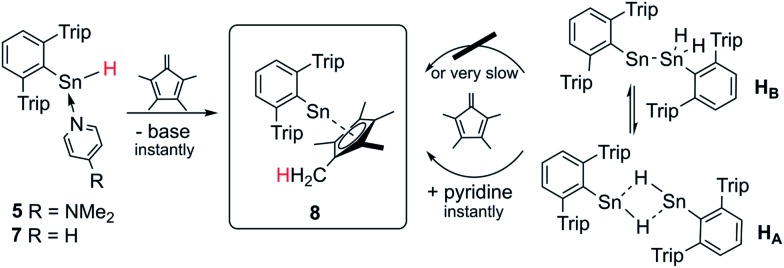
Schematic synthesis of Ar*SnCp* **8** by hydrostannylenylation of tetramethyl fulvene from *in situ* generated solutions of Ar*SnH.

**Fig. 2:**
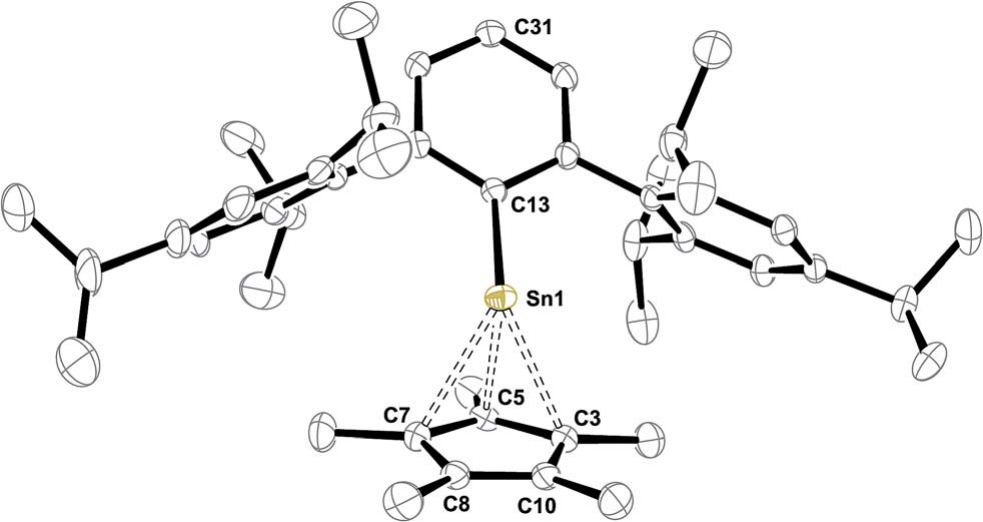
ORTEP plot of the molecular structure of Ar*SnCp* **8**. Thermal ellipsoids are shown at 50% probability level. All hydrogen atoms as well as co-crystallized solvent molecule have been omitted for clarity. Selected bond lengths [Å] and angles [°] are given: Sn1–C13 2.2678(19), Sn1–C5 2.398(2), Sn1–C3 2.543(2), Sn1–C7 2.559(2), Sn1–C8 2.859, Sn1–C10 2.845, C3–C5 1.434(3), C5–C7 1.432(3), C7–C8 1.426(3), C8–C10 1.386(3), C10–C3 1.430(3), C31–C13–Sn1 162.1°.

In order to test the reactivity and preparative applicability of the crude solutions of Ar*SnH(DMAP) **5**, Ar*SnH(py) **7** and the isomer mixtures of [Ar*SnH]_2_ **H_A_**/**H_B_** obtained by this dehydrogenation approach, trapping experiments of the Sn(ii)–H moiety have been conducted using 1,2,3,4-tetramethyl fulvene. On addition of stoichiometric amounts of fulvene to monomeric adducts Ar*SnH(DMAP) **5** and Ar*SnH(py) **7** an immediate addition of the Sn(ii)–H bond to the polar fulvenic C=C double bond was observed quantitatively yielding the bright yellow half-sandwich complex Ar*SnCp* **8** in a hydrostannylenylation reaction (Scheme 4). Addition of brightly orange solutions of fulvene to deep blue solutions of *in situ* generated **H_A_**/**H_B_** with amines did not result in a quick reaction and only green mixtures were observed, indicating the reactivity in terms of hydrostannylenylation of C=C double bonds is strongly reduced by the dimeric nature of [Ar*SnH]_2_ **H**. When small amounts of pyridine (few drops) or DMAP (>1 eq.) are added to the green mixtures, bright yellow Ar*SnCp* **8** forms instantly. We suggest that the donor addition forces monomerization and therefore conversion into a more reactive species. After removal of all volatiles Ar*SnCp* **8** could be obtained as a bright yellow solid and crystals suitable for X-ray diffraction could be obtained from concentrated solutions in pentane at –40 °C (Fig. 2).

Structural characterization of Ar*SnCp* **8** reveals the half-sandwich complex of tin(ii) and its strong structural relation to the known silicon homologue Ar*SiCp*.^63^ In solid state, the Cp* moiety reveals closer contacts to three carbon atoms in a η^3^-fashion. The tin atom deviates from the aromatic plane of the central phenyl group by almost 18° which may be in line with an unusually pronounced low-field shift of the *ipso*-C atom resonance in the ^13^C NMR spectrum at *δ* = 195.7 ppm. This arrangement maybe due to a pronounced polarization of the Sn–C single bond with large ionic character. In case of core Cp*–carbon atoms with strong Sn–C interactions, the methyl groups attached lay below the Cp-ring plane whilst the other methyl moieties are slightly twisted into the other direction. Compared to decacoordinated Cp*_2_Sn, the inhomogeneity in Sn–C contacts is increased. The shortest Sn–C_5_ contact in Ar*SnCp* **8** is almost 0.2 Å shorter than in Cp*_2_Sn, whilst the longest distances are almost 0.1 Å longer.^64^ The C_5_ C–C bond lengths are as described for Ar*SiCp* and reveal one short C–C contact. Power reported on Cp derivatives of ArECp (Ar = 2,6-diisopropylphenylphenyl; E = Ge, Sn) from reactions of the respective dimetallynes with CpH.^65^ The related η^3^-coordinated allyl complex Ar*Sn–allyl was reported only recently.^66^ In solution the Cp* moiety reveals only one resonance in ^1^H NMR spectroscopy, indicating dynamic exchange on the NMR time scale. ^119^Sn NMR resonance is found at *δ* = –25 ppm which is well in accord with the literature for a half-sandwich complex.^65^

### Kinetics

**Fig. 3:**
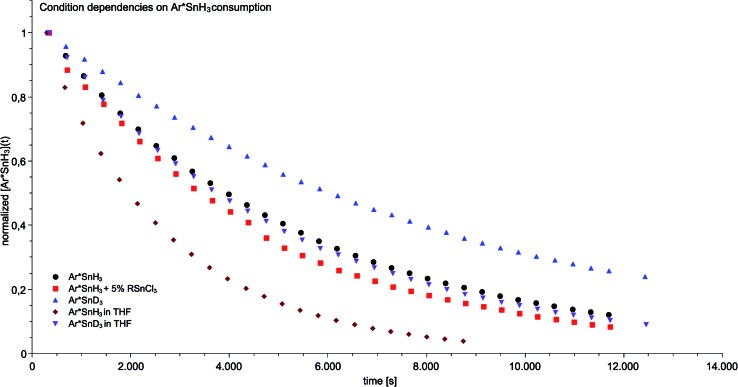
Condition-dependent consumption of tin trihydride/trideuteride (0.046 mol L^–1^) in the course of the catalytic dehydrogenation in the presence of a six fold excess of DMAP at 40 °C determined by ^1^H NMR spectroscopy.

In order to further study the mechanism underlying the observed amine-induced reductive elimination of dihydrogen from Ar*SnH_3_ **1** several experiments were conducted. As a model system for studies on mechanistic aspects of the reaction we choose the reaction of Ar*SnH_3_ **1** with six equivalents of the weighable DMAP in *d*_6_-benzene. The experiments were performed with Ar*SnH_3_ **1** obtained from various crops and different synthetic routes in order to minimize possible systematic errors derived from undetected impurities that are potentially involved in the process. Ar*SnH_3_ **1** cannot be purified by distillation, therefore halide contamination with hardly detectable amounts of potential catalytically active impurities cannot be rigorously excluded. Therefore to test earlier suggestions of a halide promoted process (*vide infra*) traces (up to 5%) of organotin trichlorides such as (trip)SnCl_3_ were added to the reaction mixtures. Hydride/chloride exchange reactions between organotin chlorides and hydrides to form mixed hydrochloro stannanes are known in the literature.^67^ However, apart from organotin halide derived side products (such as trip–H) and a tin metal mirror, we did not observe any significant acceleration of the reaction compared to the model system (Fig. 3). Though it cannot be generally ruled out, we do not consider the proposed involvement of intermediately formed mixed tin halide hydride species to be of relevance for the hydrogen elimination reaction presented (*vide infra*).

In order to gain insight into the mechanism that enables the observed amine-induced reductive elimination of dihydrogen from tin trihydride Ar*SnH_3_ **1** several experiments were conducted. To suppress the side reaction forming distannane **4**, pseudo-first order conditions with excess of DMAP were chosen. From these experiments by observation of the time-dependent consumption of Ar*SnH_3_ **1** *via* ^1^H NMR spectroscopy we conclude that the reaction proceeded *via* first order in the DMAP concentration, since the determined rate constants *k*′ were found to be directly proportional to the concentration of DMAP. Furthermore from an Arrhenius analysis by temperature-dependent measurements of the reaction rates the activation energy of the dehydrogenation in *d*_6_-benzene was determined to be 13.7 ± 1.0 kcal mol^–1^.^68^ For this model reaction at 313 K a kinetic isotope effect *k*′_H_/*k*′_D_ (KIE) was found to be around KIE ≈ 1.65 in *d*_6_-benzene and KIE ≈ 2.04 in *d*_8_-THF.^68^ The maximum KIE for Sn–H/D cleavage was approximated from the stretching frequencies of Ar*SnH_3_ and Ar*SnD_3_ to be KIE_max_ ≈ 3.4.^69^ In the more polar solvent THF the reaction is almost twice as fast with *k*′_thf_/*k*′_benzene_ ≈ 2.0 ± 0.1 (Fig. 3). The preference of a polar solvent is an indication for a polar transition state.

### Amine-base screening

In order to further evaluate the decisive feature (nucleophilicity *vs.* basicity) of the amine bases that enables the reaction to proceed, a series of amine bases were tested on their reactivity with Ar*SnH_3_ **1** (Table 1). In a control experiment Ar*SnH_3_ **1** was heated in toluene at 110 °C for 24 hours and only formation of approximately 1 mol% of distannane **4** was observed. Among pyridine bases, the sterically hindered lutidine (2,6-dimethylpyridine) was found to be only active at elevated temperatures. In a clean reaction (also with higher excesses of lutidine) only formation of distannane **4** was observed. When an exceptionally strong and almost non-nucleophilic amine base such as proton sponge (1,8-dimethylaminonaphthalene) was used, a very slow reaction was only observed under harsh conditions forming exclusively distannane **4**. This reaction behaviour may also result from trace impurities such as other amine bases contained in the proton sponge sample with proton sponge itself being completely incapable to act as dehydrogenation catalyst. As already described, for tertiary alkyl amines the uncoordinated dimeric organotin(ii) hydride **H** was obtained, with the sterically demanding i-Pr_2_NEt yielding significant amounts of product **4**. When the heavier homologue of TMEDA 1,2-bis(dimethylphosphino)ethane (dmpe) was used, no dehydrogenation reaction was observed even at elevated temperatures. However, when TMEDA was added to the mixture of Ar*SnH_3_ **1**/dmpe, dihydrogen evolved followed by putative formation of the phosphine adduct Ar*SnH(dmpe). Dynamic coordination behaviour in solution is indicated by a broad ^31^P NMR resonance at *δ* = –48 ppm and a significant change in the ^1^H NMR spectrum revealing the Sn–H resonance at *δ* = 7.80 ppm with coupling constants of *J*_Sn–H_ = 150 Hz. Since in the presence of dmpe dimerization of Ar*SnH to form **H** is energetically disfavoured compared to formation of the adduct Ar*SnH(dmpe), the phosphine donor toward Sn(ii) must be sufficiently stronger than TMEDA.

**Table 1:** Overview on the reactivities and product mixtures obtained from ^1^H NMR screening reactions of Ar*SnH_3_ ^*a*^

	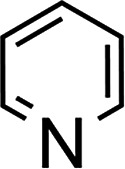	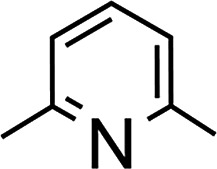	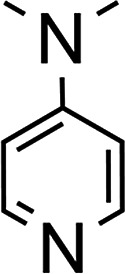	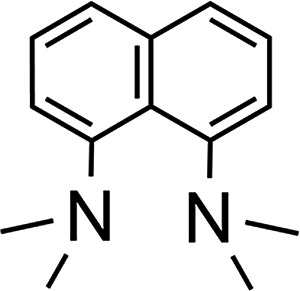	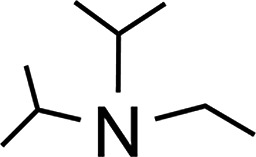	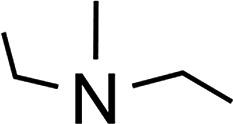	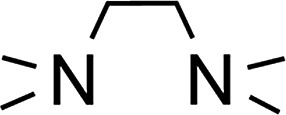	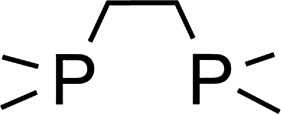
Eq.	>100	10	6	3	20	20	20	3
Conditions	r.t. very slow 90 °C 4 h	r.t. no rxn 100 °C 14 h	r.t. 12 h 50 °C 5 h	65 °C no rxn >100 °C slow^*b*^	r.t. 24 h	r.t. 5 h	r.t. 6 h	r.t. no rxn 90 °C no rxn
[Ar*H_2_Sn]_2_ **4**	4%	99%	<1%	99%^*b*^	35%	1%	1%	0%
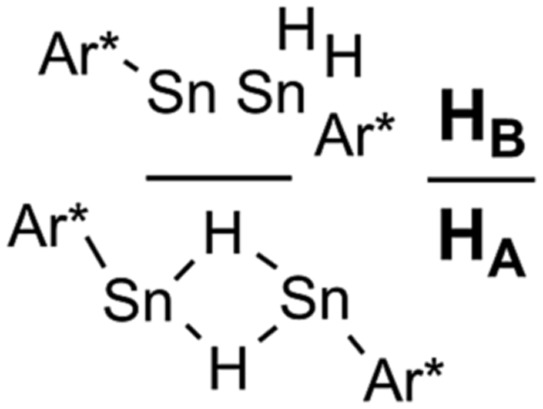	0%	0%	0%	0%	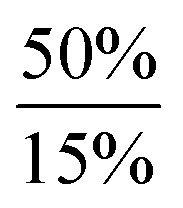	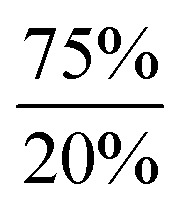 ^*c*^	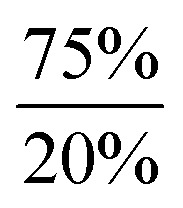 ^*c*^	0%
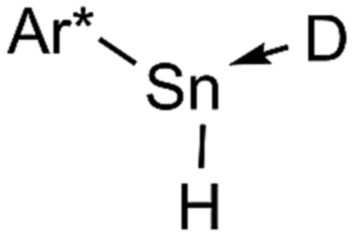	>90%	0%	99%	0%	0%	0%	0%	0%

^*a*^Generally samples containing 25 mg Ar*SnH_3_ in 0.5 mL *d*_6_-benzene were prepared.

^*b*^The reaction was kept at 105 °C for 24 h until only approx. 20% of **1** was consumed.

^*c*^The relative share of **H_A_** to **H_B_** in the resulting mixtures was found to be independent from the amount of amine used. All yields represent NMR yields.

From these observations a few conclusions and comparisons can be drawn:

(1) Larger amounts of distannane **4** are observed if a dative interaction of an initially formed Ar*SnH **J** is disfavoured by steric bulk around the potential donor (lutidine, proton-sponge, iPr_2_NEt).

(2) Phosphine donor (dmpe) does not initiate dehydrogenation, although it is expected to be a potent donor towards Sn.

(3) One of the expectably strongest amine bases tested (proton sponge) reveals only poor activity.^70^

(4) Increasing nucleophilicity and basicity leads to a lowering of the activation energy barrier (pyridine *vs.* DMAP).

For bases in a comparable p*K*_a_ range (TMEDA, Et_2_NMe, DMAP, iPr_2_NEt) efficient dehydrogenation is observed.^70^

### Mechanistic considerations

To the best of our knowledge, apart from suggestions and general observations, literature does not provide conclusive evidence of the chemical processes for the dihydrogen release from R_2_SnH_2_ (R = alkyl or aryl).^5,6,10,14^ Moreover, existing studies on amine-catalysed dehydrogenation of organotin dihydrides R_2_SnH_2_ (R = various alkyl or aryl) go along with the formation of Sn–Sn bonds to yield catenated oligomers or polymers. On account of extensive studies Neumann *et al.* suggested that the essential dehydrogenation processes are intermolecular and take place at the ends of growing chains H–(R_2_Sn)_*n*_–H without the involvement of intermediately generated free diorgano stannylenes R_2_Sn that condense to polymeric chains.^10,12^ Moreover they observed the dehydrogenation to be unaffected by addition of quinones and therefore excluded a radical chain mechanism in favour of a polar one.^5^

In some of their studies on dehydrogenations of R_2_SnH_2_ (R = aryl, alkyl) Neumann *et al.* reported an increased reaction rate when traces of tin halides (such as Ph_2_SnCl_2_) were added as cocatalyst (*vide supra*).^10^ Especially amine-base catalysed dehydrogenation of some organotin dihydrides purified by distillation was observed to be much slower or incomplete compared to crops of organotin dihydride that have not been purified by distillation.^5^ It was suggested that some tin halide impurities may stay in a pre-equilibrium with hydride–halide exchange that result in formation of R_2_Sn(H)Cl from which hydrogen is known to be readily eliminated in the presence of bases forming dichloro distannanes.^10,67^ However, the respective reaction rate was reported to be unaffected by the addition of pyridinium chloride, what somehow contradicts the assumption of the halide supported catalysis.^14^ For the formation of (Bu_2_ClSn)_2_ from Bu_2_Sn(H)Cl Davies *et al.* expected a radical chain mechanism.^67^

Our own observations for dehydrogenation of Ar*SnH_3_ **1** do not indicate a pronounced acceleration due to tin halides. Moreover unlike all previous studies we observed a base-stabilized monomeric stannylene species as a final product. We assume the formation of distannane **4** to result from a subsequent reaction of the monomeric stannylene rather than a separate mechanism for the Sn–Sn bond formation.

**Scheme 5:**
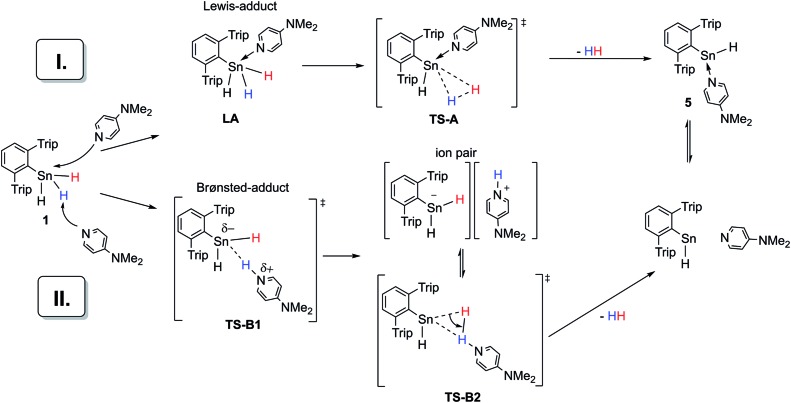
Two general suggestions (I and II) for an amine base driven dehydrogenation of Ar*SnH_3_ **1**.

Taking these facts into account and considering our own observations, two mechanistic pathways (I and II) for the dehydrogenation of Ar*SnH_3_ **1** catalyzed by amine bases seem plausible (Scheme 5). Both rely on the polarizability of the Sn–H bond.^71^ Mechanism I assumes a nucleophilic attack of the amine base at the tin centre forming a Lewis adduct. In this context a recent geometrically enforced donor-facilitated dehydrogenation of an arsine to form an arsinylidene phosphorane needs to be mentioned.^72^ Adducts of a broad variety of (also intramolecular) bases with penta-coordinated tin are well known and crystallographically documented for organotin halides R_4–*n*_SnX_*n*_ (with *n* ≥ 1).^73–77^ However in the case of tetraorgano stannanes without electronegative substituents, indications for donor–acceptor interactions of amines with poorly Lewis acidic Sn is only found in constrained intramolecular arrangements partially leading to increased reactivities of the hydride.^78–82^ The poor Lewis acidity may also be seen from the optical stability of chiral stannanes and tin hydrides even in the presence of bases.^83,84^ On addition of some Lewis-bases triorganotin hydrides have been found to be more active hydride donors.^82,85^ Apart from these reactivity related observations, there is no spectroscopic evidence for self-aggregation or hyper-coordination of organotin hydrides in solution and solid state. However to the best of our knowledge no systematic studies have been made for organotin trihydrides.^3^

In accord with the reluctance of organohydro stannanes toward aggregation and penta-coordination, in our case, NMR spectroscopy does not provide any evidence for a species such as the Lewis-adduct Ar*SnH_3_(DMAP) **LA** (path I, Scheme 5). However, in accompanying DFT calculations at the (TPSS/6-31G*(C,H,N)-def2TZVP(Sn))^86^ level on a model system (PhSnH_3_/DMAP) a Lewis-adduct such as PhSnH_3_(DMAP) is found to be a minimum on the potential energy surface with the formation being exothermic (Δ*H*_LA_ = –11 kcal mol^–1^), but slightly endergonic Δ*G*_LA_ = +4.7 kcal mol^–1^. The N-donor atom is directed towards the penta-coordinated Sn centre. Computations also provided a transition state (**TS-A**) for the extrusion of dihydrogen from the adduct complex (Scheme 5). However, Δ*G*‡TS-A was found to be around 46 kcal mol^–1^. This is too high for a reaction that readily occurs at room temperature and is in disagreement with the experimentally determined activation energy of 13.7 kcal mol^–1^ (*vide supra*). Similar values for Δ*G*_LA_ and Δ*G*‡TS-A have been computed for the corresponding intermediates and transition states when pyridine or Me_3_N or solvent effects applying a polarized continuum model (PCM) for benzene or THF were considered. The computed activation energy found for the dehydrogenation of Lewis adduct PhSnH_3_(DMAP) is comparable to the barrier height computed for the dehydrogenation of an uncoordinated terphenyltin trihydride Ar′SnH_3_.^49^

Mechanistic proposal II is a heterolytic cleavage of a proton from Ar*SnH_3_ **1** by an amine base. Proton abstraction from organotin dihydrides is known for strong bases (LDA, lithium diisopropylamide).^87^ The resulting [R_2_SnH]^–^ anion can serve as a source for nucleophilic tin. Formation of polystannanes was however not observed in these mixtures.^87^ Along with earlier suggestions that the hydrogen in organotin dihydrides reveals more electrophilic character than in organotin monohydrides, further emphasis on the electrophilic nature may be assumed in organotin trihydrides and therefore accessibility for deprotonation under milder conditions seems plausible.^9^ The proposed mechanism proceeds *via* highly polar intermediates, in agreement with the observed acceleration in more polar solvents. Computational insights [TPSS/6-31G*(C,H,N)-def2TZVP(Sn)] into the deprotonation of PhSnH_3_ with NMe_3_ or pyridine in THF (PCM model) revealed a transition state **TS-B1** with Δ*G*‡TS-B1 of 15.8 or 22.3 kcal mol^–1^ which is in reasonable agreement with the experimentally determined value.^88^ Within the narrow scope of valid comparisons of energetic barriers between the experimentally studied system and the chosen model system the computational results allow the consideration of the deprotonation to be involved in the reaction path.^89^ When a rough correction of the entropic term *T*Δ*S*‡TS-B1 for the computational Δ*G*‡TS-B1 was applied,^49,90–92^ the obtained Δ*G*‡TS-B1(corr) reproduce the qualitative trend of the experimentally observed reaction rates.^93^ However such entropy corrections have partly been criticized only recently.^94^

From these agreements, we propose this deprotonation step to be rate determining for the overall reaction. The subsequent protonation under hydride abstraction from the [Ar*SnH_2_]^–^ anion under formation of hydrogen *via* putative **TS-B2** is assumed to be much quicker. In order to access experimental insight whether dihydrogen is directly generated from the proposed ion pair or if the ion pair can separate, a crossover experiment was performed. Indeed, from a 1 : 1 mixture of Ar*SnH_3_ and Ar*SnD_3_ **1-D** with DMAP formation of small amounts of HD gas was observed in ^1^H NMR spectra. However, firm conclusions from this observation are prevented by a slow H/D exchange equilibrium between the educts generating Ar*SnHD_2_ and Ar*SnH_2_D from which HD may also be eliminated intramolecularly. This exchange equilibrium was also confirmed for solutions without addition of DMAP.

From all the experimental and computational indications we propose the successful dehydrogenation of Ar*SnH_3_ **1** to rely on a deprotonation and a subsequent protonation of hydridic hydrogen. Under these premises the base needs to be sufficiently strong for the rate-determining deprotonation step. This is in accord with the observed successful dehydrogenation applying amine bases of similar p*K*_a_ values (NiPr_2_Et, NEt_2_Me, DMAP), whilst weaker bases (pyridine, lutidine) required thermal activation. Less basic though more nucleophilic phosphines do not lead to dehydrogenation. The inefficiency of the strong base proton sponge may be related to the geometrical peculiarities of the basic pocket of proton sponge which may not be suitable for the deprotonation of Ar*SnH_3_ **1**. Apart from this suggested mechanism involvement of contaminations of the educts with hardly detectable side-products was tried to be avoided but can never be generally ruled out. Unlike earlier literature suggestions, in the present case monomeric hydrostannylene Ar*SnH **J** is obviously formed transiently in the course of the reaction. Although without disclosing evidence, we propose the reaction to likely occur at one single Ar*SnH_3_ molecule rather than being intermolecular.

## Conclusions

In conclusion we presented a new approach toward the generation of terphenyltin(ii) hydrides from tetravalent tin(iv) precursors. This salt-free reduction procedure offers novel access to this unique subclass of inorganic and organometallic compounds. A variety of amines and pyridines has been tested on their applicability as potential catalysts for this reduction reaction. It was shown that depending on donor-strength of the applied amine/pyridine base either the unsupported dimers of Ar*SnH **J** were obtained in solution, or the monomeric base-adducts were observed. Without excessive DMAP in solution, Ar*SnH(DMAP) was found to be in an equilibrium with the uncoordinated dimeric hydride [Ar*SnH]_2_ and the crystallographically characterized Ar*Sn(DMAP)SnH_2_Ar* which is the DMAP adduct to its stannylstannylene-type isomer **H_B_**.

Moreover, it was shown that the stoichiometric ratio of DMAP base can determine the product composition. In the case of catalytic amounts of DMAP quantitative dehydrocoupling to form a diorganodistannane Ar*H_2_SnSnH_2_Ar* was observed. The formation is proposed to occur *via* insertion of transiently formed Ar*SnH **J** into the Sn–H bond of Ar*SnH_3_ **1** when efficient stabilization of the reactive Lewis-acidic Sn(ii) center is not provided.

The generated derivatives of Ar*SnH were shown to be easily added to polar fulvenic C=C double bonds in hydrostannylenylation reactions to yield a half-sandwich complex of tin(ii), Ar*SnCp*, which was structurally characterized. In a set of experiments, including deuterated species, the activation energy as well as kinetic isotope effects and solvent dependencies of the dehydrogenation reaction were determined. Along with the results from accompanying computational studies a plausible mechanism of the reaction was proposed to include the deprotonation of the Ar*SnH_3_ as the rate-determining step.

The overall reaction represents a rare example for a catalytic reductive elimination from a tetravalent group 14 element to form a monomeric tin(ii) species. This reaction may be seen as a tin organometallic equivalent to the long known reduction of PbCl_4_ to PbCl_2_ under reductive elimination of chlorine. Reductive eliminations are among the key steps in important catalytic transformations and their observation in main groups is of special interest for further development of main group catalysed transformations.

## Experimental details

### General information

All manipulations were carried out under argon atmosphere using standard Schlenk techniques or an MBraun Glovebox. THF, diethylether and benzene were distilled from sodium/benzophenone, toluene from sodium. Hexane and pentane were obtained from an MBRAUN solvent purification system and degassed. Benzene-*d*_6_ was distilled from sodium and stored over potassium, THF-*d*_8_ was distilled from LiAlD_4_ and stored under exclusion of light at –40 °C, pyridine-*d*_5_ was distilled from calcium hydride and subsequently from sodium. DMAP, proton sponge and lutidine were obtained commercially (99%, Aldrich) and used without further purification, i-Pr_2_NEt, Et_2_NMe and TMEDA were obtained commercially (Aldrich) and distilled from CaH_2_ (i-Pr_2_NEt, Et_2_NMe) or from *n*-BuLi (TMEDA) and degassed.

Terphenyl-iodide (Ar*I),^95^ -lithium-etherate (Ar*Li(OEt_2_)),^95^ -Sn(ii) chloride (Ar*SnCl)^96^ and trihydride (Ar*SnH_3_)^51^ were prepared according to modified literature procedures. Ar*SnD_3_ was described earlier.^97^ 1,2,3,4-Tetramethyl fulvene was prepared according to a literature procedure.^98^

Elemental analysis was performed by the Institut für Anorganische Chemie, Universität Tübingen using a Vario MICRO EL analyzer. IR spectra were recorded as KBr pellets prepared in a glovebox and measured with a Bruker VERTEX 70 IR spectrometer. Detailed information of the conducted kinetic studies are given in the ESI.†

X-ray data for **1**, **6** and **8** were collected with a Bruker Smart APEX II diffractometer with graphite-monochromated Mo Kα radiation. The programs used were Bruker's APEX2 v2011.8-0 including SADABS for absorption correction and SAINT for structure solution, as well as the ShelXLE graphical user interface for ShelXL for structure refinement.^99–101^ For further refinement details see the attached .cif-files and the details provided in the ESI.†

### NMR spectroscopy

NMR spectra were recorded with either a Bruker DRX-250 NMR spectrometer equipped with a 5 mm ATM probe head and operating at 250.13 (^1^H), 62.90 (^13^C) and 93.31 MHz (^119^Sn), a Bruker AvanceII+400 NMR spectrometer equipped with a 5 mm QNP (quad nucleus probe) head and operating at 400.13 (^1^H), 100.62 (^13^C) or a Bruker AVII+500 NMR spectrometer with a variable temperature set up and a 5 mm ATM probe head or a 5 mm TBO probe head and operating at 500.13 (^1^H), 125.76 (^13^C), 186.55 MHz (^119^Sn). Chemical shifts are reported in *δ* values in ppm referenced on the solvent ^2^H resonance frequency C_6_D_6_ (^1^H 7.15 ppm; ^13^C 128.0 ppm), tol-*d*_8_ (^1^H 2.09), py-*d*_5_ (^1^H 8.76, ^13^C 150.2). The proton and carbon signals were assigned where possible *via* a detailed analysis of ^1^H, ^13^C or ^13^C-UDEFT, ^1^H–^1^H COSY, ^1^H–^13^C HSQC, ^1^H–^13^C HMBC NMR spectra. Selected 1D-NMR spectra of the compounds and mixtures can be found in the ESI.†

### Computational details

DFT calculations have been performed using the Gaussian09 Revision D.01 program.^102^ For the calculations of bond dissociation energies, the structures have been optimized using the BP86 functional^103,104^ with def2TZVP basis set on all atoms (C, H, N, Sn) along with w06 density fitting^105,106^ as well as Stuttgart–Dresden effective core potentials on tin as implemented in Gaussian. Grimme dispersion correction with Becke-Johnson damping has been taken into account using the D3BJ option implemented in Gaussian.^107^ Starting geometries for geometry optimizations were directly taken from the X-ray data were available (Ar*SnH_3_ and [Ar*SnH]_2_) or from manipulations on the basis of these X-ray structures (monomeric Ar*SnH). Starting geometries for the optimization of base-adducts to Ar*SnH were obtained from substitution of the donor molecule on the basis of the structure of Ar*SnH(NHC).^24^ Thermal corrections were obtained from frequency calculations performed for all optimized structures. Frequency calculations revealed no or a single imaginary frequency smaller than 9 cm^–1^, except for Ar*SnH were two small imaginary frequencies were obtained (6 and 11 cm^–1^).

For the studies of the mechanism dehydrogenation of model compound PhSnH_3_ was chosen. Computations were carried out in Gaussian09 Revision D.01 using TPSS functional^108^ along with 6-31G* basis set^109^ for C, H and N and a def2TZVP basis set along with Stuttgart–Dresden ECP for Sn and dispersion correction as implemented in Gaussian. Solvent THF was simulated by a PCM model. A table of the computed energies at standard conditions and details on the geometry optimizations is given in the ESI.†

### Synthesis

#### Ar*H_2_Sn–SnH_2_Ar* (**4**)

To a solution of Ar*SnH_3_ (200 mg, 0.331 mmol, 1 eq.) in benzene (2 mL) catalytical amounts of DMAP (0.3 mL, stock solution in benzene: 1.0 mg/1 mL, 2.5 μmol, 0.7 mol%) was added and the solution was stirred at 70 °C for 14 days. The resulting beige solution was filtered through a syringe filter and the solvent was removed under reduced pressure to quantitatively yield Ar*H_2_Sn–SnH_2_Ar* as a colorless powder (195.7 mg, 0.162 mmol, 98%). From saturated solutions of warm benzene large colorless crystals of Ar*H_2_Sn–SnH_2_Ar* can be obtained, however these crystals were not suitable to provide high quality X-ray diffraction data. Analytical data: ^1^H-NMR (C_6_D_6_, 298 K, 400.11 MHz): *δ* = 7.14 (s, 8H, *m*-C*H*_trip_), 7.12–7.09 (m, 6H, *m*-/*p*-C*H*_Ph_), 3.94 (s + satellites, 4H, ^1^ *J*_119Sn–H_ = 1777 Hz, Sn–*H*, ^1^ *J*_117Sn–H_ = 1700 Hz, ^2^ *J*_117/119Sn–H_ = 145 Hz, Sn–*H*), 2.91 (sept., 4H, ^3^ *J*_H–H_ = 6.93 Hz, *p*-C*H*Me_2_), 2.76 (sept., 8H, ^3^ *J*_H–H_ = 6.91 Hz, *o*-C*H*Me_2_), 1.36 (d, 24H, ^3^ *J*_H–H_ = 6.96 Hz, *p*-CH*Me*_2_), 1.28 (d, 24H, ^3^ *J*_H–H_ = 6.90 Hz, *o*-CH*Me*_2_), 1.06 (d, 24H, ^3^ *J*_H–H_ = 6.86 Hz, *o*-CH*Me*_2_); ^13^C{^1^H} (C_6_D_6_, 298 K, 100.62 MHz): *δ* = 149.2 (*ipso-C*_*Ph*_), 148.5 (*p-C*_*trip*_), 146.3 (*o-C*_*trip*_), 141.8 (*o-C*_*Ph*_), 140.3 (*ipso-C*_*trip*_), 128.9 (s, *m-C*_*Ph*_), 127.4 (s, *p-C*_*Ph*_), 121.3(*m-C*_*trip*_), 34.8 (s, *p-C*HMe_2_), 30.9 (s, *o-C*HMe_2_), 26.0 (s, *o*-CH*Me*_2_), 24.5 (s, *p*-CH*Me*_2_), 23.6 (*o*-CH*Me*_2_); ^119^Sn–^1^H-coupled (C_6_D_6_, 298 K, 93.24 MHz) *δ* = –393.4 (tt, ^1^ *J*_119Sn–H_ = 1777 Hz, ^2^ *J*_119Sn–H_ = 146 Hz). Anal. calcd for [**4**] C_72_H_102_Sn_2_: C 71.77, H 8.53; found: C 71.44, H 8.39. IR (KBr, cm^–1^): 1875 (s, Sn–H, stretch), 560 (s, Sn–H, bend).

#### Ar*SnH(DMAP) (**5**)

##### Method A

To a solution of Ar*SnH_3_ (200 mg, 0.331 mmol, 1 eq.) in benzene (3 mL) a solution of 4-*N*,*N*-dimethylaminopyridine (DMAP) (242.9 mg, 1.988 mmol, 6 eq.) in benzene (3 mL) was added at room temperature. Hydrogen evolves and the increasingly yellow mixture was stirred overnight until the reaction was complete. Solvents are removed under reduced pressure and to quantitatively yield [Ar*SnH × 6(DMAP)] as a yellow powder, that can be used for any further reactions. Excessive DMAP can be removed by extraction of the residue with minimum amounts of pentane (approx. 2 × 1.5 mL) to yield [Ar*SnH × 1–1.5(DMAP)]. In solution, DMAP coordination reveals to be dynamic, therefore sharp NMR spectra of [Ar*SnH × 1–1.5(DMAP)] cannot be obtained at room temperature. From solutions with excessive DMAP (6–10 eq.) sharp ^1^H NMR spectra with the signals sets for Ar*SnH(DMAP) can be obtained. The NMR spectra reported reflects the signals sets with 10 eq. DMAP.

##### Method B

A solution of **H_A_**/**H_B_** was prepared as described (*vide infra*) from Ar*SnH_3_ and Et_2_NMe (approx. 10 eq.) and the deep blue mixture was kept for 6 hours before DMAP (1 eq.) was added to yield a yellow solution. The solution is stirred for 15 min before all volatiles are thoroughly removed under reduced pressure to yield Ar*SnH(DMAP) (**5**) in 95% purity as a yellow powder. Analytical data: ^1^H-NMR (C_6_D_6_, 298 K, 500.13 MHz): *δ* = 11.64 (s, 1H, ^1^ *J*_119/117Sn–H_ = 113.2 Hz, Sn–H), 8.32 (d, *o*-C*H*_DMAP_) 7.28 (m, 1H, *p*-C*H*_Ph_), 7.20 (m, 2H, *m*-C*H*_Ph_), 7.15 (s, 4H, *m*-C*H*_trip_), 6.08 (m, *m*-C*H*_DMAP_), 3.40 (sept., 4H, ^3^ *J*_H–H_ = 6.83 Hz, *o*-C*H*Me_2_), 2.87 (sept., 2H, ^3^ *J*_H–H_ = 6.87 Hz, *p*-C*H*Me_2_), 2.27 (s, NC*H*_3_), 1.43 (br d, 12H, ^3^ *J*_H–H_ = 6.81 Hz, *o*-CH*Me*_2_), 1.28 (d, 12H, ^3^ *J*_H–H_ = 6.92 Hz, *p*-CH*Me*_2_), 1.22 (d, 12H, ^3^ *J*_H–H_ = 6.81 Hz, *o*-CH*Me*_2_); ^13^C{^1^H} (C_6_D_6_, 298 K, 100.62 MHz): *δ* = 167.4 (*ipso-C*_*Ph*_), 154.0 (*p-C*_DMAP_), 150.6 (*o-C*_DMAP_), 147.5 (*o-C*_*trip*_), 147.5 (*p-C*_*trip*_), 147.1 (*o-C*_*Ph*_), 140.5 (*ipso-C*_*trip*_), 128.9 (*m-C*_*Ph*_), 125.2 (s, *p-C*_*Ph*_), 120.9 (*m-C*_*trip*_), 106.8 (*m-C*_DMAP_), 38.2 (N*Me*_2_), 34.7 (*p-C*HMe_2_), 31.0 (*o-C*HMe_2_), 26.1 (*o*-CH*Me*_2_), 24.5 (s, *p*-CH*Me*_2_), 23.8 (*o*-CH*Me*_2_); ^119^Sn–^1^H-coupled (C_6_D_6_, 298 K, 93.24 MHz) *δ* = 224.9 (d, ^1^ *J*_119Sn–H_ = 118 Hz). IR (KBr, cm^–1^): likely superimposed: see ESI.† N. B. Since isolation of crystals of Ar*SnH(DMAP) **5** failed so far, we have not been able to provide a sample suitable for elemental analysis.

#### Ar*Sn(DMAP)–SnH_2_Ar* (**6**)

From Ar*SnH_3_ (75 mg) and DMAP (91.1 mg) a mixture of solid [Ar*SnH × 6(DMAP)] is prepared as described above. The yellow solid is extracted with cool (*ca.* 0 °C) pentane (2 × 0.8 mL) and the brightly yellow extracts were concentrated to approx. 1 mL and stored at –40 °C for two days. The residual white powder corresponds to approx. 80% of the DMAP used. From the cooled extracts further amounts of DMAP crystallize as fine colorless needles and the cool supernatant yellow solution is separated with a syringe. When warmed to ambient temperature the solution turns green. Bright yellow crystals of Ar*Sn(DMAP)–SnH_2_Ar* (**6**) (52 mg, 0.039 mmol, 63%) suitable for X-ray diffraction were obtained from this solution after a few days at –40 °C. It seems that **6** can only be isolated in the solid state since in solution, it reveals to be in an equilibrium with **H** and **5**. In VT-NMR experiments at lower temperatures sharp resonances were observed in the temperature range from –30 to –50 °C allowing the identification of **6** in mixtures with **5** and **H**. The resonances reported here correspond to the characteristic signal sets that were assigned to **6** in the spectra of the mixtures, however a thorough assignment of all resonances is circumvented due to overlap with similar ligand related signals for **5** and **H**. The complete spectrum of the mixture can be found in the ESI.† Analytical data: ^1^H-NMR (tol-*d*_8_, 243 K, 500.13 MHz): *δ* = 5.55 (s + satellites, 1H, ^1^ *J*_119Sn–H_ = 1230 Hz, ^1^ *J*_117Sn–H_ = 1176 Hz, Sn–*H*), 5.47 (s + satellites, 1H, ^1^ *J*_119Sn–H_ = 1045 Hz, ^1^ *J*_117Sn–H_ = 997 Hz, Sn–*H*); ^119^Sn–^1^H-coupled (tol-*d*_8_, 243 K, 186.52 MHz) *δ* = 374.4 (br s, 1Sn, *Sn*(DMAP)), –248.0 (dd, 1Sn, ^1^ *J*_119Sn–H_ = 1225 Hz ^1^ *J*_119Sn–H_ = 1043 Hz, *Sn*H_a_H_b_); anal. calcd for [**6**] C_79_H_110_N_2_Sn_2_: C 71.60, H 8.37, N 2.11; found: C 71.77, H 8.15, N 2.17. IR (KBr, cm^–1^): 1763 (s, Sn–H-stretch), 600 (s, Sn–H-bend). N. B. Due to the equilibrium the resulting mixture of similar overlapping compound signals prevents a meaningful assignment of the ^13^C NMR signals.

#### Ar*SnH(py) (**7**)

In a NMR experiment, a J. Young-NMR tube is charged with a suspension of Ar*SnH_3_ (30 mg, 0.050 mmol) in *d*_5_-pyridine (0.5 mL). Upon heating the cloudy solid instantly goes into solution and the mixture is kept at 95 °C for 4 h with obvious hydrogen evolution to yield a clear bright yellow solution of Ar*SnH(py) with approx. 4% of distannane (**4**) being formed as side-product. Analytical data: ^1^H-NMR (py-*d*_5_, 298 K, 500.13 MHz): *δ* = 12.13 (s + satellites, 1H, ^1^ *J*_119/117Sn–H_ = 99 Hz, Sn–*H*) 8.76 (s, pyridine), 7.61 (s, pyridine), 7.50 (t, 1H, ^3^ *J*_HH_ = 7.33 Hz, *p*-C*H*_Ph_), 7.32 (d, 2H, ^3^ *J*_HH_ = 7.33 Hz, *m*-C*H*_Ph_), 7.25 overlaying with solvent signal (s, 4H, *m*-C*H*_trip_), 3.37 (br sept., 4H, ^3^ *J*_HH_ = 6.75 Hz, *o*-C*H*Me_2_), 3.00 (br sept., 2H, ^3^ *J*_HH_ = 6.95 Hz, *p*-C*H*Me_2_), 1.38 (d, 12H, ^3^ *J*_H–H_ = 6.95 Hz, *p*-CH*Me*_2_), 1.37 (d, 12H, ^3^ *J*_H–H_ = 6.75 Hz, *o*-CH*Me*_2_), 1.20 (d, 12H, ^3^ *J*_H–H_ = 6.75 Hz, *o*-CH*Me*_2_).^13^C{^1^H} (py-*d*_5_, 298 K, 100.62 MHz): *δ* = 167.4 (*ipso-C*_*Ph*_), 150.2 (py-*d*_5_), 148.4 (*p-C*_*trip*_), 147.7 (*o-C*_*Ph*_), 147.4 (*o-C*_*trip*_), 140.0 (*ipso-C*_*trip*_), 135.8 (py-*d*_5_), 129.2 (*m-C*_*Ph*_), 125.9 (s, *p-C*_*Ph*_), 123.8 (py-*d*_5_), 121.4 (*m-C*_*trip*_), 34.8 (*p-C*HMe_2_), 31.2 (*o-C*HMe_2_), 26.3 (*o*-CH*Me*_2_), 24.7 (s, *p*-CH*Me*_2_), 23.9 (*o*-CH*Me*_2_) ^119^Sn–^1^H-coupled (py-*d*_5_, 298 K, 186.55 MHz) *δ* = 283 (d, ^1^ *J*_119Sn–H_ = 105 Hz), a second minor signal (approx. 30%) is observed at *δ* = 279 ppm. ^1^H NMR does not contain signal sets for a second Sn–H compound in the solution. According to chemical trapping experiments, this signal corresponds to an isomeric form of Ar*SnH(py) (**7**), since it completely vanishes upon reaction with 1,2,3,4-tetramethyl fulvene forming only one product Ar*SnCp* **8**.

#### Ar*SnCp* (**8**)

##### Method A

A solution of Ar*SnH_3_ (100 mg, 0.1657 mmol, 1 eq.) and DMAP (122 mg, 1.00 mmol, 6 eq.) in benzene (3 mL) is stirred at ambient temperature for 8 h. A solution of 1,2,3,4-tetramethyl fulvene (25.6 mg, 0.190 mmol. 1.15 eq.) in benzene (0.5 mL) is added and the solution is stirred for 30 min before all volatiles are removed *in vacuo*. The yellow residue is extracted with cool pentane (0 °C, *ca.* 1 mL) and the bright yellow extract is stored at –40 °C for a few hours to furtherly crystallize residual DMAP as colorless plates. The cool supernatant yellow solution is separated *via* syringe and the volatiles are removed to obtain Ar*SnCp* (**8**) as a yellow powder (95% purity) (105 mg, 0.143 mmol, 86%). Crystals suitable for X-ray diffraction were obtained from saturated solutions in hexane or pentane at –40 °C.

##### Method B

A solution of Ar*SnH_3_ (75 mg, 0.124 mmol, 1 eq.) and Et_2_NMe (*ca.* 210 mg, 2.41 mmol, 20 eq.) in benzene (2 mL) is stirred at ambient temperature for 6 h. Successively, a solution of 1,2,3,4-tetramethyl fulvene (19.2 mg, 0.190 mmol, 1.15 eq.) in benzene (0.5 mL) and 0.1 mL dry pyridine are added to the deep blue solution. On addition of pyridine the mixture immediately turns bright yellow. The solution is stirred for 30 min before all volatiles are removed *in vacuo* to obtain Ar*SnCp* (**8**) as a yellow powder (>95% purity) (87 mg, 0.118 mmol, 95%). Analytical data: ^1^H-NMR (C_6_D_6_, 298 K, 400.11 MHz): 7.26 (s, 4H, *m*-C*H*_trip_), 2.73–2.17 (m, 3H, *o*/*p*-C*H*_Ph_), 3.13 (sept., 4H, ^3^ *J*_H–H_ = 6.80 Hz, *o*-C*H*Me_2_), 2.90 (sept., 2H, ^3^ *J*_H–H_ = 6.95 Hz, *p*-C*H*Me_2_), 1.76 (s, 15H, C_5_–C*H*_3_), 1.47 (d, 12H, ^3^ *J*_H–H_ = 6.83 Hz, *o*-CH*Me*_2_), 1.33 (d, 12H, ^3^ *J*_H–H_ = 6.94 Hz, *p*-CH*Me*_2_), 1.22 (d, 12H, ^3^ *J*_H–H_ = 6.80 Hz, *o*-CH*Me*_2_); ^13^C{^1^H} (C_6_D_6_, 298 K, 100.62 MHz): *δ* = 195.7 (*ipso-C*_*Ph*_), 148.6 (*p-C*_*trip*_), 147.0 (*o-C*_*trip*_), 144.9 (*o-C*_*Ph*_), 138.1 (*ipso-C*_*trip*_), 131.2 (*m-C*_*Ph*_), 125.8 (*p-C*_*Ph*_), 121.6 (*m-C*_*trip*_), 117.8 (Cp*–*C*_5_), 35.0 (*p-C*HMe_2_), 31.05 (*o-C*HMe_2_), 26.8 (*o*-CH*Me*_2_), 24.4 (*p*-CH*Me*_2_), 23.1 (*o*-CH*Me*_2_), 10.4 (C_5_–*C*H_3_); ^119^Sn{^1^H} (C_6_D_6_, 298 K, 93.24 MHz) *δ* = –26 (s). Anal. calcd for [**8**] C_46_H_64_Sn: C 75.10, H 8.77; found: C 75.17, H 8.61.
