# Supplementary material for: A nitrogen-base catalyzed generation of organotin(ii) hydride from an organotin trihydride under reductive dihydrogen elimination
Source: Chem Sci. 2015 May 22;6(8):4737–51. doi: 10.1039/c5sc01561h (PMC5667502; doi:10.1039/c5sc01561h)
Supplement: Supplementary file 1 [file SC-006-C5SC01561H-s001.pdf]

Electronic Supplementary Material (ESI) for Chemical Science.
This journal is © The Royal Society of Chemistry 2015

Supporting Information

A nitrogen-base catalyzed generation of organotin(II) hydride from organotin trihydride under reductive dihydrogen elimination

Christian P. Sindlinger[a], Andreas Stasch[b], Holger F. Bettinger[c] and Lars Wesemann[a,*]

[a]Institut für Anorganische Chemie, Auf der Morgenstelle 18, 72076 Tübingen, Germany

[b]School of Chemistry, Monash University, PO Box 23, Melbourne, VIC 3800, Australia

[c]Institut für Organische Chemie, Auf der Morgenstelle 18, 72076 Tübingen, Germany

Table of Contents

Experimental Details 2

General Information 2

NMR spectroscopy 2

Crystallographic Details 3

Refinement Details 3

X-Ray Diffraction and Refinement of Ar*SnH_3 1 3

X-Ray Diffraction and Refinement of Ar*Sn(DMAP)SnH_2Ar* 6 4

X-Ray Diffraction and Refinement of Ar*SnCp* 8 4

X-Ray Diffraction on Distannane 4 4

Synthesis 5

Ar*$SnCl_3$ (2) 5

Ar*SnD_3 (1-D) and Ar*SnH_3 (1) 5

Kinetic Experiments 6

Determination of the order in DMAP 6

Arrhenius-analysis approximation of the activation energy 7

Kinetic Isotope Effects (KIE) 9

KIE in d_6-benzene 10

KIE in d_8-THF 10

NMR Spectra 11

Compound 1 Ar*SnH_3 and Ar*SnD_3 as used as starting material for kinetic dehydrogenation experiments 11

Distannane (4) ^{1}H-NMR 11

Distannane (4) ^{119}Sn-^{1}H-coupled-NMR 12

Ar*SnH_3 plus DMAP over time 12

Ar*SnH(DMAP) ^{119}Sn-^{1}H coupled NMR 13

Ar*Sn(DMAP)SnH_2Ar* ^{1}H-NMR 13

Ar*Sn(DMAP)SnH_2Ar* ^{1}H-NMR-details -30°C 14

Dissolved crystals of Ar*Sn(DMAP)SnH_2Ar* ^{119}Sn -^{1}H-coupled-NMR 14

Dissolved crystals of Ar*Sn(DMAP)SnH_2Ar* ^{119}Sn-NMR at RT 15

Raw solution ^{1}H-NMR of TMEDA catalysed dehydrogenation 15

Raw solution ^{1}H-NMR of Et_2NMe catalysed dehydrogenation 16

Consecutive addition of DMAP to in situ generated Ar*SnH solutions 16

^{119}Sn-NMR of Ar*SnH + 1 eq DMAP 17

Selected IR Spectra 18

Ar*SnH_2-SnH_2Ar* 18

Ar*SnH(DMAP) / Ar*SnH(6DMAP) / Ar*SnH_2Sn(DMAP)Ar* 18

Ar*SnH(DMAP) / Ar*SnD(DMAP) 19

Computational Details 20

General Methodology and BDE calculations 20

Mechanism Study 21

Cartesian coordinates of the optimized structure of $PhSnH_3$, DMAP, Pyridine, NMe_3 22

Cartesian coordinates of the optimized structure of LA, LA(py), TS-A, TS-A(py), TS-B1(DMAP), TS-B1(Py), TS-B1(NMe_3) 22

Cartesian coordinates of the optimized structures of Ar*SnH_3, Ar*SnH, µ-bridged $(Ar*SnH)_2$, Ar*SnH(DMAP), Ar*SnH(py), Ar*SnH(NMe_3) 25

Literature 33

Experimental Details

General Information

All manipulations were carried out under argon atmosphere using standard Schlenk techniques or an MBraun Glovebox. THF, diethylether and benzene were distilled from sodium/benzophenone, toluene from sodium. Hexane and pentane were obtained from an MBRAUN solvent purification system and degassed. Benzene-d_6 was distilled from sodium and stored over potassium, THF-d_8 was distilled from $LiAlD_4$ and stored under exclusion of light at –40°C, pyridine-d_5 was destilled from calcium hydride and subsequently from sodium. DMAP was obtained commercially (99%, Aldrich) and used without further purification, *i*-Pr_2Net, Et_2NMe and TMEDA were obtained commercially (Aldrich) and distilled from CaH_2 (*i*-Pr_2NEt, Et_2NMe) or from *n*-BuLi (TMEDA) and degassed.

Terphenyl- iodide (Ar*I)[1], -lithium-etherate (Ar*Li(OEt_2))[1], -Sn(II) chloride (Ar*SnCl)[2] and trihydride (Ar*SnH_3)[3] were prepared according to slightly modified literature procedures. 1,2,34-Tetramethylfulvene was prepared according to a literature procedure.[4]

Elemental analysis was performed by the Institut für Anorganische Chemie, Universität Tübingen using a Vario MICRO EL analyzer.

NMR spectroscopy

NMR spectra were recorded with either a Bruker DRX-250 NMR spectrometer equipped with a 5 mm ATM probe head and operating at 250.13 (^{1}H), 62.90 (^{13}C) 93.3 MHz (^{119}Sn), a Bruker AvanceII+400 NMR spectrometer equipped with a 5 mm

QNP (quad nucleus probe) head and operating at 400.13 (^{1}H), 161.97 (^{31}P) 100.62 MHz (^{13}C) or a Bruker AVII+ 500 NMR spectrometer with a 5 mm ATM probe head or a 5 mm TBO probe head and operating at 500.13 (^{1}H), 125.76 (^{13}C), 186.5 MHz (^{119}Sn) and a low-temperature set-up. The proton and carbon signals were assigned where possible via a detailed analysis of ^{1}H, ^{13}C, ^{13}C-UDEFT, ^{1}H-^{1}H COSY, ^{1}H-^{13}C HSQC, ^{1}H-^{13}C HMBC spectra.

Crystallographic Details

Refinement Details

X-ray data for **1, 6** and **8** were collected with a Bruker Smart APEX II diffractometer with graphite- monochromated Mo Kα radiation. The programs used were Bruker's APEX2 v2011.8-0 including SADABS for absorption correction and SAINT for structure solution, as well as the ShelXLE graphical user interface for shelxl for structure refinement.[5-9] For further refinement details see the attached cif-files.

Table 1. Selected crystallographic data for compounds.

Compound	**1**	**6 × 2(C_5H_{12})**	**8 × 0.5(C_5H_{12})**
CCDC number	1061347	1061348	1061349
Empirical formula	$C_{36}H_{52}Sn$	$C_{79}H_{110}N_2Sn_2$ ×2 (C_5H_{12})	$C_{64}H_{70}Sn$×0.5 (C_5H_{12})
Formula weight	602.51	1469.36	771.73
T [K]	100(2)	100(2)	100(2)
Λ [Å]	0.71073	0.71073	0.71073
Crystal system	Orthorhombic	triclinic	Monoclinic
Space group	Pbcm	*P–1*	*P2₁/c*
a [Å]	10.9013(2)	12.7122(5)	15.9849(4)
b [Å]	12.0601(2)	15.3521(6)	15.9056(4)
c [Å]	25.2220(5)	22.5773(9)	17.7408(4)
α [°]	90	84.881(2)	90
β [°]	90	89.932(2)	100.7540(10)
γ [°]	90	82.556(2)	90
V [Å^3]	3315.96(11)	4351.4(3)	4431.37(19)
Z	8	2	4
ρ [Mg m^{-3}]	1.209	1.121	1.157
μ [mm^{-1}]	0.791	0.614	0.606
F(000)	1272	1564	1664
Crystal size [mm^3]	0.13 × 0.18 × 0.24	0.134 × 0.164 × 0.274	0.11 × 0.16 × 0.29
Theta range [°]	1.87 – 27.90	1.86 – 26.88	1.82 – 27.12
Index ranges	–14 ≤ h ≤ 14	–16 ≤ h ≤ 16	–20 ≤ h ≤ 20
	–15 ≤ h ≤ 15	–18 ≤ h ≤19	–20 ≤ h ≤19
	–33 ≤ h ≤ 33	–27 ≤ h ≤ 28	–21 ≤ h ≤ 22
Refl. collected	42055	51088	55843
Indep. refl. / [R(int)]	4053/ 0.0297	18495 / 0.0344	9783 / 0.0332
Completeness to theta max	100.0%	98.5%	99.7%
Data/restraints/parameter	4053/3/183	18495/291/991	9783/74/505
GooF	1.063	1.051	1.011
Final *R* indices [*I* >2sigma(*I*)] *R*1 / *wR*2	0.0208 / 0.0557	0.0469 / 0.1159	0.0303 / 0.0705
R indices (all data) *R*1 / *wR*2	0.0260/ 0.0584	0.0672/ 0.1257	0.0448 / 0.0767
Largest diff. peak and hole [eA^{-3}]	0.475 / –0.467	3.805/ –0.903	0.893 / –0.854
Absorption correction	numerical	numerical	numerical

X-Ray Diffraction and Refinement of Ar*SnH_3 1

Large colorless crystals of Ar*SnH_3 were obtained quantitatively from slow solvent evaporation from solutions in benzene. The Sn–H protons were located in the difference Fourier map and found to be disordered. They were treated with DFIX and DANG commands and the Sn–H bond length are underestimated.

X-Ray Diffraction and Refinement of Ar*Sn(DMAP)SnH$_2$Ar* 6

Molecule **6** crystallized from pentane at –40°C. In the crystal structure of **6** two equivalents of lattice pentane were found in the asymmetric unit located on three positions. One position was found to be fully occupied by one whole molecule of pentane with severe disorder that was treated with DFIX and DANG commands. The other two positions are only occupied to 50% by severely disordered pentane molecules which were treated using DFIX, DANG; SIMU, DELU, ISOR as well as EADP command. A large residual density of 3.8 e$Å^{-3}$ remained around Sn1. The methyl group in a para-*i*Pr-group at the Trip moieties attached to the stannylene-Sn was found to be disordered over two positions and treated accordingly. Sn–H hydrogen atoms have been located in the difference Fourier map. The found Sn–H bond length may therefore be underestimated.

X-Ray Diffraction and Refinement of Ar*SnCp* 8

Molecules **8** crystallized reproducibly in good yields from pentane or hexane at –40°C. A half equivalent of lattice pentane was found per formula unit which is severely disordered and treated with DFIX, DANG, SIMU, DELU, ISOR commands. An isopropylgroup in the Trip-moieties was found to be disordered and treated accordingly.

X-Ray Diffraction on Distannane 4

Distannane **4** repeatedly crystallized from saturated solutions in benzene in large colorless blocks that diffracted poorly. To date we were not able to grow crystals suitable for X-ray diffraction from various conditions. The data sets obtained for the crystals were refined isotropically in the monoclinic space group P2/n. The asymmetric unit of the obtained poor preliminary solution and refinement up to a wR2 = 44% contained five independent molecules. Proton refinement is impossible. The preliminary structure refinement supports the connectivity pattern that is evident from the NMR spectroscopic properties of the compound.

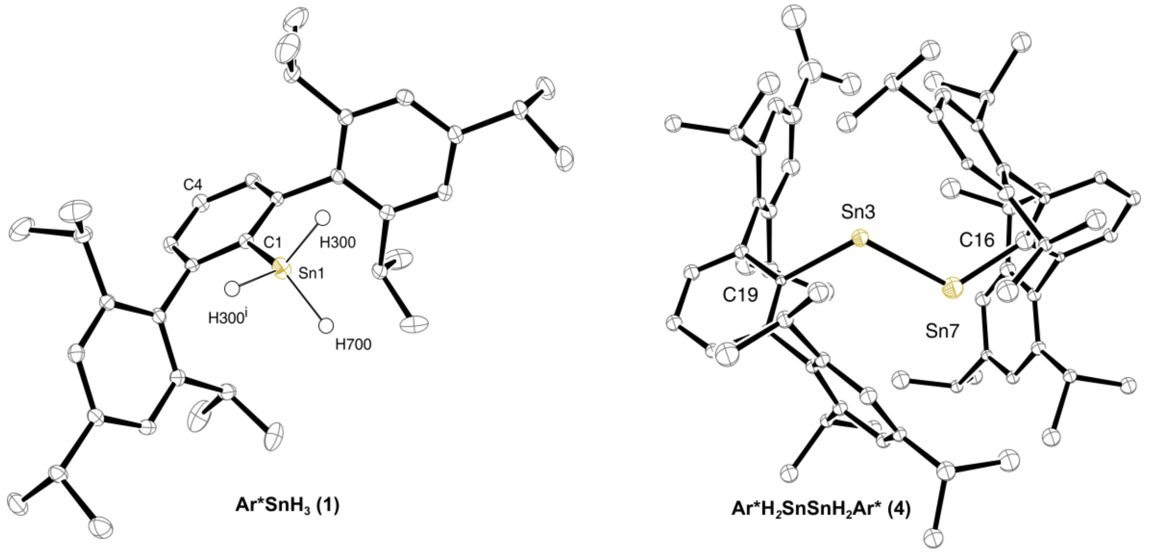

Figure 1SI

(*left*) ORTEP plot for Ar*SnH$_3$ **1**. Hydrogen atoms except Sn-bond H were omitted for clarity. Thermal ellipsoids are drawn at 50% probability level. Selected bond lengths [Å] are given: Sn1–C1 2.157(2), Sn–H300/700 1.493(15)/1.529(17).

(*right*) Isotropically refined preliminary solution for distannane **4**. One out of five molecules in the asymmetric unit is depicted. Atom positions were refined isotropically and are depicted at 25% probability level. Selected bond lengths [Å] and angles [°] are given exemplarily, the ranges for the other molecules are given in square brackets: Sn3–Sn7 2.272 [2.685-2.764], C19-Sn3-Sn7-C16 159.5 [155.4-167.4], C19-Sn3-Sn7 117.0, Sn3-Sn7-C16 120.3 [114.6-121.5].

Synthesis

Ar*SnCl$_3$ (2)

Caution! Mercury compounds are toxic! **Caution!** To a mixture of solid Ar*SnCl (**3**) (1.592 g, 2.503 mmol, 1 eq) and $HgCl_2$ (0.680 g, 2.505 mmol, 1 eq) toluene (35 mL) was added at room temperature and the initially bright orange suspension immediately decolourizes and a blackish precipitate is observed. Volatiles are removed under reduced pressure and the residue is extracted with hexane (6 × 40 mL), the supernatant extracts are decanted and filtered through a plug of glass fibre. After removal of hexane under reduced pressure pure Ar*SnCl$_3$ (**2**) is obtained as a white fluffy powder (1.698 g, 2.403 mmol, 96%). Spectroscopic data were identical to those reported in the literature.[10]

Ar*SnD$_3$ (1-D) and Ar*SnH$_3$ (1)

Ar*SnD$_3$ was synthesized essentially identical to Ar*SnH$_3$ using $LiAlD_4$ instead of $LiAlH_4$. During the reaction direct day- or lamp light were avoided. To a stirred suspension of $LiAlD_4$ (Aldrich 98 %D, 94 mg, 2.2 mmol, 3.1 eq) in Et_2O (10 mL) at –40°C a solution of Ar*SnCl$_3$ (**2**) (500 mg, 0.707 mmol, 1 eq) in Et_2O (15 mL) was added and the mixture was stirred at –40°C for 2h before the mixture was allowed to warm to room temperature. The mixture is kept at room temperature for 1 h before Et_2O was removed under reduced pressure and the grey residue was dried well in vacuo. The residue is repeatedly extracted with hexane (in total 90 mL) and the decanted extracts were filtered through a short plug of Celite or a syringe filter. Volatiles were removed under reduced pressure to obtain pure Ar*SnD$_3$ as a white powder (414 mg, 0.683 mmol, 97%). Spectroscopic properties are identical to those reported for Ar*SnH$_3$ with the respective deuterium related couplings: **^{1}H**-NMR (C_6D_6, 298 K, 400.11 MHz): δ = 7.25-7.21 (m, 3H, *p/m*-C*H*$_{Ph}$), 7.19 (s, 4H, *m*-C*H*$_{trip}$), 2.91 (sept., 4H, $^{3}J_{H\text{-}H}$ = 6.87 Hz, *o*-C*H*Me$_2$), 2.76 (sept, 2H, $^{3}J_{H\text{-}H}$ = 6.94 Hz, *p*-C*H*Me$_2$), 1.33 (d, 12H, $^{3}J_{H\text{-}H}$ = 6.94 Hz, *o*-CH*Me$_2$*), 1.27 (d, 12H, $^{3}J_{H\text{-}H}$ = 6.91 Hz, *o*-CH*Me$_2$*), 1.14 (d, 12H, $^{3}J_{H\text{-}H}$ = 6.83 Hz, *p*-CH*Me$_2$*) **^{119}Sn** (C_6D_6, 298K, 93.25 MHz) –388.3 (sept., SnD_3, $^{1}J_{119Sn\text{-}D}$ = 296.1 Hz). IR (KBr, cm^{-1}): 1340 (stretch, Sn–D).

Kinetic Experiments

Determination of the order in DMAP

All experiments were conducted using 18.0 mg Ar*SnH_3 in 0.65 mL of C_6D_6 (with approx. 1-2% of hexamethyldisiloxane as internal standard). All signals were manually integrated. To approximate the error of the method selected datasets were repeatedly integrated and the deviation of the determined rate constants was approx. ±2%.

In order to determine the order of the dehydrogenation reaction in DMAP to gain first insights into the general role of the base, the kinetics of the consumption of Ar*SnH_3 in a C_6D_6 solution under pseudo-first order conditions with excessive DMAP concentrations (3, 4, 6, 8, 10 equivalents) were measured by means of time-dependent ^{1}H NMR spectroscopy at 50°C. Manual integration of the hydride resonance at δ = 4.21 ppm of Ar*SnH_3 provided [Ar*SnH_3]$_t$ proportional values. Integration values were referenced to the integral of the internal standard ($Me_3SiOSiMe_3$). Samples (in a J- Young-type NMR tube) have been prepared and mixed at room temperature in a glovebox and then transferred (generally within 3-4 minutes) to a preheated (50°C) NMR spectrometer (Bruker AVII+ 500 NMR). The time was counted from the moment the sample was inserted into the spectrometer (0:00) at the investigated temperature. Each ^{1}H NMR was taken with 16 scans and spectra acquisition was repeated after exactly 300 seconds.

The values [Ar*SnH_3](t) depicted in the following graph were normalized for each experiment against the first determined value [Ar*SnH_3](0) = 1.

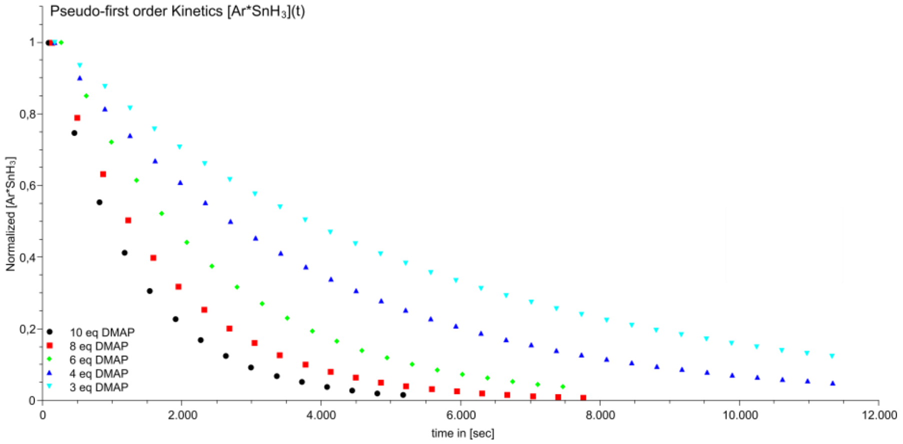

From the rate law of a pseudo-first order reaction (with R and Ar* being equivalently used)

$$d[RSnH_3] = -k'[RSnH_3] \text{ with } k' = k[DMAP] \text{ for } [DMAP] \gg [RSnH_3]$$

follows after integration

$$ln[RSnH_3]_t = \ln[RSnH_3]_0 - k't$$

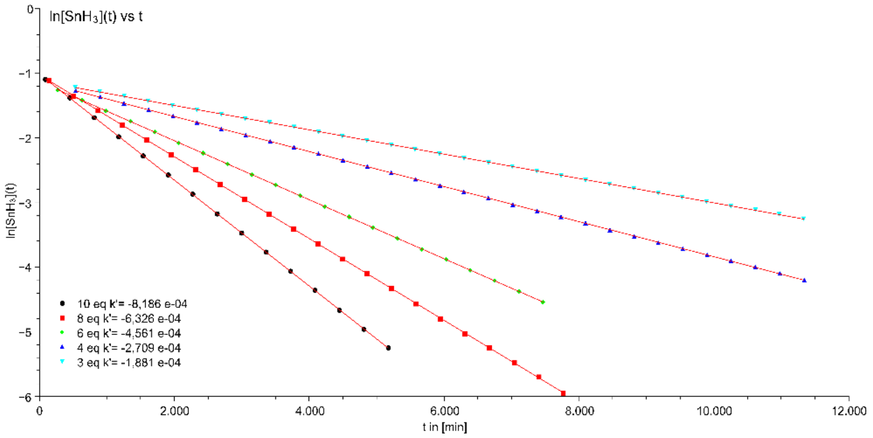

The plotted values for ln[RSnH_3] to the not normalized integral values of [Ar*SnH_3] referenced to the standard integral = 1.

Linear fitting according to the equation

$$y = m\,x + b$$

provides the pseudo rate constant k’

$$k' = m$$

The results of these linear fits for the slope values are given in the legend of the ln[Ar*SnH_3](t) plot.

A direct proportional dependency of the pseudo rate constant from the relative concentration of DMAP is evident and therefore strongly indicates the reaction to be first order in DMAP.

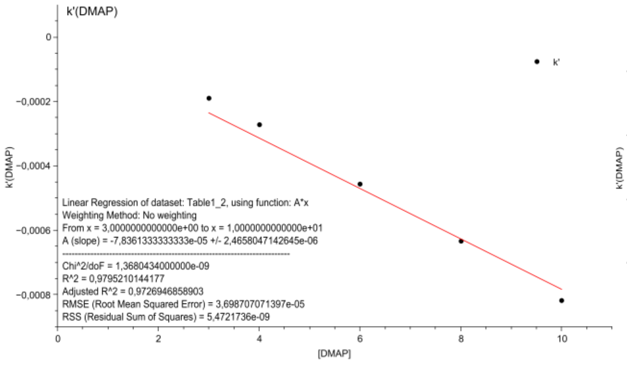

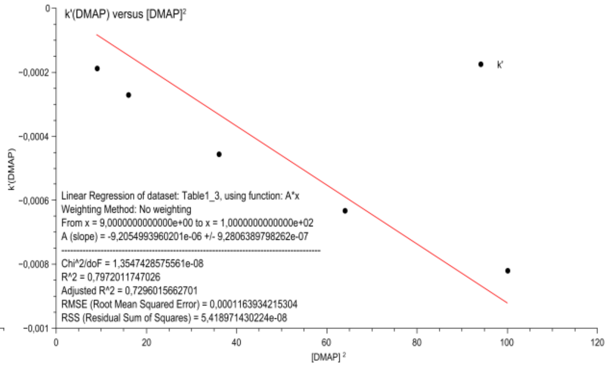

Arrhenius-analysis approximation of the activation energy

Likewise, in order to approximate the activation energy of the reaction an Arrhenius-Analysis was performed on the basis of kinetic data obtained from time-dependent ^{1}H NMR spectroscopy. Therefore samples of 18.0 mg Ar*SnH_3 in 0.65 mL of C_6D_6 (with approx. 1-2% of hexamethyldisiloxane as internal standard) and 6, 8 and 10 equivalents of DMAP (pseudo first order conditions) have been prepared in a glove box and directly transferred (within 3-4 min) in an NMR spectrometer (Bruker AVII+ 500 NMR) preheated to 40°C, 50°C and 60°C. 16 scan spectra were taken every 300 seconds starting from the time the sample was inserted into the spectrometer (time 0:00). By manually integrating the Ar*SnH_3 concentration proportional signal at δ = 4.21 ppm the consumption of Ar*SnH_3 over time was measured. The pseudo-rate constants $k'_{T,c}$ at each temperature and DMAP concentration were determined as described above by linear regression of ln[Ar*SnH_3] vs t plots.

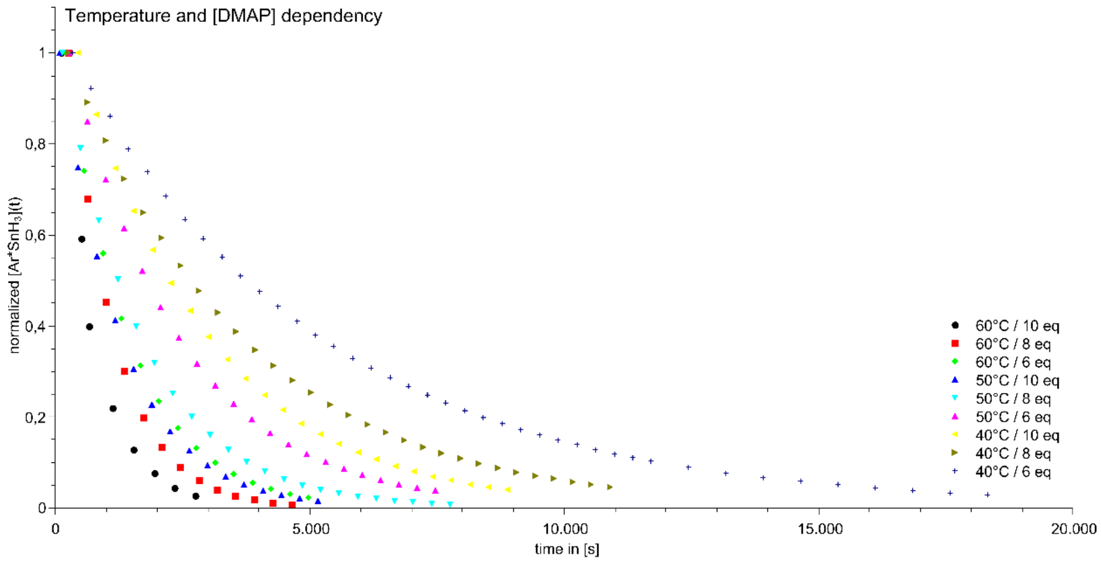

From linear regression of the respective ln[Ar*SnH$_3$](t) vs. time-plots the pseudo rate constants $k'_{T,c}$ were determined.

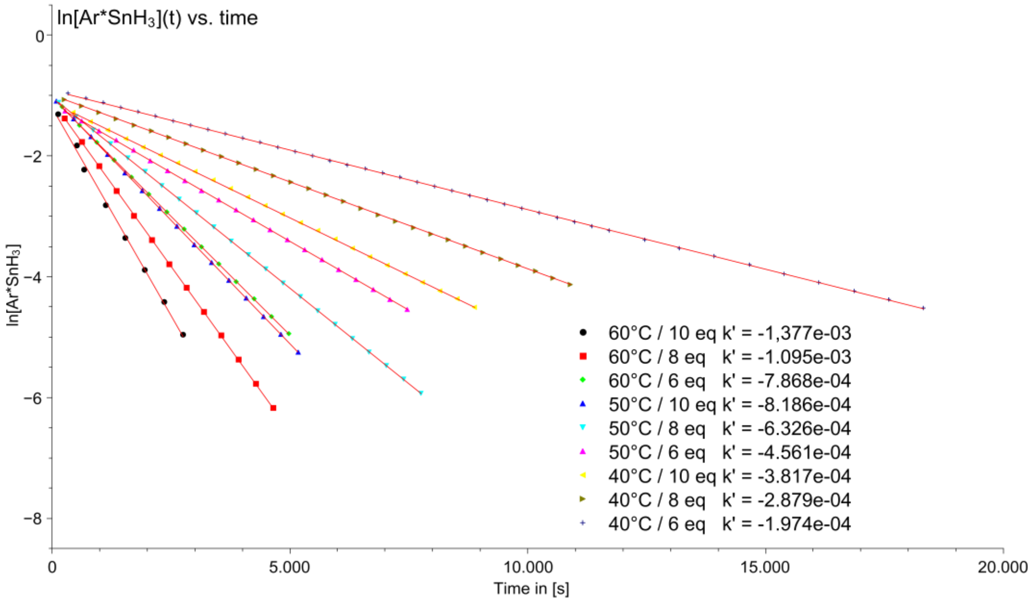

The DMAP independent rate constants k were extrapolated from the k' plots versus DMAP.

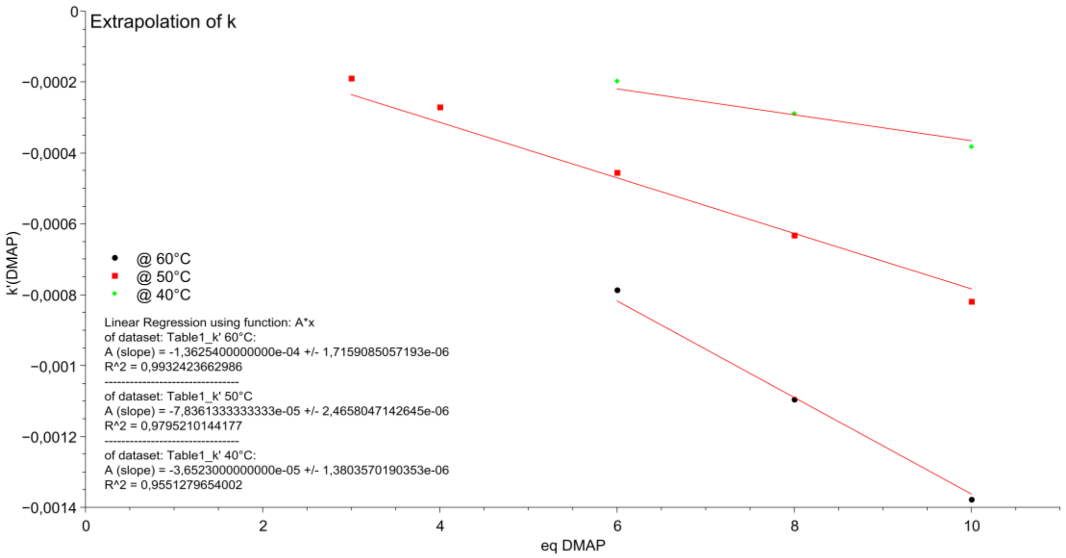

According to the Arrhenius approximation for the temperature dependence of rate constants

$$k_T = A\, e^{-E_a/RT} \text{ with } A = const.$$

The activation energy of a reaction can be approximated by a linearization:

$$\ln(k_T) = \frac{-E_a}{R}\frac{1}{T} + \ln A$$

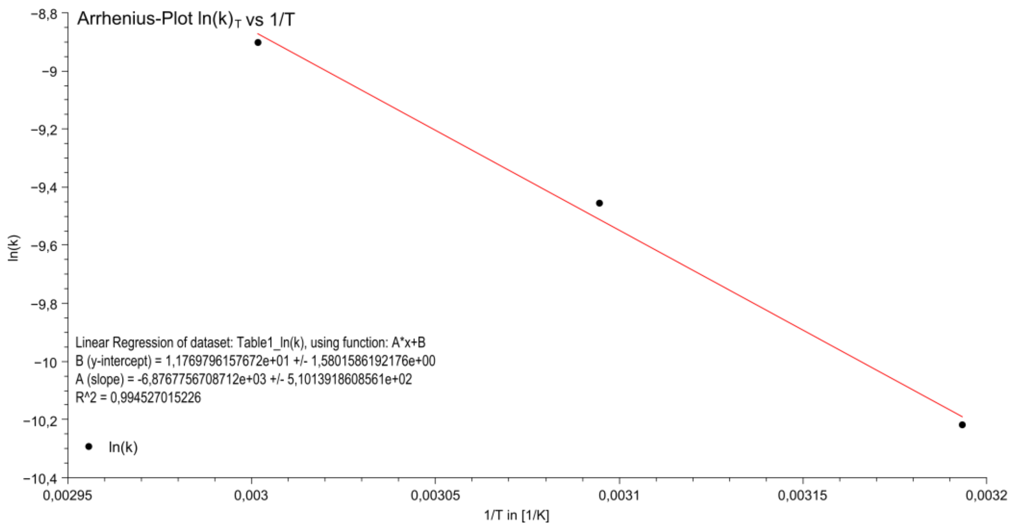

with slope = *m* and the ideal gas constant R = 8,314 J/molK and 4,18 J/cal

$$E_a = -\, m\, R = \; 13.68 \pm 1.01\; kcal/mol$$

Kinetic Isotope Effects (KIE)

In order to approximate the kinetic isotope effect of the reaction time-dependent ^{1}H NMR spectroscopy experiments were performed. Therefore samples of 18.0 mg Ar*SnH_3 or Ar*SnD_3 in 0.65 mL of C_6D_6 or THF-d_8 (with approx. 1-2% of hexamethyldisiloxane as internal standard) and 6 equivalents of DMAP (pseudo first order conditions) have been prepared in a glove box and directly transferred (within 3-4 min) in an NMR spectrometer (Bruker AVII+ 500 NMR) preheated to 40°C. 16 scan spectra were taken every 300 seconds starting from the time the sample was inserted into the spectrometer (time 0:00). By manually integrating the Ar*SnH_3 concentration proportional signal at δ = 4.21 ppm the consumption of Ar*SnH_3 over time was measured. The Ar*SnD_3 concentration was determined by manual integration of the doublet signal *i*Pr-group at δ = 1.11 ppm (C_6D_6) or the *m*-CH_{Trip}-signal (d_8-THF). The pseudo-rate constants k' were determined as described above by linear regression of ln[Ar*SnH_3] vs time plots.

$$KIE = \frac{k'_H}{k'_D}$$

The maximal KIE for cleavage of the Sn–H/Sn–D bond was approximated on account of the different bond strength obtained from the IR(KBr) stretching frequencies of the Sn–H/D at 298K within the Arrhenius relation for k.

$$\tilde{\nu}(SnH) = 1875\; cm^{-1}$$

$$\tilde{\nu}(SnD) = 1342\; cm^{-1}$$

$$k_H = A(H)\; e^{-E_a(H)/kT} \text{and } k_D = A(D)\; e^{-E_a(D)/kT}$$

with

$$E_a(H/D) \approx E_0(Sn - H/Sn - D) = \tfrac{1}{2} h\nu_0 \;\text{ and }\; A(H) \approx A(D)$$

$$\left(\frac{k_H}{k_D}\right)_{th} = e^{\Delta E_A(H-D)/kT} = e^{h[\nu_0(H) - \nu_0(D)]/2kT}$$

$$\left(\frac{k_H}{k_D}\right)_{313K} = 3.40$$

KIE in d_6-benzene

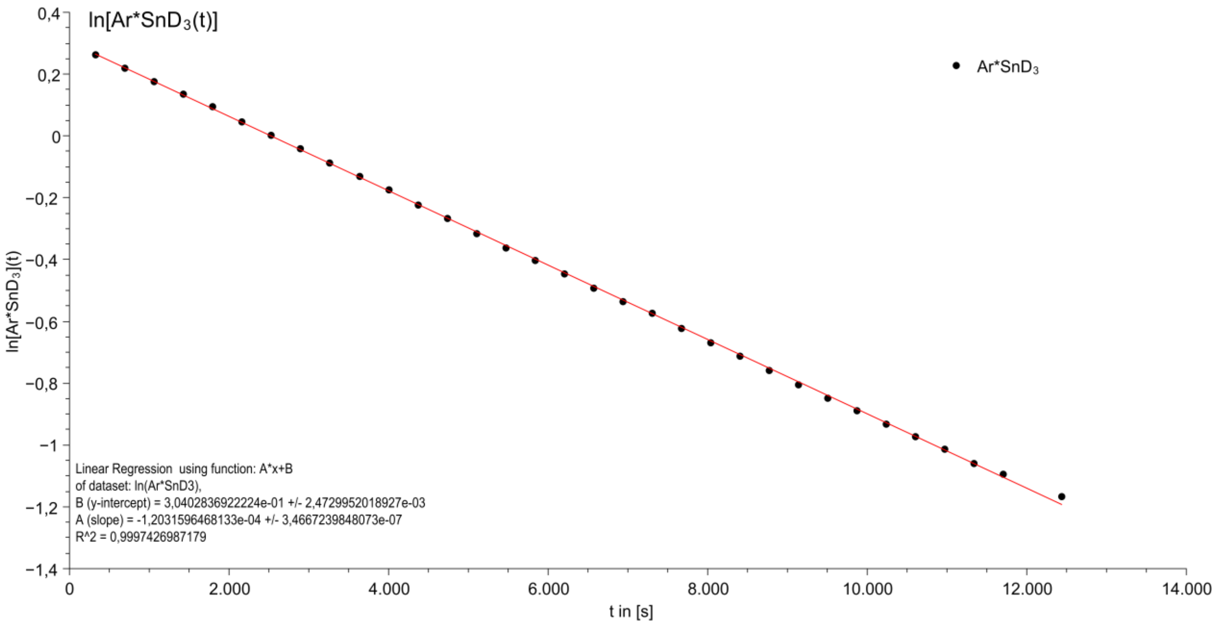

Along with the respective k' value for 6eq DMAP at 40°C for Ar^*SnH_3 (vide supra):

$$KIE(benzene) = \left(\frac{k'_H}{k'_D}\right)_{313K} = \left(\frac{-1.974}{-1.203}\right) = 1.64$$

KIE in d_8-THF

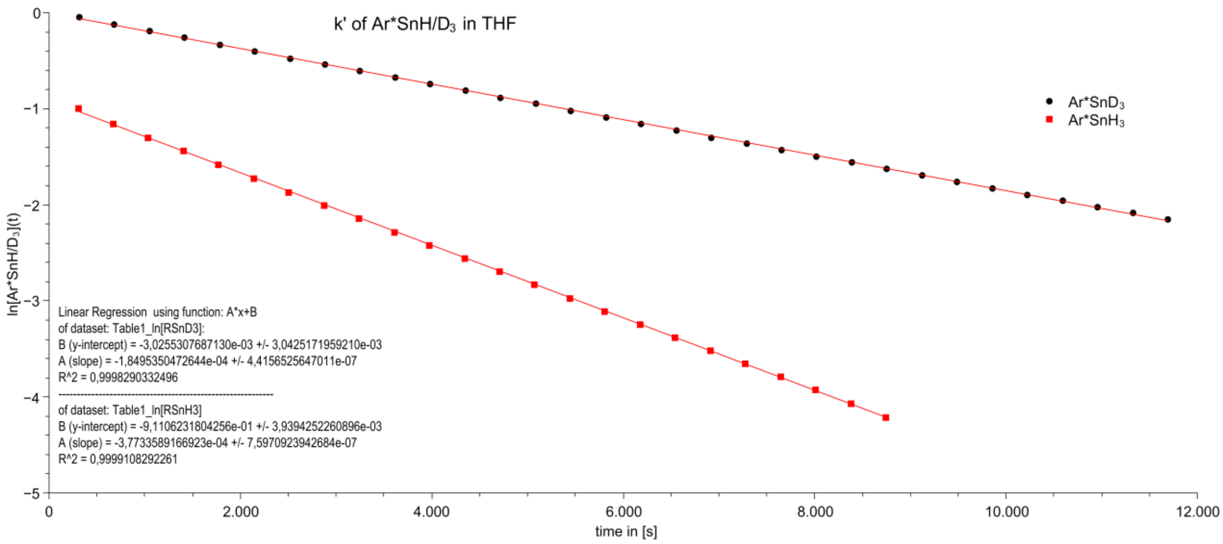

And therefore:

$$KIE(THF) = \left(\frac{k'_H}{k'_D}\right)_{313K} = \left(\frac{-3.773}{-1.850}\right) = 2.04$$

NMR Spectra

Compound 1 Ar*SnH$_3$ and Ar*SnD$_3$ as used as starting material for kinetic dehydrogenation experiments

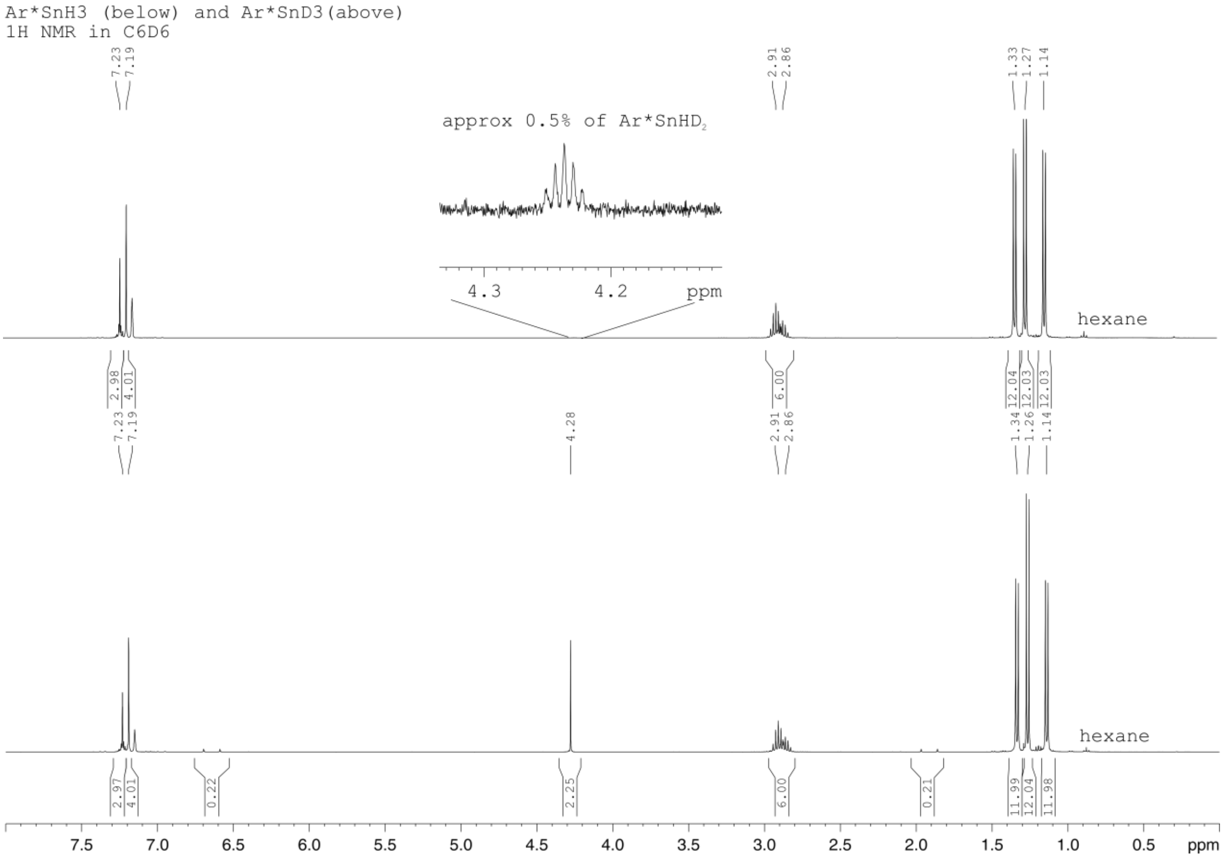

Distannane (4) ^{1}H-NMR

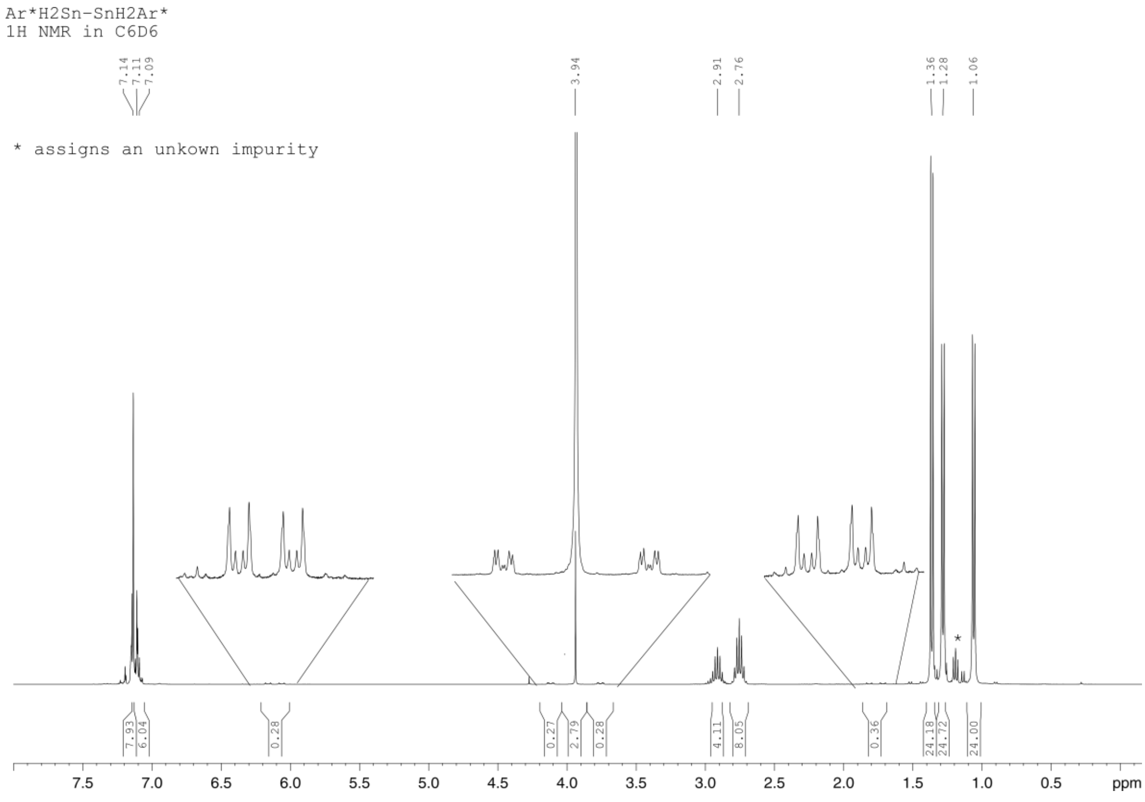

Distannane (4) ^{119}Sn-^{1}H-coupled-NMR

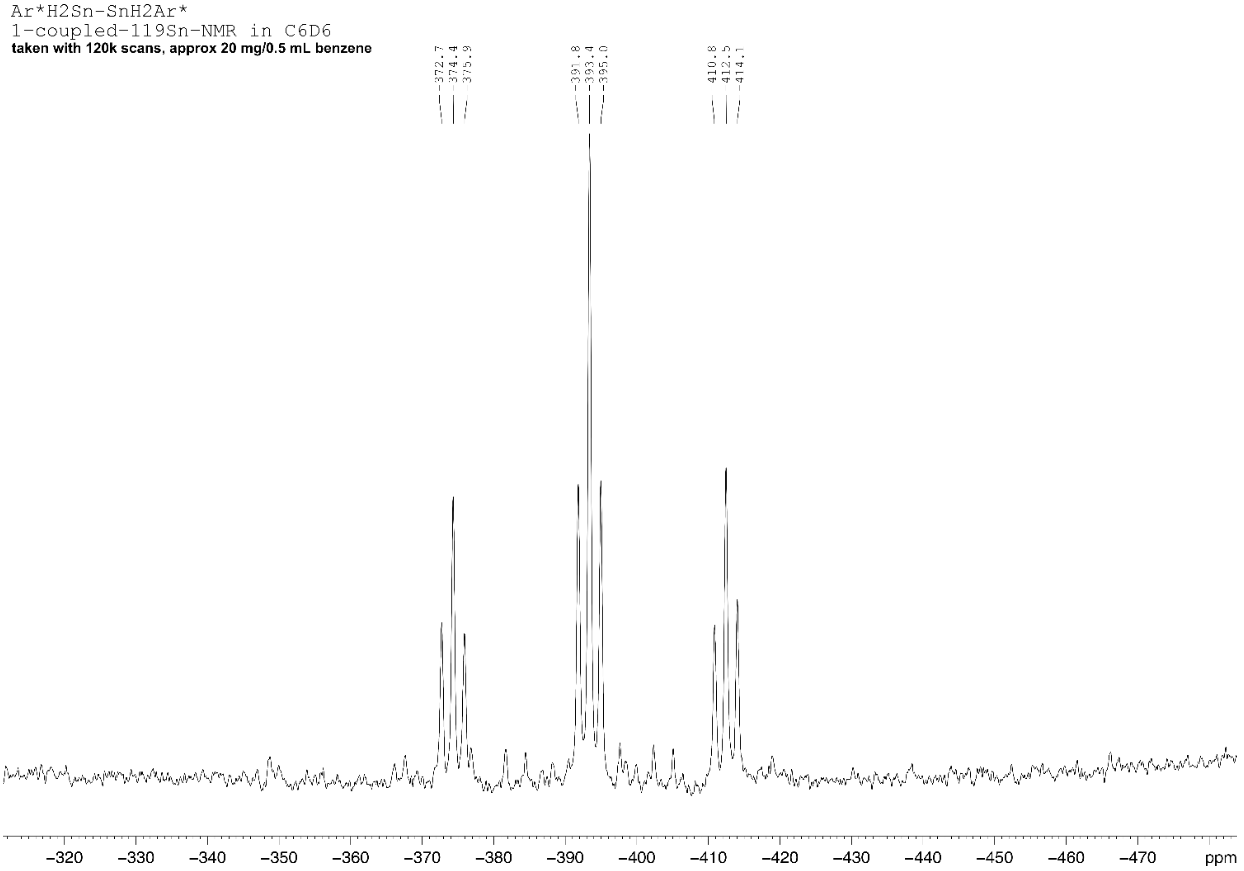

Ar*SnH$_3$ plus DMAP over time

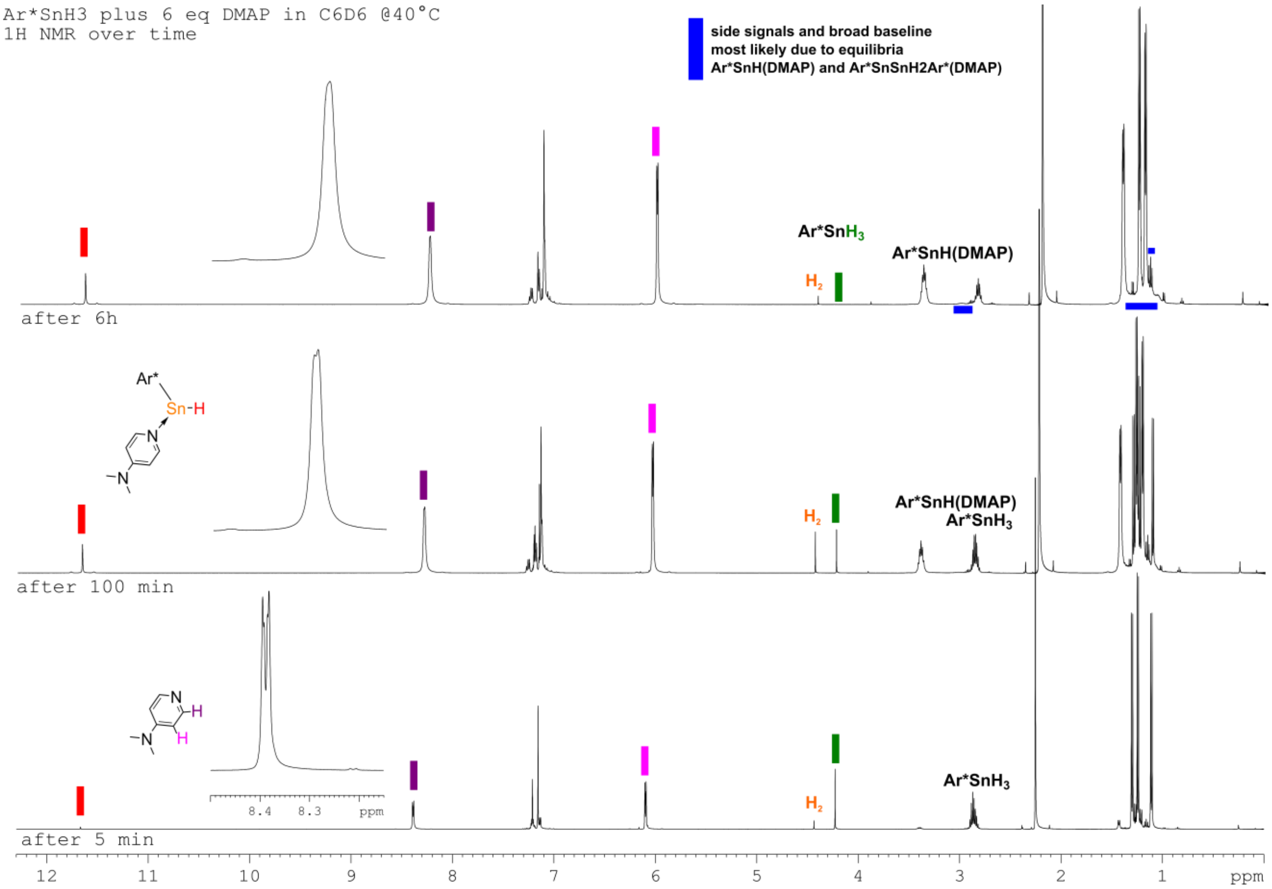

Ar*SnH(DMAP) ^{119}Sn-^{1}H coupled NMR

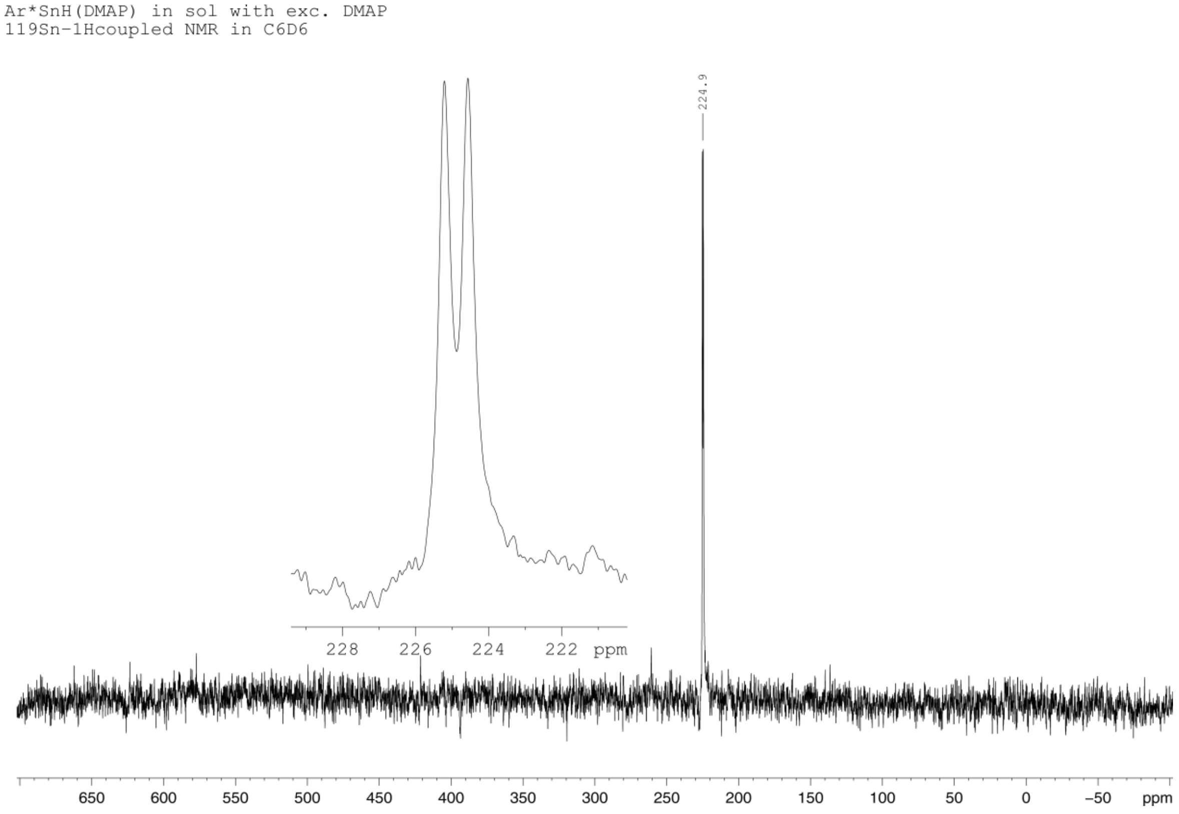

Ar*Sn(DMAP)SnH$_2$Ar* ^{1}H-NMR

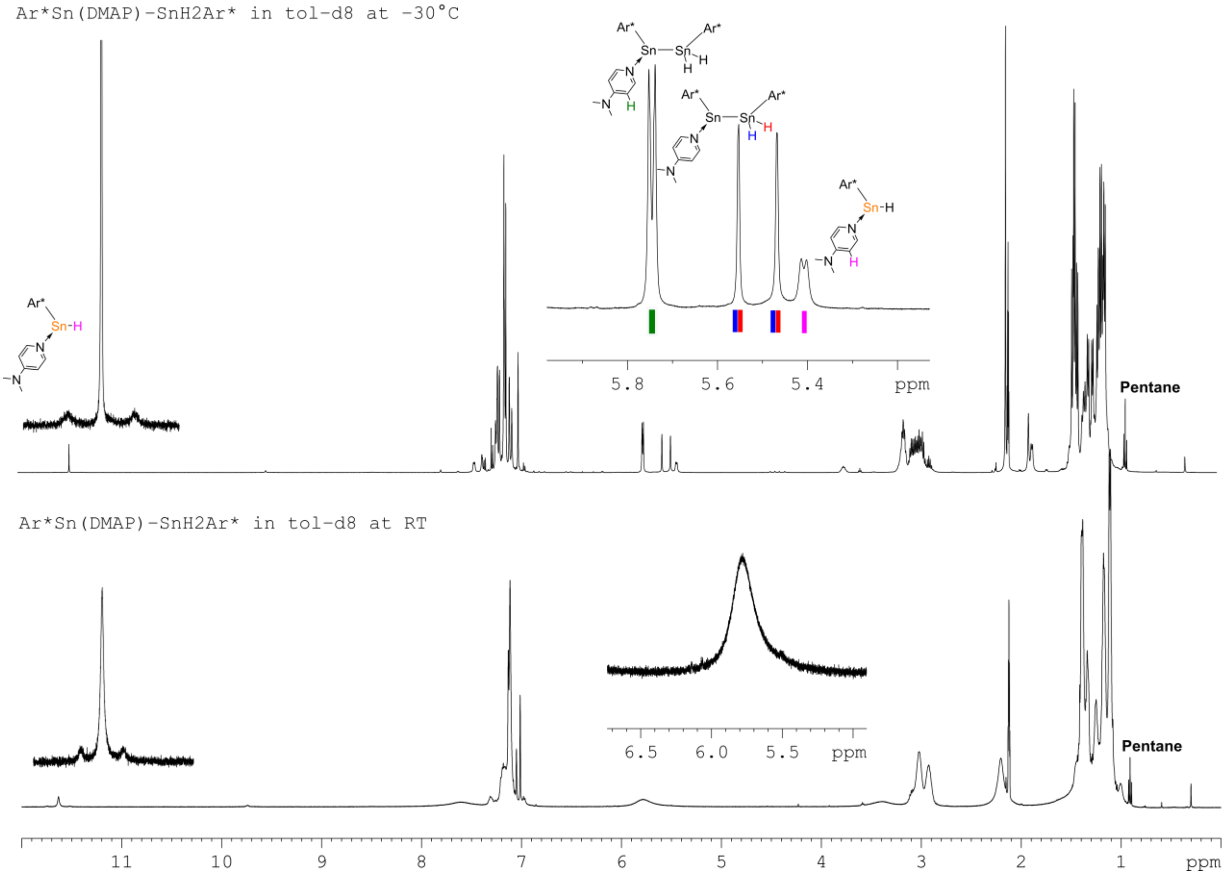

Ar*Sn(DMAP)SnH$_2$Ar* ^{1}H-NMR-details -30°C

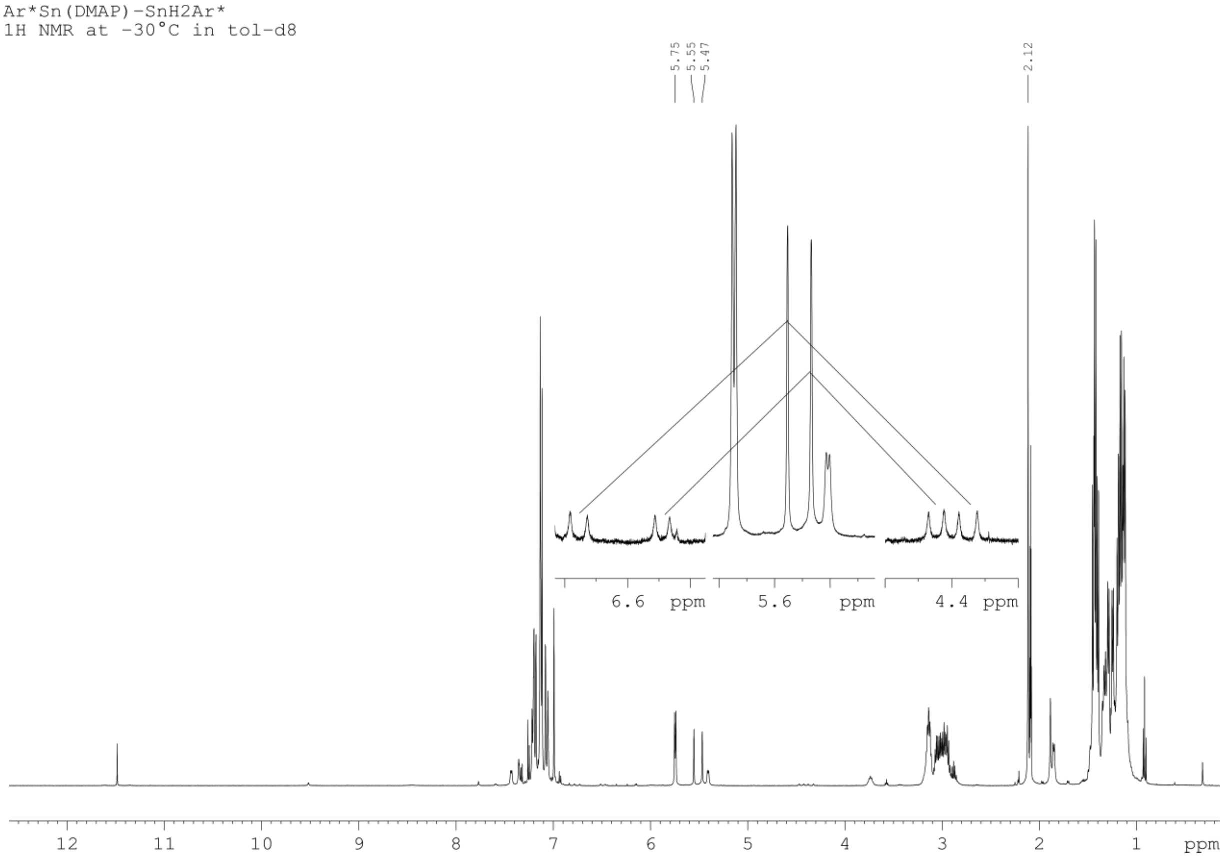

Dissolved crystals of Ar*Sn(DMAP)SnH$_2$Ar* ^{119}Sn -^{1}H-coupled-NMR

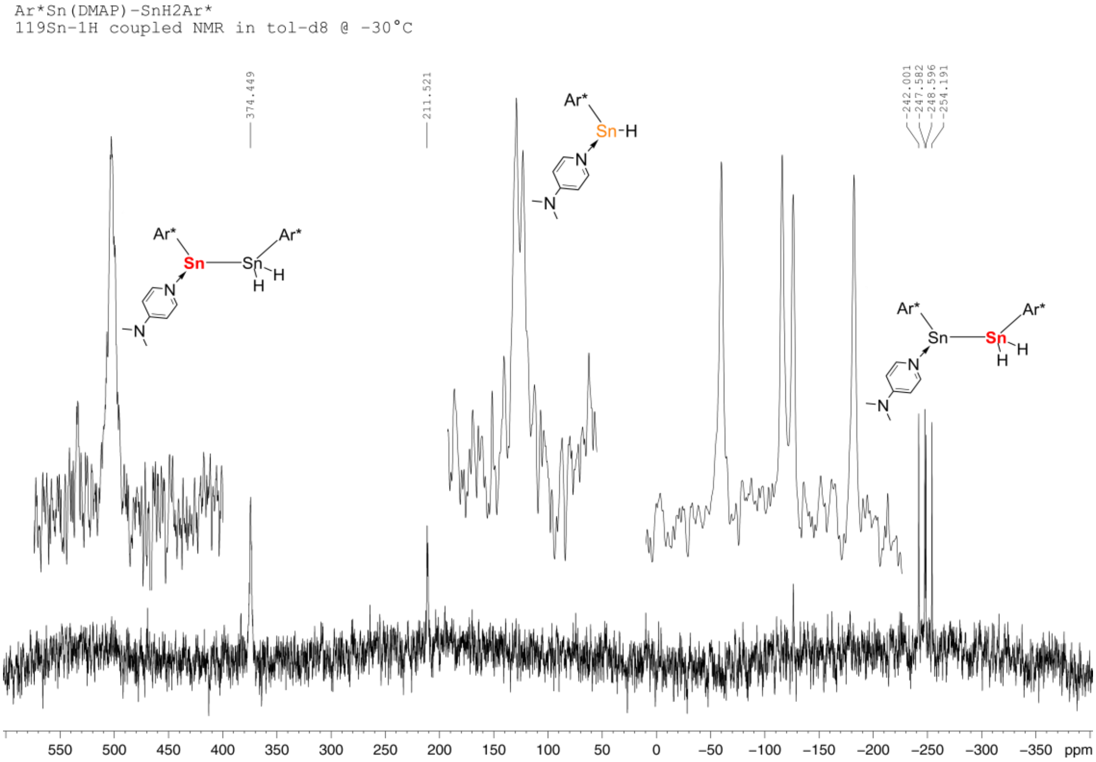

Dissolved crystals of Ar*Sn(DMAP)SnH$_2$Ar* ^{119}Sn-NMR at RT

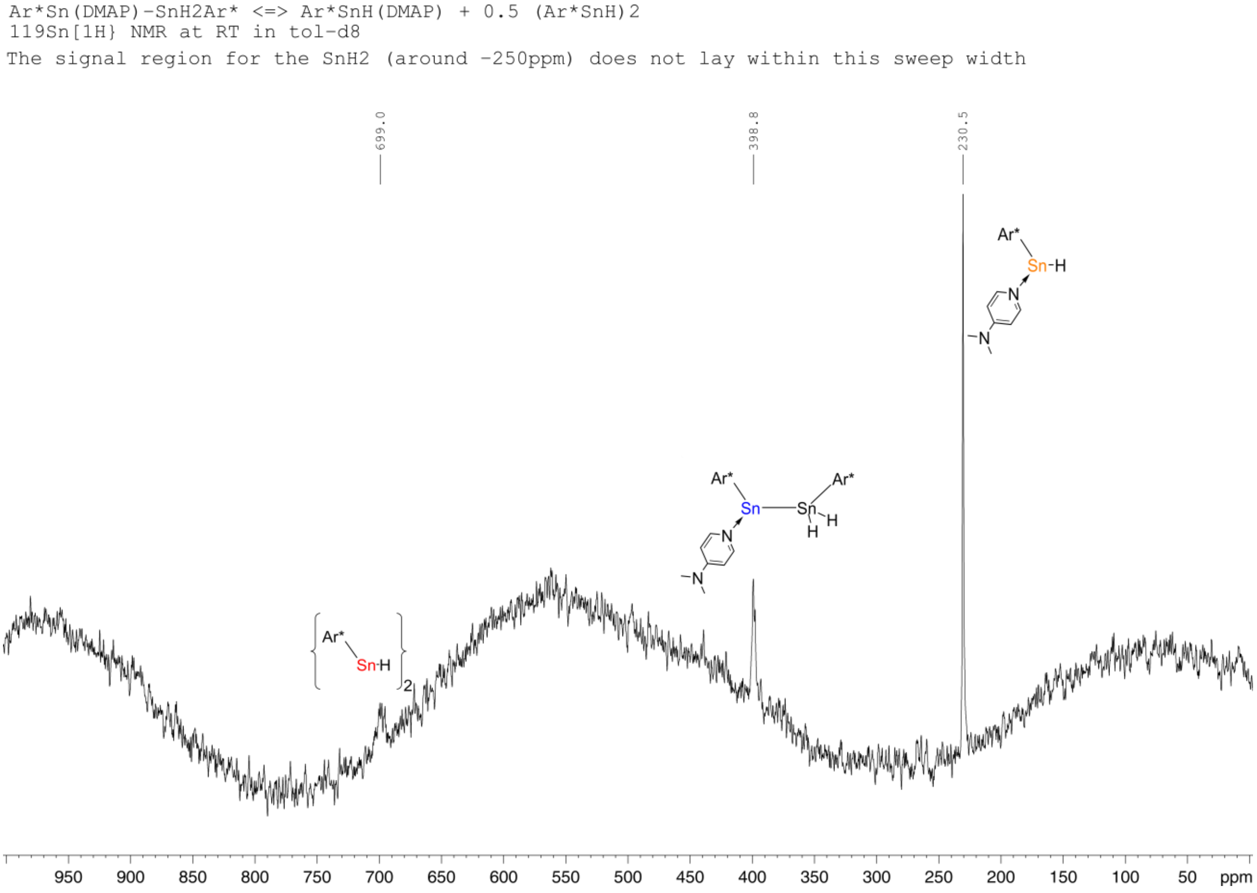

Raw solution ^{1}H-NMR of TMEDA catalysed dehydrogenation

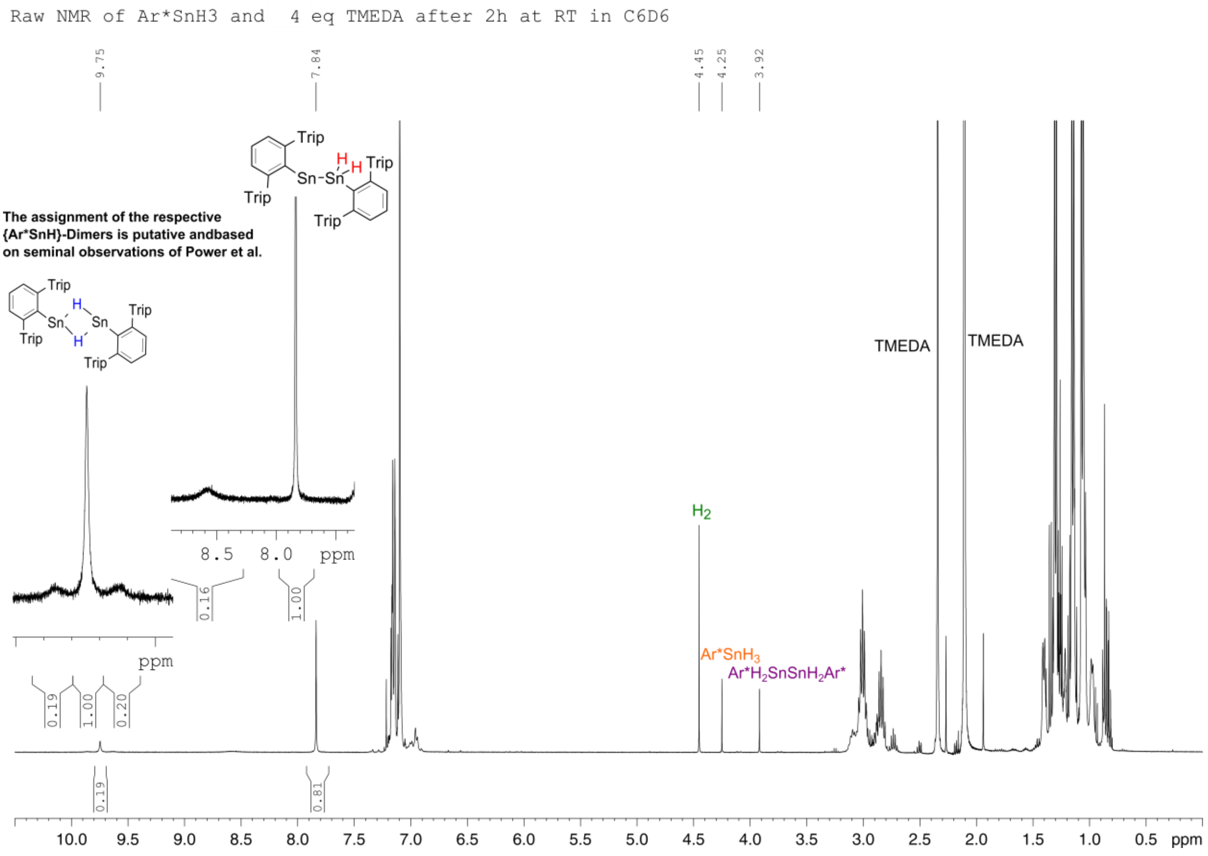

Raw solution ^{1}H-NMR of Et_2NMe catalysed dehydrogenation

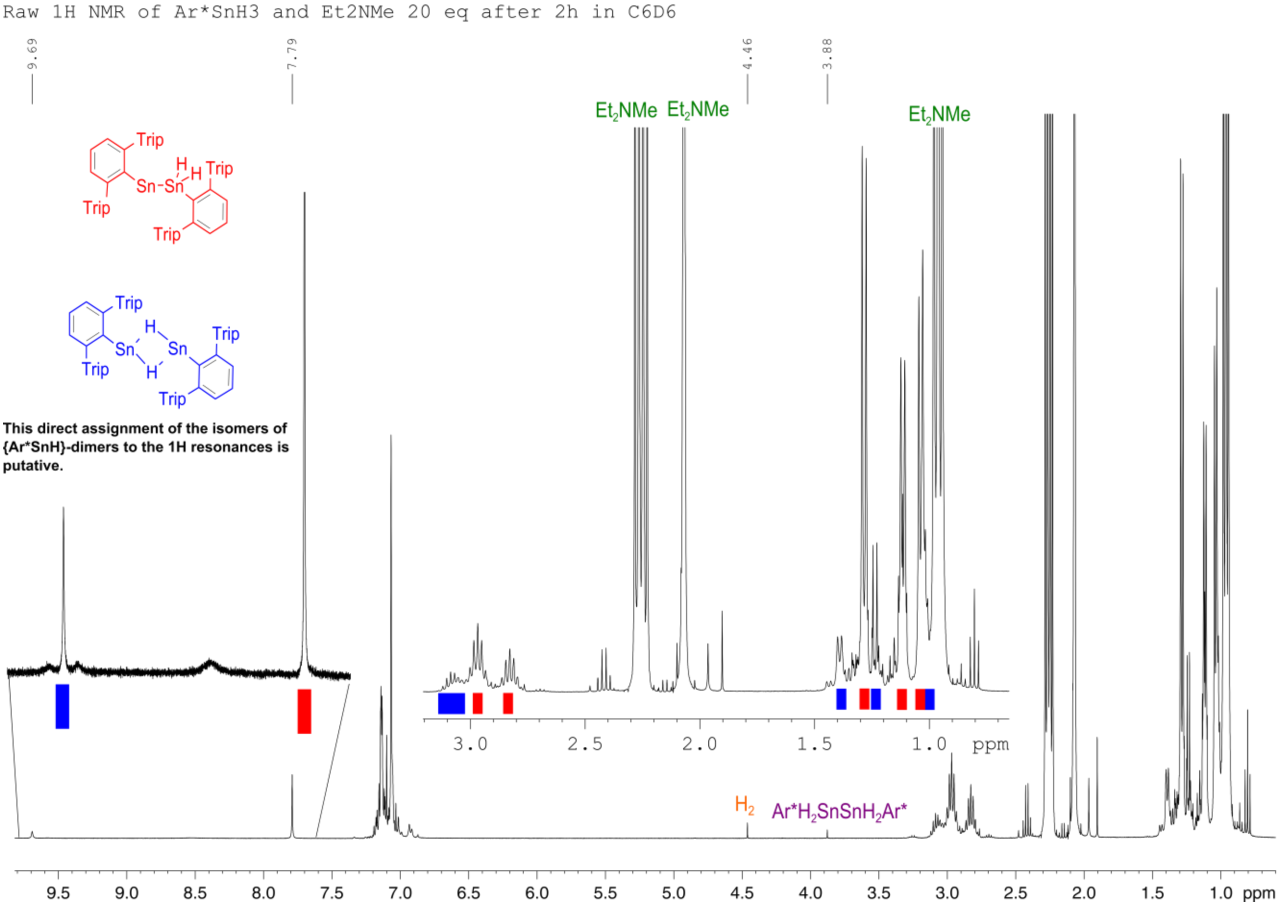

Consecutive addition of DMAP to in situ generated Ar*SnH solutions

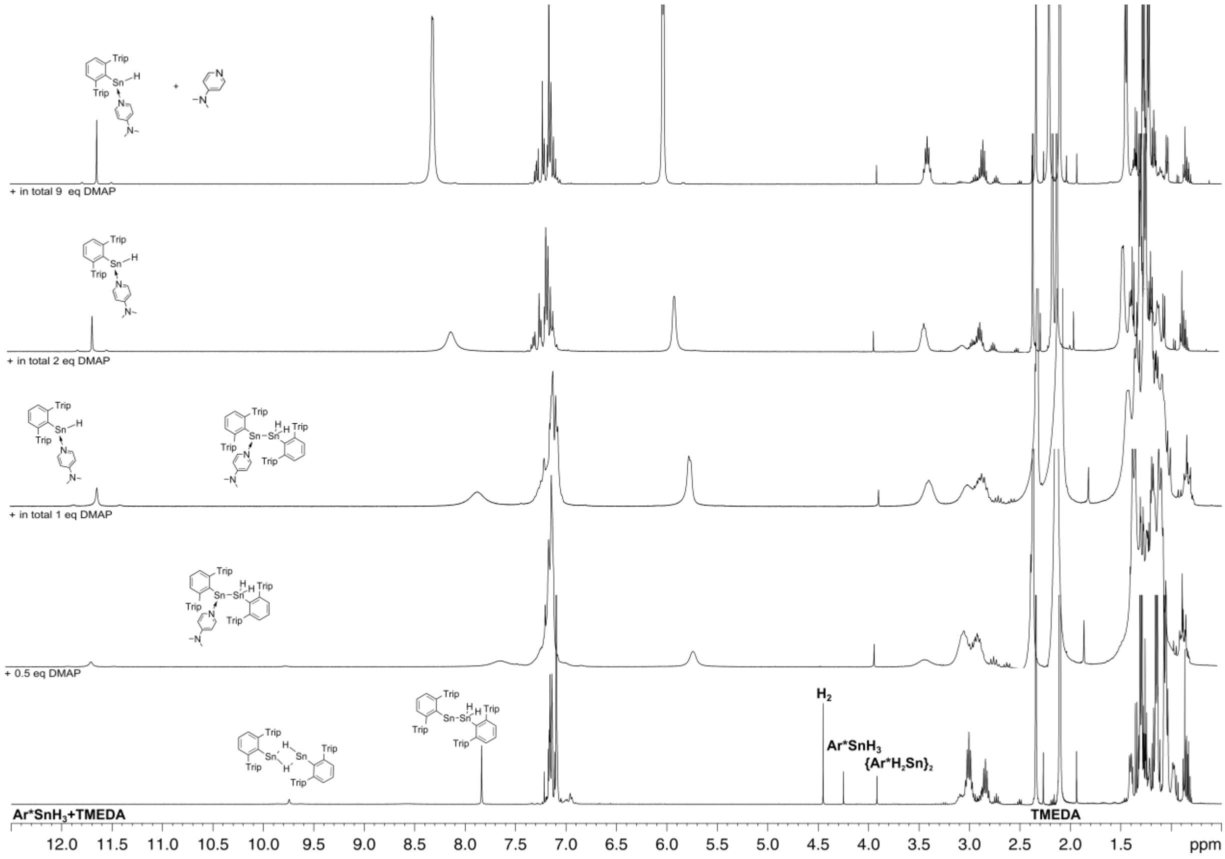

^{119}Sn-NMR of Ar*SnH + 1 eq DMAP

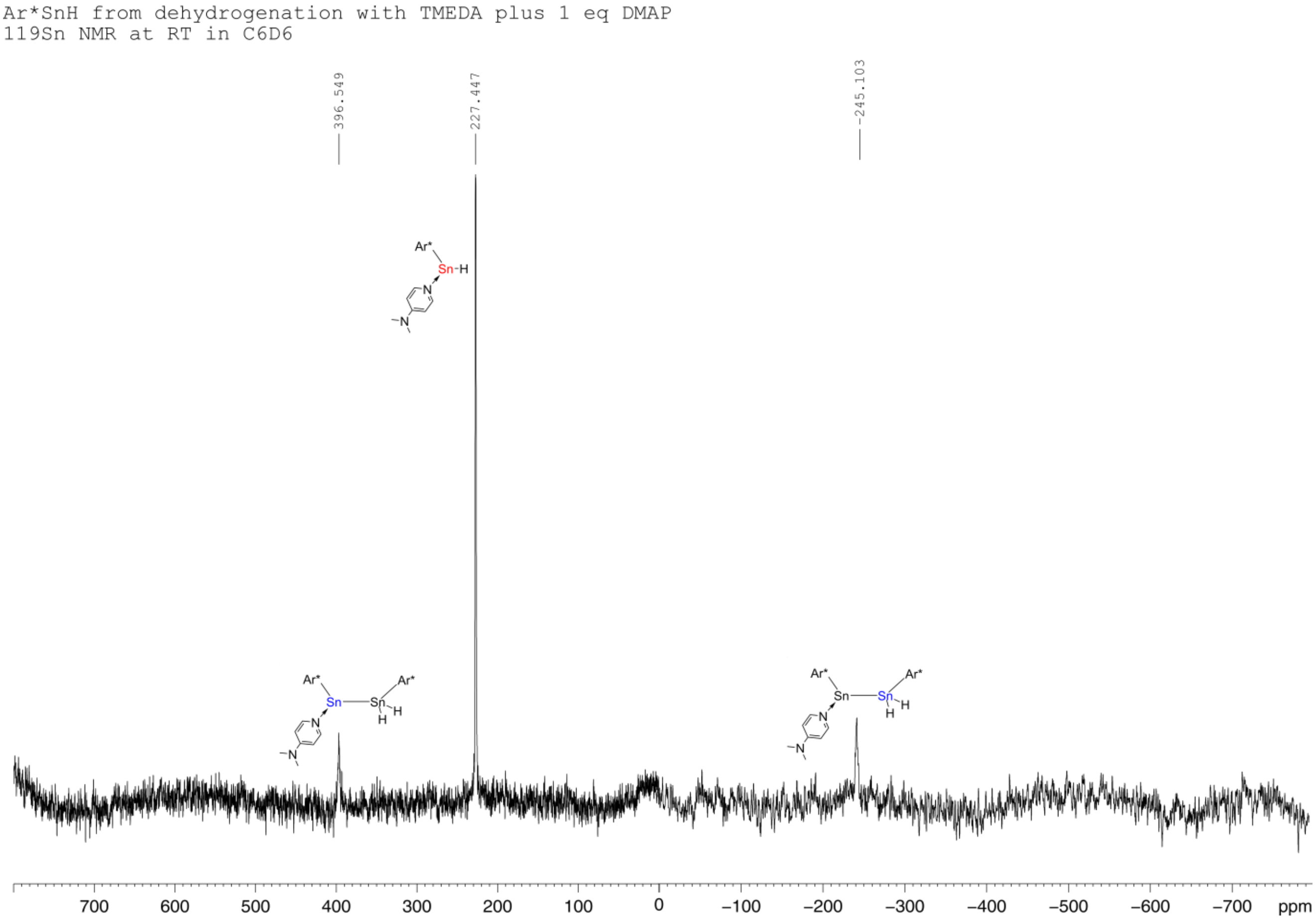

Selected IR Spectra

Ar^*SnH_2-SnH_2Ar^*

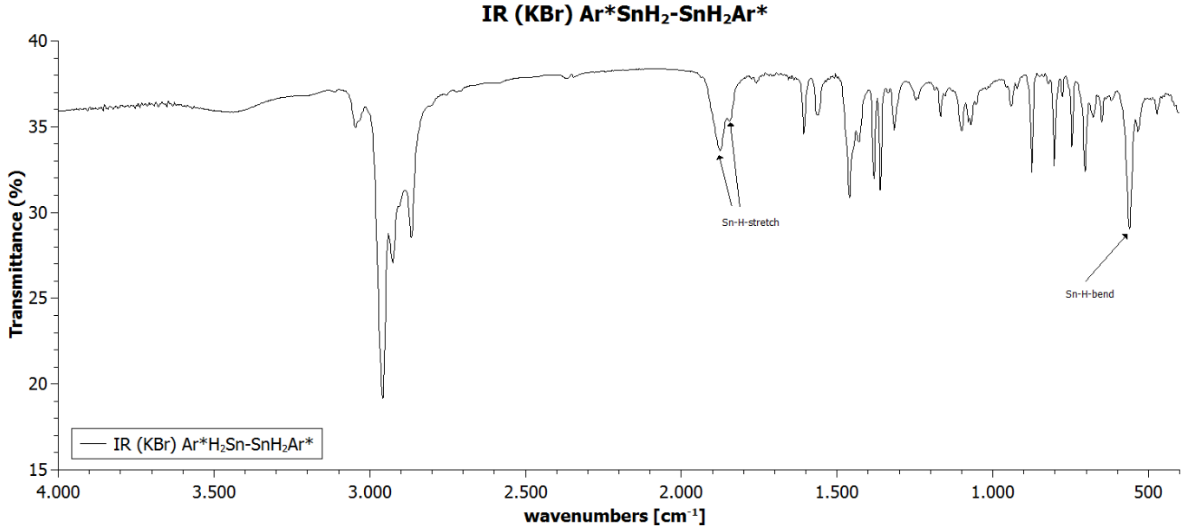

$Ar^*SnH(DMAP)$ / $Ar^*SnH(6DMAP)$ / $Ar^*SnH_2Sn(DMAP)Ar^*$

Depicted are the IR spectra of dried raw solid material of Ar*SnH(ca. 1 DMAP) obtained from the dehydrogenation with 6 equivalent of DMAP and extraction with pentane under removal of the most of the excessive DMAP. The IR spectra of the crude yellow solid obtained after removal of solvents from the reaction of Ar^*SnH_3 and 6 equivalents of DMAP and the IR spectra of isolated crystals of Ar^*SnH_2-Sn(DMAP)Ar*.

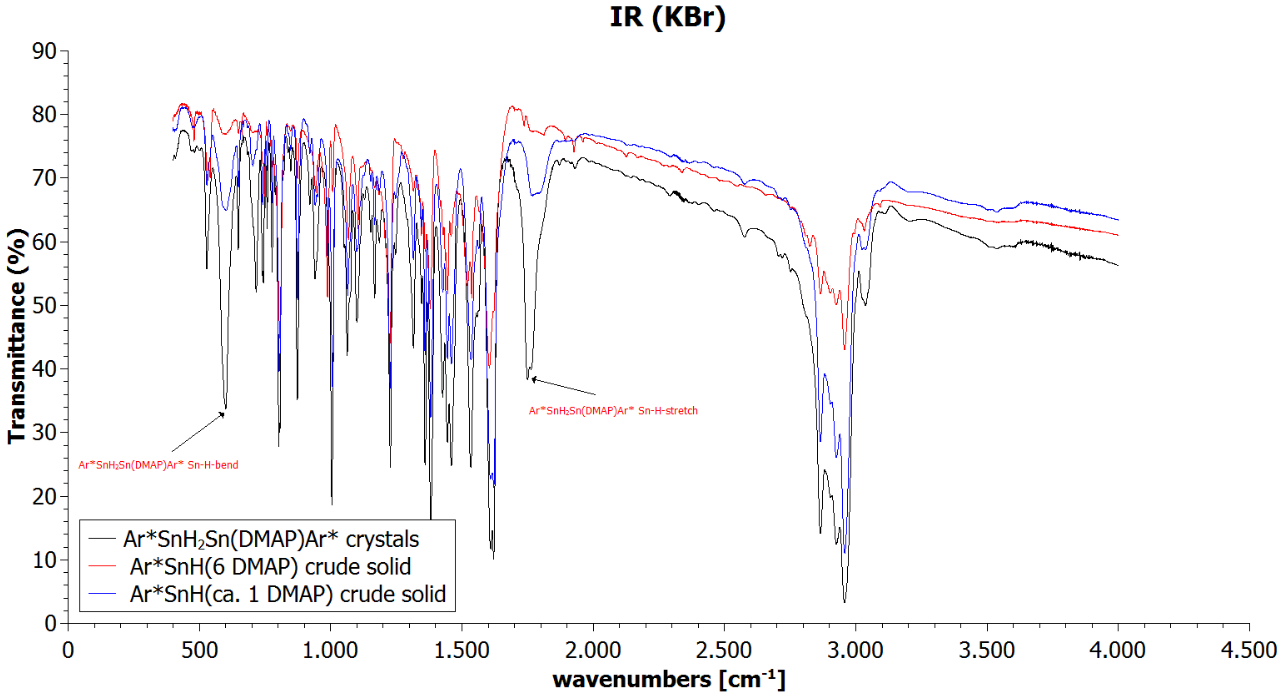

Ar*SnH(DMAP) / Ar*SnD(DMAP)

Depicted are the IR spectra of dried raw solid material of Ar*SnH(ca. 1 DMAP) and Ar*SnD(ca. 1DMAP) obtained from the dehydrogenation with 6 equivalent of DMAP and extraction with pentane under removal of the most of the excessive DMAP.

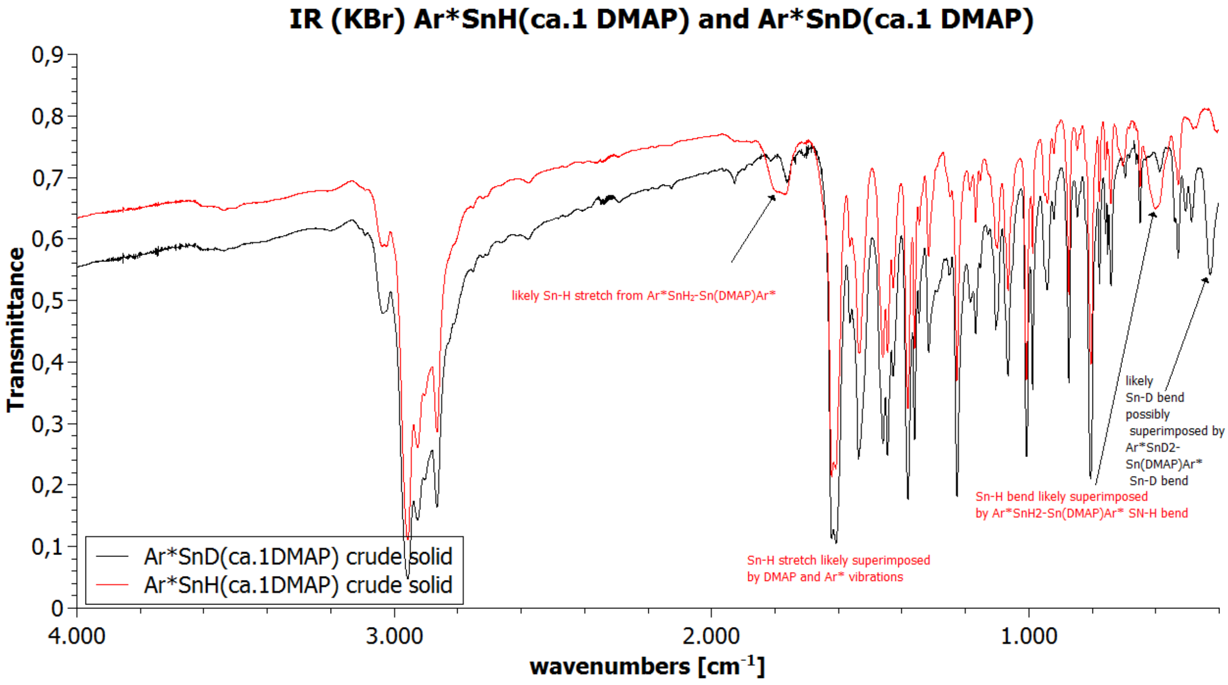

Computational Details

General Methodology and BDE calculations

DFT calculations have been performed using the Gaussian09 Revision D.01 program.[11] For the calculations of bond dissociation energies, the structures have been optimized in the gasphase using the BP86 functional[12,13] with def2TZVP basis set on all atoms (C, H, N, Sn) along with w06 density fitting[14, 15] as well as Stuttgart-Dresden effective core potentials on tin (MWB46) as implemented in Gaussian. Grimme dispersion correction with Becke-Johnson damping has been taken into account using the D3BJ option implemented in Gaussian.[16] Starting geometries for geometry optimizations were directly taken from the X-ray data were available (Ar*SnH_3 and [Ar*SnH]$_2$) or from manipulations on the basis of these X-ray structures (monomeric Ar*SnH). Starting geometries for the optimization of base-adducts to Ar*SnH were obtained from substitution of the donor molecule on the basis of the structure of Ar*SnH(NHC).[17] Thermal corrections were obtained from frequency calculations performed for all optimized structures. Frequency calculations revealed no or a single imaginary frequency smaller than 9 cm^{-1}, except for Ar*SnH were two small imaginary frequencies were obtained (6 and 11 cm^{-1}).

Table 2SI Computationally accessed energies and enthalpies for Ar*SnH_3 and derivatives for the approximation of bond dissociation enthalpies.

Comp.	E/a.u.	E+ZPVE	H	G
Ar*SnH_3	-1407,19433	-1406,44173	-1406,39690	-1406,52121
μ-(Ar*SnH)$_2$	-2812,10217	-2810,62067	-2810,53277	-2810,74554
Ar*SnH	-1406,00603	-1405,26818	-1405,22493	-1405,34586
Ar*SnH(DMAP)	-1788,46949	-1787,57231	-1787,51800	-1787,66410
Ar*SnH(pyridine)	-1654,42926	-1653,60256	-1653,55294	-1653,68827
ArSnH(NMe_3)	-1580,58269	-1579,72442	-1579,67412	-1579,80911
DMAP	-382,42253	-382,26603	-382,25615	-382,30019
Pyridine	-248,38765	-248,30168	-248,29632	-248,32850
NMe_3	-174,54492	-174,42871	-174,42219	-174,45507

The BDEs given in the main text are calculated from

$$BDE = \Delta G(diss) = G(adduct) - G(ArSnH) - G(base)$$

and no further entropy-corrections have been made.

Mechanism Study

For the studies of the mechanism dehydrogenation of model compound $PhSnH_3$ was chosen. Computations were carried out in Gaussian09 Revision D01 using TPSS functional (TPSSTPSS-keyword in Gaussian)[18] along with 6-31G* basis set[19] for C, H and N and a def2TZVP basis set along with Stuttgart-Dresden ECP (MWB46) for Sn. Superfinegrid was applied. Solvent THF was simulated by a polarized continuum model. For **TS-B1** (and **TS-B1(D)**) one strong imaginary frequency at 1228 cm^{-1} (876 cm^{-1} for $PhSnD_3$) was found along with one minor frequency at 6 cm^{-1}. The transition state structures for the NMe_3 and Pyridine deprotonation as well as **TS-A** revealed only one strong imaginary frequency. For **LA(DMAP)** an imaginary frequency was found (14 cm^{-1}). For all other structures frequency calculations revealed no imaginary frequency. Our screenings for putative transition state **TS-B2** remained unsuccessful. The computed energies at standard conditions are given in Table 3SI. E_{rel} and dH, dG values tabulated represent the differences against free $PhSnH_3$ and base.

Table 3SI Computationally accessed energies and enthalpies for the model system $PhSnH_3$ plus DMAP/base in THF (PCM). Relative energies and enthalpies (dH, dG) are given in kcal/mol. Please also note accompanying Scheme 1-SI

Comp.	E/a.u.	E+ZPVE	H	G	E_{rel}	$(E+ZPVE)_{rel}$	dH	dG	-TdS	dGcorr
DMAP	-382,35092	-382,19047	-382,18066	-382,22476						
Pyridine	-248,34295	-248,25510	-248,24980	-248,28190						
NMe_3	-174,51014	-174,39048	-174,38405	-174,41679						
$PhSnH_3$	-236,84686	-236,73888	-236,72991	-236,77739						
**LA(DMAP)**	-619,21653	-618,9461	-618,92787	-618,99466	-11,77	-10,49	-10,85	4,70	15,6	-3,1
**TS-A(DMAP)**	-619,14386	-618,87728	-618,85798	-618,92813	33,83	32,67	33,00	46,45	13,4	39,7
**TS-B1(DMAP)**	-619,18375	-618,91684	-618,89839	-618,96827	8,80	7,85	7,65	21,26	13,6	14,5
$PhSnD_3$	-236,84686	-236,74440	-236,73483	-236,78230						
**TS-B1(^{2}H)(DMAP)**	-619,18375	-618,92165	-618,90259	-618,97365	8,80	8,30	8,10	20,96	12,9	14,5
**LA(pyridine)**	-485,20575	-485,00816	-484,99342	-485,05464	-10,0	-8,9	-8,6	2,9	11,5	-2,8
**TS-A(pyridine)**	-485,13139	-484,93759	-484,92278	-484,98180	36,7	35,4	35,7	48,6	12,9	42,2
**TS-B1(pyridine)**	-485,16999	-484,97550	-484,96067	-485,02412	12,4	11,6	11,95	22,1	10,1	17,0
**TS-B1(^{2}H)(pyridine)**	-485,16999	-484,98037	-484,96494	-485,02966	12,4	12,0	12,35	21,7		
**TS-B1(NMe_3)**	-411,34865	-411,12140	-411,10533	-411,16902	5,2	5,0	5,4	15,8	10,4	10,6

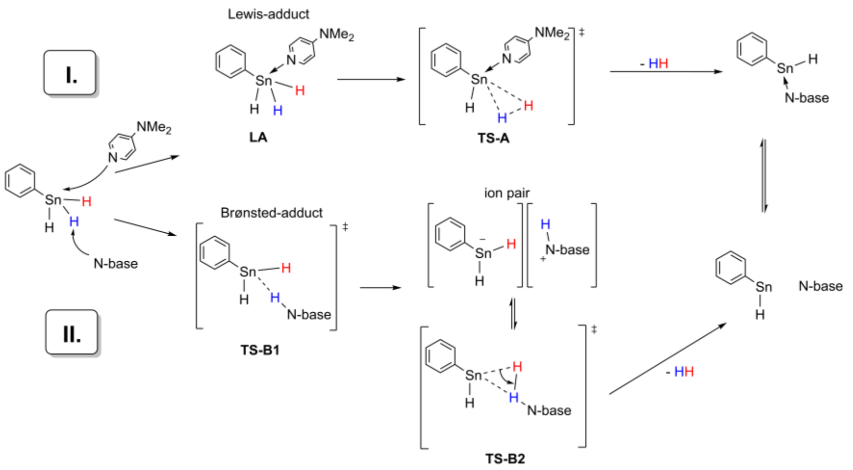

Scheme 1-SI Molecular entities of the $PhSnH_3$ model system that was computationally investigated.

The considerations have to be seen with some caution, since the transition state enthalpies for DMAP may be defective due to a small imaginary frequency for the DMAP transition state.

A rough and only approximating correction of the computed $\Delta G^{‡}_{TS\text{-}B1}$ in terms of a potentially overestimated entropic contribution apply an experimentally approximated scaling factor of 0.5 toward the $T\Delta S^{‡}_{TS\text{-}B1}$ term.[20] This "semiempiric" approach has been applied earlier for similar problems[21] but also critized recently.[22]

$$-T\Delta S = \Delta G - \Delta H$$

$$\Delta G\ (corr) = \Delta H - 0.5T\Delta S$$

Cartesian coordinates of the optimized structure of $PhSnH_3$, DMAP, Pyridine, NMe_3

$PhSnH_3$				DMAP			
Sn	1.569715000	-0.000195000	-0.000009000	N	0.032139000	-2.689550000	0.000000000
C	-0.589311000	-0.014918000	0.000026000	N	-0.084329000	1.558372000	0.000000000
C	-1.323651000	-1.219982000	0.000034000	C	0.018181000	-1.960408000	1.139033000
C	-2.725936000	-1.207425000	0.000035000	H	0.035163000	-2.533500000	2.068368000
C	-3.417549000	0.012734000	0.000027000	C	-0.011258000	-0.569044000	1.204790000
C	-2.703513000	1.218993000	0.000018000	H	-0.012834000	-0.082184000	2.176082000
C	-1.300636000	1.203276000	0.000020000	C	-0.035704000	0.185277000	0.000000000
H	2.189113000	-0.790498000	1.410852000	C	0.018181000	2.288749000	1.263220000
H	2.188505000	-0.822215000	-1.393079000	H	-0.070408000	3.358506000	1.057911000
H	2.087039000	1.650550000	-0.017852000	H	0.980581000	2.104268000	1.766553000
H	-4.506703000	0.022715000	0.000027000	H	-0.793346000	2.000420000	1.945576000
H	-3.236142000	2.169369000	-0.000022000	C	0.018181000	2.288749000	-1.263220000
H	-0.760646000	2.151068000	-0.000318000	H	-0.793346000	2.000420000	-1.945576000
H	-0.806187000	-2.180155000	-0.000207000	H	0.980581000	2.104268000	-1.766553000
H	-3.277157000	-2.147144000	0.000075000	H	-0.070408000	3.358506000	-1.057911000
Pyridine				C	-0.011258000	-0.569044000	-1.204790000
C	0.000000000	-1.148660000	0.723564000	H	-0.012834000	-0.082184000	-2.176082000
N	0.000000000	0.000000000	1.431673000	C	0.018181000	-1.960408000	-1.139033000
C	0.000000000	1.148660000	0.723564000	H	0.035163000	-2.533500000	-2.068368000
C	0.000000000	1.203732000	-0.676373000	NMe_3			
C	0.000000000	0.000000000	-1.392231000	N	0.000000000	0.000000000	0.397816000
C	0.000000000	-1.203732000	-0.676373000	C	0.000000000	1.390368000	-0.065414000
H	0.000000000	0.000000000	-2.480935000	H	-0.891497000	1.903342000	0.318749000
H	0.000000000	2.164491000	-1.187511000	H	0.891497000	1.903342000	0.318749000
H	0.000000000	2.068314000	1.310667000	H	0.000000000	1.477893000	-1.173248000
H	0.000000000	-2.068314000	1.310667000	C	1.204094000	-0.695184000	-0.065414000
H	0.000000000	-2.164491000	-1.187511000	H	2.094091000	-0.179612000	0.318749000
				H	1.202594000	-1.723730000	0.318749000
				H	1.279893000	-0.738947000	-1.173248000
				C	-1.204094000	-0.695184000	-0.065414000
				H	-1.202594000	-1.723730000	0.318749000
				H	-2.094091000	-0.179612000	0.318749000
				H	-1.279893000	-0.738947000	-1.173248000

Cartesian coordinates of the optimized structure of LA, LA(py), TS-A, TS-A(py), TS-B1(DMAP), TS-B1(Py), TS-B1(NMe_3)

Lewis-adduct PhSnH3(DMAP) (LA)				PhSnH3(DMAP)-dehydrogenation (TS-A)			
C	-2.636255000	-0.058455000	1.217698000	C	1.714952000	1.341273000	1.055667000
C	-3.383359000	0.080700000	0.012335000	C	2.373598000	0.494431000	0.142415000
C	-2.697468000	-0.243030000	-1.193813000	C	3.409262000	1.034817000	-0.645615000
C	-1.375097000	-0.660490000	-1.136549000	C	3.783048000	2.380416000	-0.520247000
N	-0.668001000	-0.786471000	0.009920000	C	3.121441000	3.211191000	0.396875000
C	-1.317213000	-0.485196000	1.158237000	C	2.087048000	2.688560000	1.185450000
Sn	1.842995000	-1.540469000	0.015629000	Sn	1.734039000	-1.563091000	-0.137347000
C	2.325997000	0.581932000	-0.022784000	N	-0.615975000	-0.700358000	-0.064608000
C	3.694584000	0.918174000	0.059685000	C	-1.008582000	0.393038000	-0.763572000
C	4.120046000	2.255227000	0.044888000	C	-2.315643000	0.849323000	-0.804379000
C	3.177403000	3.287639000	-0.056607000	C	-3.329432000	0.152025000	-0.083393000
C	1.813428000	2.973510000	-0.142733000	C	-2.906611000	-0.997201000	0.646430000
C	1.393679000	1.634866000	-0.125027000	C	-1.572328000	-1.371269000	0.620172000
H	3.517301000	-2.155033000	0.025489000	H	3.098637000	-2.426541000	-0.851308000
H	1.218493000	-2.245320000	-1.442669000	H	3.728011000	-2.148006000	0.459207000
H	1.231892000	-2.180807000	1.509110000	H	1.308954000	-2.458188000	1.284303000

H	3.502457000	4.327492000	-0.069334000	H	3.410069000	4.257064000	0.494855000
H	5.182488000	2.488917000	0.111492000	H	1.569323000	3.327260000	1.900938000
H	4.440345000	0.124930000	0.135609000	H	0.901772000	0.954146000	1.669985000
H	0.330885000	1.413511000	-0.191786000	H	3.935063000	0.401770000	-1.362140000
H	1.074881000	3.771170000	-0.223593000	H	4.587265000	2.780240000	-1.137563000
N	-4.683909000	0.499341000	0.013449000	N	-4.629930000	0.562689000	-0.090867000
H	-3.188475000	-0.171513000	-2.159510000	H	-3.608407000	-1.588319000	1.226294000
H	-0.837101000	-0.912879000	-2.050379000	H	-1.230853000	-2.245752000	1.173310000
H	-0.733493000	-0.597745000	2.071859000	H	-0.220117000	0.920269000	-1.297787000
H	-3.078450000	0.160988000	2.184672000	H	-2.545040000	1.737725000	-1.384372000
C	-5.351255000	0.816593000	1.278086000	C	-5.637206000	-0.174808000	0.676981000
C	-5.416189000	0.621384000	-1.249137000	C	-5.021062000	1.750811000	-0.854792000
H	-6.429176000	0.968657000	-1.034255000	H	-6.604395000	0.315069000	0.546345000
H	-4.932003000	1.346819000	-1.918647000	H	-5.719228000	-1.212975000	0.325207000
H	-5.479952000	-0.347287000	-1.765618000	H	-5.389354000	-0.182097000	1.747911000
H	-6.374517000	1.132940000	1.064228000	H	-6.096946000	1.900092000	-0.742368000
H	-5.386634000	-0.061490000	1.939066000	H	-4.501564000	2.646917000	-0.486294000
H	-4.835102000	1.632887000	1.803554000	H	-4.794697000	1.623935000	-1.922782000

**Bronsted-Deprotonation TS-B1(DMAP)**				**Bronsted-Deprotonation TS-B1(Pyridin)**			
Sn	-1.871240000	-1.494201000	-0.089367000	Sn	-0.526217000	-1.435173000	-0.133243000
C	-3.115785000	0.331226000	-0.023079000	C	-2.129974000	0.087758000	-0.028982000
C	-4.349170000	0.376657000	0.662772000	C	-3.320383000	-0.121187000	0.701271000
C	-5.114013000	1.552528000	0.718640000	C	-4.315272000	0.866217000	0.780655000
C	-4.658744000	2.717557000	0.083979000	C	-4.140966000	2.093814000	0.125013000
C	-3.435801000	2.698731000	-0.602215000	C	-2.966450000	2.326993000	-0.605165000
C	-2.676546000	1.519095000	-0.647963000	C	-1.975801000	1.335081000	-0.673422000
H	-2.655912000	-2.440482000	1.187416000	H	-1.122055000	-2.562539000	1.101985000
H	0.105471000	-1.101156000	-0.036657000	H	1.381070000	-0.630073000	-0.035244000
H	-2.535485000	-2.330840000	-1.508414000	H	-1.011072000	-2.345043000	-1.583318000
H	-5.250258000	3.631817000	0.125685000	H	-4.911704000	2.861885000	0.184366000
H	-6.062979000	1.560313000	1.255653000	H	-5.224967000	0.678300000	1.351793000
H	-4.722009000	-0.518605000	1.163309000	H	-3.476790000	-1.069668000	1.217874000
H	-1.724065000	1.529814000	-1.183374000	H	-1.066701000	1.542183000	-1.243389000
H	-3.074124000	3.600624000	-1.097011000	H	-2.822321000	3.279176000	-1.116711000
C	1.699210000	0.655485000	0.014886000	C	2.535485000	1.364957000	0.038622000
C	2.496729000	-1.526328000	0.006338000	C	3.725177000	-0.653385000	0.112311000
C	2.979916000	1.181301000	0.043828000	C	3.712829000	2.109389000	0.120800000

H	0.819530000	1.299084000	0.006180000
C	3.814267000	-1.099995000	0.034901000
H	2.243900000	-2.585646000	-0.009498000
C	4.101551000	0.298316000	0.055225000
H	3.110099000	2.258617000	0.057749000
H	4.609192000	-1.838937000	0.041410000
N	1.452132000	-0.671147000	-0.003628000
N	5.381303000	0.766327000	0.084133000
C	5.636320000	2.209991000	0.105103000
H	6.715690000	2.373359000	0.128013000
H	5.226719000	2.695825000	-0.791674000
H	5.191724000	2.675763000	0.995842000
C	6.507910000	-0.171876000	0.094118000
H	6.470666000	-0.822741000	0.978965000
H	6.506927000	-0.798953000	-0.808600000
H	7.437785000	0.399820000	0.120645000

H	1.547005000	1.817583000	-0.025675000
C	4.943209000	0.022830000	0.196588000
H	3.648150000	-1.738917000	0.104200000
C	4.933974000	1.425007000	0.200821000
H	3.672755000	3.195350000	0.122440000
H	5.873439000	-0.535697000	0.257546000
N	2.563562000	0.020442000	0.035571000
H	5.868120000	1.978169000	0.266236000

Bronsted-Deprotonation TS-B1(NMe3)

Sn	0.211851000	-1.223327000	-0.173359000
C	-1.678313000	-0.088731000	-0.055066000
C	-2.809062000	-0.586626000	0.628389000
C	-3.995157000	0.158413000	0.722102000
C	-4.076702000	1.426715000	0.128932000
C	-2.966064000	1.944286000	-0.553448000
C	-1.783005000	1.193896000	-0.636900000
H	-0.137129000	-2.520367000	0.981142000
H	1.842555000	-0.065542000	0.034278000
H	0.006765000	-2.121493000	-1.690327000
H	-4.996188000	2.007191000	0.199731000
H	-4.854063000	-0.249570000	1.255703000
H	-2.766858000	-1.571827000	1.095879000
H	-0.928367000	1.620702000	-1.167024000
H	-3.020963000	2.930322000	-1.015679000
N	2.922005000	0.854665000	0.185543000
C	2.426552000	2.124816000	-0.380223000
H	1.525861000	2.423941000	0.167653000
H	3.184473000	2.917524000	-0.304873000
H	2.168557000	1.962707000	-1.432891000
C	3.182404000	0.943712000	1.635655000
H	3.985945000	1.662114000	1.852407000
H	2.261392000	1.261469000	2.137279000

H	3.468146000	-0.048076000	2.002800000
C	4.064137000	0.301566000	-0.568116000
H	4.318667000	-0.678559000	-0.150054000
H	3.766824000	0.180041000	-1.615629000
H	4.939071000	0.964867000	-0.509192000

**LA(py)**				**TS-A(py)**			
Sn	-0.511013000	-1.692447000	-0.015867000	C	-1.632831000	1.114510000	-0.837715000
H	-1.886840000	-2.806993000	-0.014362000	N	-1.660905000	-0.006002000	-0.084866000
H	0.414675000	-2.168403000	1.366969000	C	-2.787720000	-0.306433000	0.592600000
H	0.244747000	-2.025284000	-1.537936000	C	-3.927716000	0.501094000	0.549335000
C	-2.966747000	2.653769000	0.236485000	C	-3.897004000	1.663149000	-0.232019000
C	-3.464549000	1.582720000	-0.518971000	C	-2.728069000	1.975388000	-0.939057000
C	-2.753202000	0.374459000	-0.573777000	Sn	0.343203000	-1.663266000	-0.048378000
C	-1.528011000	0.217415000	0.107664000	C	1.572754000	0.118482000	0.123036000
C	-1.044758000	1.304646000	0.864089000	C	2.677151000	0.313437000	-0.729506000
C	-1.757775000	2.510702000	0.932777000	C	3.433485000	1.492829000	-0.666713000
H	-3.518422000	3.591931000	0.285004000	C	3.090479000	2.500050000	0.247572000
H	-4.404487000	1.685950000	-1.060577000	C	1.990828000	2.320930000	1.098521000
H	-3.163866000	-0.453453000	-1.154663000	C	1.236837000	1.138990000	1.034351000
H	-0.101156000	1.214901000	1.400823000	H	1.361820000	-2.976936000	-0.641046000
H	-1.369743000	3.337581000	1.527293000	H	1.998406000	-2.779812000	0.668967000
C	4.025211000	1.478193000	-0.255394000	H	-0.386863000	-2.235195000	1.414310000
C	3.915717000	0.474179000	0.714649000	H	3.675589000	3.417602000	0.295330000
C	2.728283000	-0.263045000	0.782515000	H	1.719628000	3.098497000	1.812248000
N	1.684967000	-0.048238000	-0.042379000	H	0.381004000	1.019133000	1.699043000
C	1.796924000	0.921070000	-0.973993000	H	2.955824000	-0.459045000	-1.447715000
C	2.944467000	1.706154000	-1.117950000	H	4.286012000	1.626532000	-1.332112000
H	4.933618000	2.071775000	-0.337687000	H	-4.767041000	2.313983000	-0.288358000
H	4.729096000	0.264189000	1.405354000	H	-4.813191000	0.223035000	1.115548000
H	2.592337000	-1.055699000	1.518161000	H	-2.755963000	-1.220379000	1.184492000
H	0.926097000	1.065876000	-1.612035000	H	-0.696376000	1.314724000	-1.354900000
H	2.986468000	2.476096000	-1.884903000	H	-2.661845000	2.868637000	-1.555294000

Cartesian coordinates of the optimized structures of Ar*SnH_3, Ar*SnH, μ-bridged (Ar*SnH)$_2$, Ar*SnH(DMAP), Ar*SnH(py), Ar*SnH(NMe_3)

Ar*SnH3				Ar*SnH			
C	0.025940000	0.413055000	0.791450000	Sn	0.023014000	-0.205398000	-1.199413000
C	-1.204935000	0.645143000	1.436131000	C	0.021278000	0.300376000	0.985953000
C	-1.220518000	1.140503000	2.748092000	C	-1.202194000	0.375152000	1.666995000
C	-0.024222000	1.391416000	3.422819000	C	-1.220062000	0.634526000	3.043056000
C	1.197391000	1.142447000	2.794094000	C	-0.012887000	0.822733000	3.728408000
C	1.232097000	0.653079000	1.480233000	C	1.210399000	0.749002000	3.052078000
C	2.517203000	0.365094000	0.780477000	C	1.228533000	0.481691000	1.675792000
C	2.948982000	-0.977139000	0.648956000	C	2.453250000	0.354684000	0.828514000
C	4.072513000	-1.247378000	-0.137349000	C	3.188084000	-0.854294000	0.815845000
C	4.774360000	-0.230528000	-0.795069000	C	4.205372000	-1.018279000	-0.130492000
C	4.343048000	1.087749000	-0.628363000	C	4.510472000	-0.028228000	-1.070542000
C	3.227198000	1.410755000	0.154009000	C	3.785555000	1.166391000	-1.032725000
C	2.743260000	2.851958000	0.258469000	C	2.764809000	1.388323000	-0.096313000
C	3.886267000	3.875619000	0.283394000	C	2.079214000	2.745368000	0.010991000
H	3.488987000	4.878797000	0.497958000	C	2.751350000	3.567824000	1.127557000

H	4.630374000	3.627867000	1.054365000	H	2.248457000	4.539522000	1.247257000
H	4.407121000	3.933021000	-0.684499000	H	2.707661000	3.039112000	2.089814000
C	1.744652000	3.175785000	-0.868053000	H	3.809674000	3.754670000	0.887128000
H	1.408955000	4.221940000	-0.801436000	C	2.039844000	3.534830000	-1.300613000
H	2.204632000	3.020070000	-1.855468000	H	1.437381000	4.445081000	-1.167453000
H	0.854128000	2.534440000	-0.803636000	H	3.043625000	3.852028000	-1.623485000
H	2.196803000	2.943540000	1.210197000	H	1.580718000	2.944863000	-2.106725000
H	4.892447000	1.883601000	-1.136591000	H	1.037296000	2.561528000	0.316138000
C	5.968748000	-0.549915000	-1.676310000	H	4.030806000	1.949626000	-1.752910000
C	7.102902000	-1.214671000	-0.878367000	C	5.586243000	-0.248012000	-2.119168000
H	7.980783000	-1.388804000	-1.519044000	C	6.959898000	-0.513300000	-1.481686000
H	7.412030000	-0.586609000	-0.030416000	H	7.735285000	-0.615276000	-2.256008000
H	6.783291000	-2.188340000	-0.476289000	H	7.249254000	0.305700000	-0.807565000
C	5.556069000	-1.414581000	-2.879862000	H	6.949667000	-1.444087000	-0.894102000
H	4.759376000	-0.928339000	-3.460812000	C	5.193872000	-1.379649000	-3.085020000
H	6.414073000	-1.592954000	-3.545909000	H	4.221283000	-1.178326000	-3.556746000
H	5.177943000	-2.393696000	-2.547948000	H	5.947359000	-1.495188000	-3.878948000
H	6.347716000	0.411223000	-2.064800000	H	5.115552000	-2.339869000	-2.552306000
H	4.405248000	-2.280857000	-0.249019000	H	5.659389000	0.684790000	-2.704538000
C	2.231476000	-2.096198000	1.394256000	H	4.761240000	-1.958407000	-0.149130000
C	2.786663000	-2.211232000	2.826253000	C	2.824324000	-1.997073000	1.753327000
H	2.677012000	-1.262619000	3.370030000	C	4.052558000	-2.678729000	2.372102000
H	2.254474000	-2.993406000	3.389077000	H	4.706367000	-1.947841000	2.869836000
H	3.856809000	-2.469354000	2.803587000	H	3.737123000	-3.422904000	3.118549000
C	2.278251000	-3.450829000	0.678304000	H	4.652105000	-3.208788000	1.616414000
H	1.634533000	-4.173049000	1.201502000	C	1.929415000	-3.015683000	1.025286000
H	1.923164000	-3.368347000	-0.358781000	H	1.627610000	-3.827729000	1.704345000
H	3.294857000	-3.872737000	0.662777000	H	1.018559000	-2.534683000	0.640134000
H	1.171386000	-1.807412000	1.481382000	H	2.459001000	-3.459733000	0.168221000
H	2.136659000	1.325730000	3.320338000	H	2.229890000	-1.570876000	2.575105000
H	-0.043932000	1.775263000	4.444558000	H	2.147088000	0.896899000	3.595313000
H	-2.179329000	1.327476000	3.236018000	H	-0.026196000	1.028627000	4.800355000
C	-2.471189000	0.349102000	0.704023000	H	-2.169948000	0.690855000	3.579891000
C	-2.933606000	-0.987798000	0.621145000	C	-2.414325000	0.164607000	0.815930000
C	-4.071432000	-1.257985000	-0.143964000	C	-2.822892000	-1.156319000	0.492217000
C	-4.764778000	-0.247014000	-0.822261000	C	-3.893464000	-1.335630000	-0.392068000
C	-4.291962000	1.062053000	-0.718930000	C	-4.563040000	-0.250826000	-0.968734000
C	-3.150508000	1.381726000	0.027885000	C	-4.130928000	1.040388000	-0.651694000
C	-2.650444000	2.818179000	0.068925000	C	-3.060239000	1.273753000	0.219729000
C	-2.383054000	3.371544000	-1.340014000	C	-2.609259000	2.690698000	0.537451000
H	-1.691236000	2.721420000	-1.894196000	C	-2.595168000	3.608678000	-0.691756000
H	-3.311638000	3.447797000	-1.926072000	H	-1.993036000	3.164484000	-1.497471000
H	-1.940846000	4.377444000	-1.280577000	H	-3.608931000	3.801117000	-1.075371000
C	-3.622286000	3.718764000	0.849197000	H	-2.155306000	4.582436000	-0.429132000
H	-3.769592000	3.346403000	1.873488000	C	-3.469185000	3.283987000	1.668064000
H	-3.237652000	4.748461000	0.908301000	H	-3.423011000	2.659412000	2.571463000
H	-4.608105000	3.752460000	0.359955000	H	-3.122170000	4.294943000	1.931095000

H	-1.691882000	2.818577000	0.609502000	H	-4.523669000	3.353039000	1.358087000
H	-4.822147000	1.854901000	-1.253942000	H	-1.574126000	2.619964000	0.906904000
C	-5.987292000	-0.567796000	-1.663601000	H	-4.637124000	1.889495000	-1.117252000
C	-5.625286000	-1.481962000	-2.846666000	C	-5.708153000	-0.466619000	-1.942359000
H	-4.834257000	-1.033632000	-3.464715000	C	-5.218239000	-1.152332000	-3.229604000
H	-5.259857000	-2.457565000	-2.491003000	H	-4.411222000	-0.574717000	-3.702999000
H	-6.504268000	-1.663517000	-3.483879000	H	-4.828002000	-2.158705000	-3.013113000
C	-7.116004000	-1.178107000	-0.815991000	H	-6.040091000	-1.257810000	-3.954131000
H	-8.013647000	-1.352249000	-1.428674000	C	-6.863660000	-1.251794000	-1.300393000
H	-6.809112000	-2.145247000	-0.389033000	H	-7.706774000	-1.346592000	-2.001407000
H	-7.388816000	-0.514881000	0.017523000	H	-6.544539000	-2.267931000	-1.022245000
H	-6.353642000	0.388479000	-2.075522000	H	-7.224861000	-0.752374000	-0.389865000
H	-4.427506000	-2.287137000	-0.217815000	H	-6.089337000	0.532288000	-2.216181000
C	-2.233192000	-2.097404000	1.395526000	H	-4.209310000	-2.351027000	-0.639963000
C	-2.303852000	-3.469020000	0.714745000	C	-2.154512000	-2.353805000	1.152843000
H	-1.670824000	-4.188298000	1.254905000	C	-1.959986000	-3.543732000	0.205041000
H	-3.327284000	-3.874447000	0.712577000	H	-1.373016000	-4.330404000	0.701314000
H	-1.950286000	-3.417166000	-0.324767000	H	-2.918628000	-3.991877000	-0.097650000
C	-2.787174000	-2.168758000	2.830820000	H	-1.422262000	-3.241118000	-0.706477000
H	-2.662461000	-1.209533000	3.351950000	C	-2.939552000	-2.763180000	2.412261000
H	-3.860965000	-2.411818000	2.815908000	H	-3.013525000	-1.924330000	3.118906000
H	-2.265611000	-2.945084000	3.411442000	H	-3.961753000	-3.075871000	2.147991000
H	-1.168426000	-1.822405000	1.471286000	H	-2.443999000	-3.601995000	2.924464000
Sn	0.051232000	-0.378234000	-1.248730000	H	-1.156417000	-2.026795000	1.483851000
H	-0.112259000	-2.108298000	-1.177824000	H	-0.366212000	1.531478000	-1.646421000
H	-1.267807000	0.271180000	-2.171146000				
H	1.527281000	0.005455000	-2.074719000				

Hydride-bridged (ArSnH)2				Ar*SnH(DMAP)			
Sn	-0.000001000	-1.337839000	0.942568000	N	-1.318599000	1.527844000	-0.853834000
C	0.000000000	-2.664265000	-0.886351000	N	-4.809323000	3.860045000	-0.842208000
C	-1.211900000	-3.175820000	-1.393832000	Sn	0.719937000	0.311385000	-1.153336000
C	-1.204776000	-4.148988000	-2.406354000	C	-2.507121000	1.015344000	-1.235540000
C	0.000000000	-4.630663000	-2.917963000	H	-2.508177000	-0.037317000	-1.523540000
C	1.204775000	-4.148989000	-2.406354000	C	-3.679550000	1.746573000	-1.252160000
C	1.211900000	-3.175820000	-1.393832000	H	-4.599141000	1.245224000	-1.543604000
C	2.535165000	-2.772649000	-0.824610000	C	-3.668630000	3.108798000	-0.862161000
C	3.082459000	-3.514566000	0.246902000	C	-6.087901000	3.245312000	-1.168847000
C	4.388749000	-3.235837000	0.669470000	H	-6.875920000	4.002688000	-1.100458000
C	5.156458000	-2.232530000	0.071967000	H	-6.334976000	2.423309000	-0.475040000
C	4.569202000	-1.467067000	-0.941524000	H	-6.088666000	2.841788000	-2.194622000
C	3.274445000	-1.718201000	-1.407354000	C	-4.767718000	5.237820000	-0.374585000
C	-2.535166000	-2.772649000	-0.824609000	H	-4.076022000	5.845133000	-0.980711000
C	-3.082460000	-3.514565000	0.246903000	H	-4.451995000	5.303278000	0.681092000
C	-4.388750000	-3.235836000	0.669470000	H	-5.768128000	5.674678000	-0.462365000
C	-5.156458000	-2.232530000	0.071967000	C	-2.410632000	3.640333000	-0.476202000
C	-4.569202000	-1.467066000	-0.941524000	H	-2.304805000	4.672633000	-0.149515000

C	-3.274445000	-1.718201000	-1.407354000	C	-1.291239000	2.826776000	-0.482862000
C	-2.690511000	-0.910482000	-2.555274000	H	-0.312082000	3.200201000	-0.178120000
C	-3.119889000	0.558542000	-2.530123000	C	0.764033000	-0.653567000	0.918575000
C	-3.039645000	-1.545246000	-3.913671000	C	-0.286507000	-1.367700000	1.532951000
C	-6.610702000	-2.008429000	0.449904000	C	-1.637793000	-1.471463000	0.900766000
C	-6.840610000	-1.880537000	1.961793000	C	-2.744667000	-0.792921000	1.453875000
C	-7.489142000	-3.125137000	-0.144109000	C	-2.597652000	0.096495000	2.680133000
C	-2.295203000	-4.634506000	0.914179000	H	-1.520413000	0.214670000	2.870012000
C	-2.169731000	-4.426600000	2.431145000	C	-3.186534000	1.498528000	2.468837000
C	-2.903455000	-6.007175000	0.583113000	H	-3.034689000	2.113854000	3.368969000
C	2.295203000	-4.634506000	0.914178000	H	-4.269242000	1.461087000	2.271481000
C	2.903456000	-6.007176000	0.583114000	H	-2.706075000	2.007024000	1.624121000
C	2.169728000	-4.426599000	2.431144000	C	-3.223511000	-0.575559000	3.915008000
C	6.610701000	-2.008429000	0.449905000	H	-3.076797000	0.044921000	4.812461000
C	7.489142000	-3.125136000	-0.144110000	H	-2.774851000	-1.561049000	4.102441000
C	6.840609000	-1.880539000	1.961794000	H	-4.306200000	-0.721514000	3.774585000
C	2.690511000	-0.910482000	-2.555274000	C	-4.009411000	-0.964228000	0.874045000
C	3.039644000	-1.545246000	-3.913671000	H	-4.868331000	-0.435973000	1.298092000
C	3.119890000	0.558542000	-2.530123000	C	-4.210400000	-1.780142000	-0.239856000
H	-1.210230000	0.000000000	0.000000000	C	-5.597679000	-1.957803000	-0.832209000
H	-2.155286000	-4.544147000	-2.771779000	H	-6.258227000	-1.232832000	-0.324063000
H	-0.000001000	-5.394890000	-3.697493000	C	-6.141032000	-3.368703000	-0.544460000
H	2.155285000	-4.544148000	-2.771779000	H	-6.147450000	-3.575450000	0.535230000
H	4.817577000	-3.827704000	1.481822000	H	-5.511627000	-4.131678000	-1.027741000
H	5.150493000	-0.657204000	-1.386302000	H	-7.167057000	-3.482785000	-0.927157000
H	-4.817578000	-3.827703000	1.481823000	C	-5.634314000	-1.655865000	-2.338896000
H	-5.150494000	-0.657204000	-1.386302000	H	-6.661092000	-1.731591000	-2.727931000
H	-1.593023000	-0.947100000	-2.445380000	H	-5.011778000	-2.367381000	-2.901752000
H	-3.001312000	1.002252000	-1.532045000	H	-5.257856000	-0.645618000	-2.557919000
H	-4.175248000	0.680545000	-2.813718000	C	-3.100358000	-2.445388000	-0.776333000
H	-2.523941000	1.142999000	-3.246253000	H	-3.237036000	-3.098540000	-1.640514000
H	-2.657027000	-2.571564000	-3.988091000	C	-1.819725000	-2.312890000	-0.227217000
H	-2.603821000	-0.957074000	-4.735951000	C	-0.667027000	-3.145144000	-0.773396000
H	-4.131617000	-1.573990000	-4.053315000	H	0.263195000	-2.609640000	-0.533684000
H	-6.918058000	-1.058244000	-0.020200000	C	-0.703632000	-3.331638000	-2.293744000
H	-6.255323000	-1.054778000	2.390424000	H	-1.558415000	-3.945515000	-2.618017000
H	-6.554772000	-2.801961000	2.491075000	H	0.210826000	-3.842365000	-2.629196000
H	-7.903215000	-1.691090000	2.175679000	H	-0.752604000	-2.360335000	-2.807677000
H	-8.554391000	-2.940366000	0.063728000	C	-0.610379000	-4.499515000	-0.043437000
H	-7.220559000	-4.100342000	0.290699000	H	-0.502045000	-4.356153000	1.040914000
H	-7.353968000	-3.195350000	-1.232911000	H	0.245555000	-5.094083000	-0.398090000
H	-1.277984000	-4.617667000	0.494205000	H	-1.530304000	-5.077793000	-0.223563000
H	-1.560192000	-5.225192000	2.880194000	C	-0.068628000	-2.062330000	2.736349000
H	-3.152777000	-4.434506000	2.925910000	H	-0.895264000	-2.615591000	3.188710000
H	-1.688742000	-3.463228000	2.659515000	C	1.187519000	-2.068032000	3.342049000
H	-2.937411000	-6.167566000	-0.504115000	H	1.344814000	-2.609746000	4.276734000
H	-3.931779000	-6.087014000	0.968357000	C	2.248416000	-1.404552000	2.723132000

H	-2.307845000	-6.816452000	1.032494000	H	3.249451000	-1.436267000	3.159992000
H	1.277984000	-4.617669000	0.494203000	C	2.043601000	-0.718630000	1.518027000
H	2.937414000	-6.167567000	-0.504114000	C	3.207683000	-0.119051000	0.794159000
H	2.307846000	-6.816453000	1.032495000	C	3.646061000	1.190293000	1.087291000
H	3.931779000	-6.087014000	0.968359000	C	4.724334000	1.718813000	0.369760000
H	1.560190000	-5.225192000	2.880193000	H	5.069901000	2.732798000	0.586731000
H	1.688738000	-3.463228000	2.659513000	C	5.359660000	1.000743000	-0.647836000
H	3.152774000	-4.434504000	2.925910000	C	6.502961000	1.618589000	-1.433646000
H	6.918057000	-1.058243000	-0.020199000	H	6.695014000	2.612390000	-0.993012000
H	8.554390000	-2.940365000	0.063728000	C	6.115291000	1.825015000	-2.908076000
H	7.353968000	-3.195348000	-1.232913000	H	5.205349000	2.435975000	-2.994534000
H	7.220559000	-4.100342000	0.290697000	H	5.917025000	0.859778000	-3.398935000
H	6.255321000	-1.054781000	2.390425000	H	6.925539000	2.325164000	-3.460950000
H	7.903214000	-1.691091000	2.175679000	C	7.793913000	0.792453000	-1.310559000
H	6.554772000	-2.801964000	2.491074000	H	7.664274000	-0.208753000	-1.749455000
H	1.593023000	-0.947099000	-2.445379000	H	8.081348000	0.661722000	-0.257262000
H	2.603820000	-0.957074000	-4.735951000	H	8.625148000	1.284442000	-1.838780000
H	2.657026000	-2.571564000	-3.988091000	C	4.898260000	-0.287096000	-0.937796000
H	4.131616000	-1.573990000	-4.053316000	H	5.379171000	-0.860295000	-1.733570000
H	2.523941000	1.142999000	-3.246253000	C	3.834614000	-0.865225000	-0.233694000
H	4.175248000	0.680545000	-2.813718000	C	3.414687000	-2.300911000	-0.524713000
H	3.001313000	1.002252000	-1.532045000	H	2.403053000	-2.435620000	-0.112144000
Sn	0.000001000	1.337839000	-0.942568000	C	4.340755000	-3.280542000	0.217894000
C	0.000000000	2.664265000	0.886351000	H	5.379540000	-3.173929000	-0.131932000
C	1.211900000	3.175820000	1.393833000	H	4.024947000	-4.321487000	0.047809000
C	1.204776000	4.148988000	2.406355000	H	4.325971000	-3.089245000	1.300330000
C	0.000001000	4.630663000	2.917964000	C	3.346628000	-2.625030000	-2.022620000
C	-1.204775000	4.148988000	2.406355000	H	2.688069000	-1.917341000	-2.548376000
C	-1.211900000	3.175820000	1.393832000	H	2.947675000	-3.639849000	-2.171590000
C	-2.535165000	2.772649000	0.824609000	H	4.338990000	-2.590802000	-2.497867000
C	-3.082459000	3.514566000	-0.246903000	C	2.951658000	2.009270000	2.163915000
C	-4.388748000	3.235837000	-0.669470000	H	1.950889000	1.568565000	2.297176000
C	-5.156458000	2.232531000	-0.071968000	C	2.759177000	3.478610000	1.764229000
C	-4.569202000	1.467067000	0.941524000	H	2.276390000	3.544799000	0.778374000
C	-3.274445000	1.718201000	1.407354000	H	3.716013000	4.021529000	1.718584000
C	-2.690511000	0.910482000	2.555274000	H	2.126127000	3.993577000	2.503119000
C	-3.039645000	1.545246000	3.913671000	C	3.699710000	1.894754000	3.503971000
H	-2.603822000	0.957074000	4.735951000	H	4.722838000	2.291947000	3.411423000
H	-2.657027000	2.571564000	3.988091000	H	3.771709000	0.847617000	3.829646000
H	-4.131618000	1.573990000	4.053315000	H	3.181135000	2.463931000	4.291356000
C	-3.119890000	-0.558541000	2.530123000	H	1.414425000	1.883776000	-0.561339000
H	-2.523943000	-1.142999000	3.246254000				
H	-4.175249000	-0.680544000	2.813717000	Ar*SnH(NMe3)			
H	-3.001313000	-1.002252000	1.532045000	N	-1.056822000	1.037250000	-2.554878000
H	-1.593023000	0.947100000	2.445380000	Sn	0.459863000	-0.463428000	-1.270447000
H	-5.150493000	0.657204000	1.386301000	C	0.108671000	0.408411000	0.830551000
C	-6.610701000	2.008430000	-0.449905000	C	-1.109112000	0.496515000	1.547469000

C	-7.489141000	3.125138000	0.144108000	C	-2.424327000	0.142647000	0.925661000
H	-8.554390000	2.940368000	-0.063730000	C	-3.436967000	1.114877000	0.736842000
H	-7.353967000	3.195351000	1.232910000	C	-3.260003000	2.569499000	1.157947000
H	-7.220558000	4.100343000	-0.290700000	H	-2.182305000	2.742796000	1.307583000
C	-6.840608000	1.880538000	-1.961794000	C	-3.771827000	3.572643000	0.108950000
H	-6.255321000	1.054779000	-2.390425000	H	-3.470558000	4.594167000	0.384921000
H	-7.903214000	1.691092000	-2.175680000	H	-4.870470000	3.563389000	0.047322000
H	-6.554770000	2.801962000	-2.491076000	H	-3.380484000	3.359174000	-0.893827000
H	-6.918058000	1.058245000	0.020199000	C	-3.973217000	2.840255000	2.497042000
H	-4.817577000	3.827704000	-1.481823000	H	-3.818340000	3.882696000	2.814961000
C	-2.295202000	4.634506000	-0.914179000	H	-3.609635000	2.181733000	3.295826000
C	-2.903454000	6.007176000	-0.583113000	H	-5.056363000	2.672505000	2.391631000
H	-2.937410000	6.167566000	0.504115000	C	-4.659119000	0.725189000	0.167824000
H	-2.307844000	6.816453000	-1.032494000	H	-5.438705000	1.474995000	0.010971000
H	-3.931777000	6.087015000	-0.968357000	C	-4.910658000	-0.588422000	-0.227549000
C	-2.169728000	4.426600000	-2.431144000	C	-6.221766000	-0.964433000	-0.893749000
H	-1.560189000	5.225192000	-2.880193000	H	-6.857979000	-0.062372000	-0.883986000
H	-1.688739000	3.463228000	-2.659514000	C	-6.962480000	-2.071015000	-0.125608000
H	-3.152774000	4.434506000	-2.925910000	H	-7.134066000	-1.779736000	0.920567000
H	-1.277983000	4.617668000	-0.494204000	H	-6.383695000	-3.007180000	-0.123107000
H	-2.155285000	4.544148000	2.771779000	H	-7.937085000	-2.282737000	-0.590951000
H	0.000001000	5.394889000	3.697494000	C	-5.996053000	-1.365358000	-2.362150000
H	2.155286000	4.544147000	2.771780000	H	-6.950678000	-1.600331000	-2.857236000
C	2.535165000	2.772648000	0.824610000	H	-5.351841000	-2.255579000	-2.428671000
C	3.082460000	3.514565000	-0.246902000	H	-5.507612000	-0.554746000	-2.922391000
C	4.388749000	3.235836000	-0.669470000	C	-3.911554000	-1.542930000	-0.002648000
C	5.156458000	2.232530000	-0.071967000	H	-4.099852000	-2.582311000	-0.276905000
C	4.569202000	1.467066000	0.941524000	C	-2.686359000	-1.212563000	0.583533000
C	3.274445000	1.718200000	1.407354000	C	-1.714919000	-2.321057000	0.971063000
C	2.690511000	0.910481000	2.555274000	H	-0.700632000	-1.898531000	0.946162000
C	3.119889000	-0.558543000	2.530123000	C	-1.728278000	-3.526841000	0.026894000
H	3.001312000	-1.002253000	1.532045000	H	-2.675304000	-4.086050000	0.079593000
H	4.175248000	-0.680546000	2.813717000	H	-0.923158000	-4.223297000	0.302333000
H	2.523941000	-1.143000000	3.246253000	H	-1.560473000	-3.215340000	-1.014467000
C	3.039645000	1.545245000	3.913671000	C	-1.983670000	-2.751241000	2.425492000
H	2.657027000	2.571562000	3.988092000	H	-1.889074000	-1.898233000	3.111990000
H	2.603821000	0.957072000	4.735951000	H	-1.264223000	-3.523889000	2.737444000
H	4.131617000	1.573988000	4.053316000	H	-2.999506000	-3.163975000	2.527302000
H	1.593023000	0.947099000	2.445380000	C	-1.102456000	0.840679000	2.911455000
H	5.150494000	0.657203000	1.386302000	H	-2.047674000	0.865907000	3.456126000
C	6.610702000	2.008429000	-0.449905000	C	0.087487000	1.114255000	3.583560000
C	6.840609000	1.880536000	-1.961793000	H	0.071967000	1.383174000	4.641424000
H	6.255322000	1.054777000	-2.390423000	C	1.297566000	0.999010000	2.900219000
H	6.554770000	2.801960000	-2.491076000	H	2.244621000	1.162638000	3.419730000
H	7.903214000	1.691089000	-2.175679000	C	1.309438000	0.627093000	1.548778000
C	7.489142000	3.125138000	0.144108000	C	2.618043000	0.350900000	0.877976000
H	8.554390000	2.940367000	-0.063730000	C	3.360945000	1.392107000	0.281524000

H	7.220558000	4.100342000	-0.290701000	C	4.565652000	1.083071000	-0.357485000
H	7.353968000	3.195351000	1.232910000	H	5.146519000	1.881485000	-0.826355000
H	6.918059000	1.058244000	0.020201000	C	5.037618000	-0.229828000	-0.452746000
H	4.817578000	3.827703000	-1.481822000	C	6.326277000	-0.537335000	-1.194868000
C	2.295203000	4.634506000	-0.914178000	H	6.746428000	0.429367000	-1.522698000
C	2.169729000	4.426600000	-2.431144000	C	6.053005000	-1.381788000	-2.451537000
H	1.560191000	5.225192000	-2.880193000	H	5.329552000	-0.882768000	-3.112135000
H	3.152775000	4.434505000	-2.925909000	H	5.635263000	-2.363362000	-2.179584000
H	1.688740000	3.463228000	-2.659514000	H	6.981199000	-1.554681000	-3.017758000
C	2.903456000	6.007175000	-0.583113000	C	7.363314000	-1.216379000	-0.285186000
H	2.937412000	6.167566000	0.504115000	H	7.002956000	-2.197616000	0.059939000
H	3.931779000	6.087014000	-0.968357000	H	7.571544000	-0.604047000	0.604056000
H	2.307846000	6.816452000	-1.032494000	H	8.309340000	-1.378250000	-0.824223000
H	1.277984000	4.617668000	-0.494204000	C	4.281157000	-1.250115000	0.131818000
H	1.210230000	0.000000000	0.000000000	H	4.635833000	-2.281130000	0.068318000
				C	3.081472000	-0.986057000	0.805706000
Ar*SnH(Pyridine)				C	2.343549000	-2.111446000	1.521942000
N	-1.312427000	1.799972000	-1.421867000	H	1.312453000	-1.768914000	1.698845000
Sn	0.508847000	0.201414000	-1.252960000	C	2.979607000	-2.357289000	2.901979000
C	-2.553671000	1.403324000	-1.759666000	H	4.027573000	-2.677977000	2.794718000
H	-2.738203000	0.327766000	-1.746146000	H	2.433305000	-3.140956000	3.449116000
C	-3.553469000	2.309608000	-2.103818000	H	2.965593000	-1.440806000	3.508726000
H	-4.547918000	1.941187000	-2.353626000	C	2.265102000	-3.409178000	0.707852000
C	-3.257735000	3.672950000	-2.113604000	H	1.817591000	-3.228753000	-0.281421000
C	-1.971043000	4.083889000	-1.757561000	H	1.643505000	-4.148468000	1.235068000
H	-1.696779000	5.138733000	-1.737704000	H	3.256613000	-3.863133000	0.558429000
C	-1.028817000	3.118901000	-1.410477000	C	2.849103000	2.823137000	0.323220000
H	-0.008452000	3.370119000	-1.115972000	H	1.769834000	2.765198000	0.536757000
C	0.308850000	-0.216740000	0.977703000	C	3.017901000	3.553151000	-1.016741000
C	-0.873104000	-0.563528000	1.663717000	H	2.597702000	2.950912000	-1.835576000
C	-2.198469000	-0.584096000	0.972310000	H	4.076252000	3.752008000	-1.245453000
C	-3.171184000	0.399061000	1.252851000	H	2.501556000	4.524944000	-0.989814000
C	-2.892285000	1.531442000	2.230584000	C	3.512439000	3.608462000	1.468251000
H	-1.817566000	1.502890000	2.464710000	H	4.602202000	3.663747000	1.319283000
C	-3.201822000	2.914435000	1.640173000	H	3.328498000	3.126302000	2.438610000
H	-2.955074000	3.702246000	2.368011000	H	3.120821000	4.636521000	1.516039000
H	-4.267097000	3.021482000	1.384527000	H	1.574987000	0.881262000	-1.771852000
H	-2.616145000	3.098315000	0.730907000	C	-2.429916000	0.506536000	-2.637757000
C	-3.666418000	1.315926000	3.543171000	H	-3.062337000	1.152957000	-3.275255000
H	-3.429969000	2.108876000	4.269097000	H	-2.402340000	-0.503967000	-3.065584000
H	-3.415828000	0.347884000	3.998916000	H	-2.867915000	0.454604000	-1.636773000
H	-4.752907000	1.330984000	3.363612000	C	-1.030264000	2.361536000	-1.910720000
C	-4.426982000	0.311893000	0.635266000	H	0.008267000	2.710853000	-1.862976000
H	-5.182519000	1.073393000	0.848738000	H	-1.643808000	3.088923000	-2.476329000
C	-4.746327000	-0.715366000	-0.254590000	H	-1.413920000	2.273022000	-0.888770000
C	-6.127457000	-0.798451000	-0.881087000	C	-0.459992000	1.113642000	-3.900681000
H	-6.645358000	0.147352000	-0.643012000	H	-0.455082000	0.113694000	-4.355853000

C	-6.939705000	-1.947764000	-0.257435000
H	-7.009720000	-1.834089000	0.833756000
H	-6.459594000	-2.916798000	-0.463450000
H	-7.959740000	-1.979037000	-0.670281000
C	-6.073114000	-0.936204000	-2.410726000
H	-7.086687000	-0.926118000	-2.838948000
H	-5.595963000	-1.881726000	-2.709030000
H	-5.499534000	-0.117878000	-2.870598000
C	-3.766138000	-1.678917000	-0.525429000
H	-3.997954000	-2.496354000	-1.210883000
C	-2.500523000	-1.637802000	0.071371000
C	-1.509293000	-2.769037000	-0.167600000
H	-0.502947000	-2.363948000	0.013533000
C	-1.527897000	-3.315663000	-1.598977000
H	-2.472317000	-3.829378000	-1.836639000
H	-0.716935000	-4.046838000	-1.730094000
H	-1.373554000	-2.508764000	-2.330517000
C	-1.737894000	-3.889123000	0.863646000
H	-1.642190000	-3.504023000	1.888640000
H	-0.999618000	-4.694524000	0.729381000
H	-2.744444000	-4.321847000	0.751784000
C	-0.821097000	-0.964570000	3.010559000
H	-1.747867000	-1.236456000	3.521441000
C	0.395669000	-1.036749000	3.688542000
H	0.423855000	-1.348448000	4.734362000
C	1.578679000	-0.741203000	3.008512000
H	2.543497000	-0.836447000	3.512240000
C	1.538150000	-0.349998000	1.663394000
C	2.807092000	-0.155473000	0.895074000
C	3.474435000	1.088632000	0.901796000
C	4.639497000	1.234689000	0.141473000
H	5.162043000	2.194609000	0.137076000
C	5.144573000	0.196433000	-0.647030000
C	6.389323000	0.401413000	-1.492421000
H	6.755961000	1.421113000	-1.281890000
C	6.059435000	0.320713000	-2.992960000
H	5.278135000	1.045014000	-3.264528000
H	5.692091000	-0.682366000	-3.259101000
H	6.952728000	0.526180000	-3.602792000
C	7.505735000	-0.586624000	-1.116258000
H	7.199803000	-1.624020000	-1.321262000
H	7.754141000	-0.515468000	-0.047465000
H	8.417981000	-0.385608000	-1.698655000
C	4.460256000	-1.023079000	-0.655049000
H	4.839114000	-1.843975000	-1.267753000
C	3.301088000	-1.223456000	0.105148000
C	2.636651000	-2.594529000	0.141270000
H	-1.039191000	1.799210000	-4.546963000
H	0.574476000	1.469588000	-3.815136000

H	1.608262000	-2.450817000	0.506808000
C	3.355679000	-3.498088000	1.159011000
H	4.404301000	-3.657949000	0.863235000
H	2.862631000	-4.480184000	1.225549000
H	3.350893000	-3.042473000	2.159385000
C	2.546060000	-3.267629000	-1.234095000
H	2.039154000	-2.614436000	-1.960299000
H	1.973902000	-4.204580000	-1.158652000
H	3.538884000	-3.522745000	-1.635115000
C	2.929907000	2.248376000	1.720477000
H	1.864840000	2.033233000	1.901767000
C	3.010526000	3.590612000	0.980254000
H	2.558135000	3.503533000	-0.018308000
H	4.050185000	3.932401000	0.860037000
H	2.473968000	4.368654000	1.544476000
C	3.633696000	2.328297000	3.086817000
H	4.712295000	2.508978000	2.956646000
H	3.513573000	1.392580000	3.650520000
H	3.219438000	3.149287000	3.692564000
H	1.432474000	1.745565000	-1.000222000
H	-4.019921000	4.405414000	-2.383740000

Literature

1. B. Schiemenz and P. P. Power, *Organometallics*, 1996, 15, 958-964.
2. B. E. Eichler, L. Pu, M. Stender and P. P. Power, *Polyhedron*, 2001, 20, 551-556.
3. M. Saito, H. Hashimoto, T. Tajima and M. Ikeda, *J. Organomet. Chem.*, 2007, 692, 2729-2735.
4. P. Jutzi, T. Heidemann, B. Neumann and H. G. Stammler, *Synthesis*, 1992, 1992, 1096-1098.
5. SAINT, APEX2, Bruker AXS Inc.: Madison, WI.
6. G. M. Sheldrick, University of Göttingen, Göttingen, Germany, 1997.
7. G. M. Sheldrick, SADABS, 2008 University of Göttingen, Göttingen, Germany.
8. C. B. Hübschle, G. M. Sheldrick and B. Dittrich, *J. Appl. Crystallogr.*, 2011, 44, 1281-1284.
9. L. J. Farrugia, *J. Appl. Crystallogr.*, 1999, 32, 837-838.
10. B. P. Johnson, S. Almstätter, F. Dielmann, M. Bodensteiner and M. Scheer, *Z. Anorg. Allg. Chem.*, 2010, 636, 1275-1285.
11. M. J. Frisch, G. W. Trucks, H. B. Schlegel, G. E. Scuseria, M. A. Robb, J. R. Cheeseman, G. Scalmani, V. Barone, B. Mennucci, G. A. Petersson, H. Nakatsuji, M. Caricato, X. Li, H. P. Hratchian, A. F. Izmaylov, J. Bloino, G. Zheng, J. L. Sonnenberg, M. Hada, M. Ehara, K. Toyota, R. Fukuda, J. Hasegawa, M. Ishida, T. Nakajima, Y. Honda, O. Kitao, H. Nakai, T. Vreven, J. A. Montgomery Jr., J. E. Peralta, F. Ogliaro, M. J. Bearpark, J. Heyd, E. N. Brothers, K. N. Kudin, V. N. Staroverov, R. Kobayashi, J. Normand, K. Raghavachari, A. P. Rendell, J. C. Burant, S. S. Iyengar, J. Tomasi, M. Cossi, N. Rega, N. J. Millam, M. Klene, J. E. Knox, J. B. Cross, V. Bakken, C. Adamo, J. Jaramillo, R. Gomperts, R. E. Stratmann, O. Yazyev, A. J. Austin, R. Cammi, C. Pomelli, J. W. Ochterski, R. L. Martin, K. Morokuma, V. G. Zakrzewski, G. A. Voth, P. Salvador,

J. J. Dannenberg, S. Dapprich, A. D. Daniels, Ö. Farkas, J. B. Foresman, J. V. Ortiz, J. Cioslowski and D. J. Fox, Gaussian, Inc., Wallingford, CT, USA, 2009.
12. A. D. Becke Phys. Rev. A, 1988, 38, 3098
13. J. P. Perdew, Phys. Rev. B, 1986, 33, 8822-24.
14. F. Weigend and R. Ahlrichs, *Phys. Chem. Chem. Phys.*, 2005, 7, 3297-3305.
15. F. Weigend, *Phys. Chem. Chem. Phys.*, 2006, 8, 1057-1065.
16. S. Grimme, S. Ehrlich and L. Goerigk, *J. Comput. Chem.*, 2011, 32, 1456-1465.
17. C. P. Sindlinger and L. Wesemann, *Chem. Sci.*, 2014, 5, 2739-2746.
18. J. M. Tao, J. P. Perdew, V. N. Staroverov, and G. E. Scuseria, Phys. Rev. Lett., 2003, 91, 146401.
19. M. M. Francl, W. J. Pietro, W. J. Hehre, J. S. Binkley, D. J. DeFrees, J. A. Pople, and M. S. Gordon, J. Chem. Phys.,1982, 77, 3654-65.
20. Y. Liang, S. Liu, Y. Xia, Y. Li and Z.-X. Yu, *Chem. Eur. J.*, 2008, 14, 4361-4373.
21. L. Zhao, F. Huang, G. Lu, Z.-X. Wang and P. v. R. Schleyer, *J. Am. Chem. Soc.*, 2012, 134, 8856-8868.
22. R. E. Plata and D. A. Singleton, *J. Am. Chem. Soc.*, 2015, 137, 3811-3826.
